# Phase I Study of Safety and Immunogenicity of an *Escherichia coli*-Derived Recombinant Protective Antigen (rPA) Vaccine to Prevent Anthrax in Adults

**DOI:** 10.1371/journal.pone.0013849

**Published:** 2010-11-05

**Authors:** Bruce K. Brown, Josephine Cox, Anita Gillis, Thomas C. VanCott, Mary Marovich, Mark Milazzo, Tanya Santelli Antonille, Lindsay Wieczorek, Kelly T. McKee, Karen Metcalfe, Raburn M. Mallory, Deborah Birx, Victoria R. Polonis, Merlin L. Robb

**Affiliations:** 1 United States Military HIV Research Program, Henry M. Jackson Foundation, Rockville, Maryland, United States of America; 2 United States Military HIV Research Program, Walter Reed Army Institute of Research, Rockville, Maryland, United States of America; 3 DynPort Vaccine Company LLC, Frederick, Maryland, United States of America; The George Washington University Medical Center, United States of America

## Abstract

**Background:**

The fatal disease caused by *Bacillus anthracis* is preventable with a prophylactic vaccine. The currently available anthrax vaccine requires a lengthy immunization schedule, and simpler and more immunogenic options for protection against anthrax are a priority for development. In this report we describe a phase I clinical trial testing the safety and immunogenicity of an anthrax vaccine using recombinant *Escherichia coli*-derived, *B. anthracis* protective antigen (rPA).

**Methodology/Principal Findings:**

A total of 73 healthy adults ages 18–40 were enrolled and 67 received 2 injections separated by 4 weeks of either buffered saline placebo, or rPA formulated with or without 704 µg/ml Alhydrogel® adjuvant in increasing doses (5, 25, 50, 100 µg) of rPA. Participants were followed for one year and safety and immunologic data were assessed. Tenderness and warmth were the most common post-injection site reactions. No serious adverse events related to the vaccine were observed. The most robust humoral immune responses were observed in subjects receiving 50 µg of rPA formulated with Alhydrogel® with a geometric mean concentration of anti-rPA IgG antibodies of 283 µg/ml and a toxin neutralizing geometric 50% reciprocal geometric mean titer of 1061. The highest lymphoproliferative peak cellular response (median Lymphocyte Stimulation Index of 29) was observed in the group receiving 25 µg Alhydrogel®-formulated rPA.

**Conclusions/Significance:**

The vaccine was safe, well tolerated and stimulated a robust humoral and cellular response after two doses.

**Trial Registration:**

ClinicalTrials.gov NCT00057525

## Introduction

*Bacillus anthracis* is a gram positive, facultative anaerobic, rod-shaped bacterium that has the ability to form endospores. Due to their durability, the endospores have the potential to be weaponized and therefore pose a threat for use by terrorists and/or adversary governments. *B. anthracis* produces a tripartite toxin composed of: protective antigen (PA), edema factor (EF), and lethal factor (LF). The binding of PA to the target cell initiates a sequence of events that result in EF and LF accessing the cytosol of the target cell, eventually culminating in cell death [1]. The bio-terrorism attacks in 2001 involving spore-laden envelopes mailed to individuals in the U.S. Capitol building and elsewhere have reinforced the need for vaccination strategies to protect against anthrax exposures.

The currently licensed anthrax vaccine in the U.S., BioThrax™ (previously called anthrax vaccine adsorbed, or AVA), protects against inhalation anthrax in monkeys and rabbits [2], [3], [4] and a prior version of the vaccine conferred protection from occupational exposure in humans [5]. Biothrax™ is a cell-free filtrate and while the components of the vaccine have not been fully elucidated, the major immunogen in this vaccine is PA [6]. It has been well documented in animal challenge studies that antibodies against PA lead to protection from anthrax exposure [7]. The next generation of vaccines has focused on using rPA expressed in prokaryotic systems such as *B. anthracis* and *Escherichia coli* (*E. coli*) as the sole immunogen.

The primary immunization schedule for BioThrax™ in humans is time intensive, requiring up to 5 injections spaced over an 18 month period [8]. Although determined to be safe by the FDA [9] and more recently by the VAERS Working Group [10], there is still controversy surrounding the safety profile of this product. Specifically, local and systemic reactions to the vaccine are reported to be more common and of longer duration compared to other experimental rPA vaccines [11], [12]. These drawbacks, real or perceived, have stimulated a desire to develop new vaccines to prevent anthrax. These new vaccines would likely be available to U.S. defense forces, to emergency support personnel, as well as to the general public, for protection from potential bioweapon attacks.

Currently, there are two reports of *B. anthracis* expressed rPA vaccines in humans [13], [14]. Similar humoral responses to the rPA vaccines were reported in both of these studies, despite differences in the amount of adjuvant used and the number of vaccinations. Herein, we report the results of a randomized phase I clinical trial of an *E. coli* expressed rPA vaccine administered to humans. The humoral responses we observed following administration of two injections of the current rPA vaccine were found to be similar to those previously reported [13], [14]. In addition, we provide data for the first time on the cellular immune response to rPA in humans.

## Materials and Methods

The protocol for this trial and supporting CONSORT checklist are available as supporting information; see Checklist S1 and Protocol S1.

### Objectives

This study aimed to assess the safety and immunogenicity of an anthrax vaccine in which a recombinant *Escherichia coli*-derived, *B. anthracis* protective antigen is the principal antigenic component.

### Participants and Randomization

**Figure 1:**
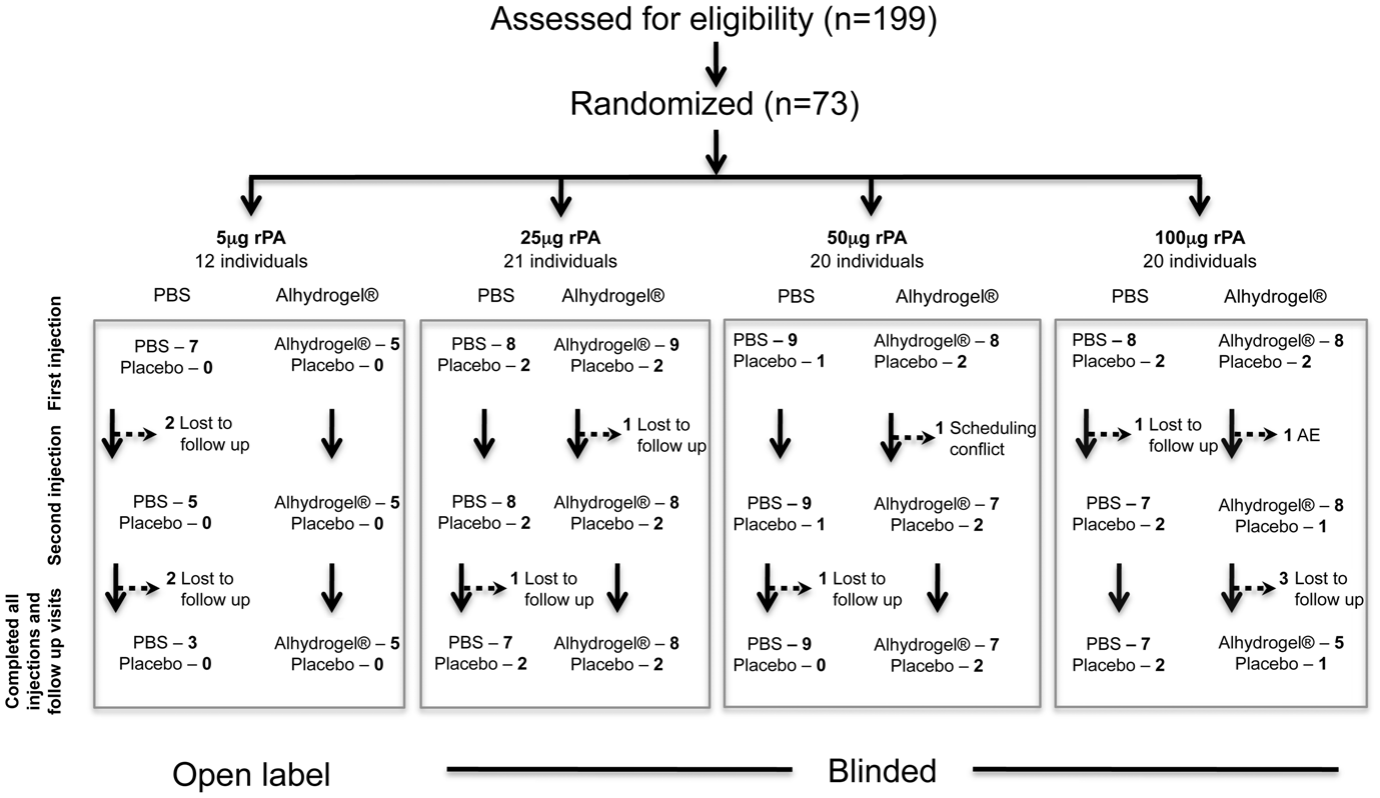
CONSORT flowchart.

The study enrolled 73 healthy adults age 18 to 40 years who were anthrax vaccine naïve. If any participants were unable to complete the vaccine schedule for any reason, they were replaced within the study recruitment time period. The inclusion and exclusion criteria are described in detail in the included trial protocol. Briefly, individuals were required to be between 18 and 40 years of age, and in good health. Individuals who had chronic medical or psychiatric illness, required immune modulators, reported drug or alcohol abuse or were unable to meet all required protocol visits were excluded. The first 12 participants received 5 µg of active vaccine with or without adjuvant under open label (i.e., not blinded) to test initial safety; the remaining study subjects were blinded to receipt of vaccine or placebo. For the blinded portion of the study, the study statistician prepared a randomization list using the RANUNI function in SAS Version 8 (SAS Institute, Cary, NC USA); only the study pharmacist and the statistician had access to this randomization list, and study personnel were unblinded only after study completion. The study plan is diagramed in a CONSORT flowchart (Figure 1). Safety data was analyzed and reported on every subject (73 total) at every available time point immediately after initial injection. These include immediate reactions and at 48–72 hours and 2 weeks post each injection. Longer term safety and immune response data was collected at visits 6, 10, 16, 26, 36, and 52 weeks after the initial injection. Serious adverse events were solicited at all time points through the 52 week visit. All adverse and serious adverse events were graded on a scale that is used for rating adverse events associated with vaccines, as recommended by the Division of Acquired Immunodeficiency Syndrome of the National Institute of Allergy and Infectious Diseases (http://rcc.tech-res.com/safetyandpharmacovigilance). Immunogenicity data was analyzed and reported on all subjects who provided a sample at the time point being assessed. Two subjects were excluded from the immunogenicity analyses; one subject who was randomized to receive placebo inadvertently was administered vaccine product (50 µg of rPA with PBS) at the second inoculation and thus was included in safety analysis, but not immunogenicity analysis. One subject, randomized to the 100 µg rPA with PBS arm, had prior undisclosed anthrax vaccine exposure as determined by detectable levels of anti-rPA antibodies prior to injection of vaccine. This volunteer denied anthrax vaccine exposure at entry and after unblinding reported never being aware of receiving an anthrax vaccine. This subject was also included in safety analysis, but excluded from the immunogenicity analysis. The samples and data were collected in Rockville MD USA at the Rockville Vaccine Assessment Clinic (RVAC).

### Trial Design

Subjects were enrolled into four sequential, escalating dosage groups (5, 25, 50 or 100 µg of rPA) formulated in phosphate buffered saline (PBS) or Alhydrogel® adjuvant resulting in 8 dosage/formulation groups as well as a placebo group (Figure 1). The volunteers enrolled in the initial, open-label, phase of the trial received vaccine containing the lowest antigen concentration (5 µg) under study. Within the subsequent double-blind, placebo-controlled portion of the study (25, 50 or 100 µg doses of rPA formulated with PBS or Alhydrogel®), 10–11 volunteers were randomized at each antigen dosage level to receive placebo (PBS alone) or active agent (rPA) at one of the two formulations in a 1∶4 ratio. Each vaccine or placebo was administered as two intramuscular injections in the left deltoid four weeks apart. Safety assessment included a diary of local reactions such as pain, tenderness, or warmth and systemic reactions such as fatigue, headache, or diarrhea for one week post-immunization which was used as memory aid to identify solicited adverse events (post-injection reactions, PIRs). The PIRs were recorded separate from other adverse events (AEs) unless a PIR persisted beyond one week post-injection. Also, at each of the 13 clinic scheduled visits, samples were collected for hematology, serum chemistry, urinalysis and immunogenicity.

### Vaccine

The rPA component of the vaccine formulations tested in the study was produced in *E. coli* by Cambrex BioScience Inc, now Lonza Biologics Inc. (Hopkinton, MA). The bulk rPA was formulated (25 mM Sodium Phosphate, 150 mM Sodium Chloride, pH 8.0) and vialed at Walter Reed Army Institute of Research (WRAIR) Pilot BioProduction Facility, (Forest Glen, MD). In addition, empty vials and vials containing diluent used to prepare vaccine formulations were processed at the same facility. Alhydrogel® is an aluminum hydroxide preparation manufactured under cGMP by HCI BioSector (Frederikssund, Denmark). For vaccine formulations containing adjuvant, rPA was adsorbed to 1.3% Alhydrogel® (w/v) resulting in formulations containing 704 µg of elemental aluminum per dose.

### ELISA

The validated ELISA was performed with a modified version of the protocol generously provided by Conrad Quinn and reported previously [15]. Immulon 2 microtiter plates (Fisher Scientific, Pittsburgh, PA) were coated with rPA (2 µg/ml) (List Biological Laboratories Inc., Campbell, CA) in phosphate-buffered saline [PBS, (Quality Biological, Gaithersburg, MD), pH 7.4] overnight at 4°C. After washing the plates three times, reference standard (Anti-AVA sera AVR801, kindly provided by Conrad Quinn, CDC, Atlanta, GA), serum samples and controls were diluted in serum diluent (5% skim milk in PBS with 0.1% Tween-20, pH 7.4) and incubated in antigen-coated wells for one hour at 37°C. Plates were then washed three times with wash buffer and incubated with horseradish peroxidase (HRPO)-conjugated mouse anti-human IgG (affinity purified, gamma chain specific monoclonal antibody, clone HP6043 (Hybridoma Reagent Laboratory, Baldwin, MD) (diluted 1∶16,000 in serum diluent). After an hour incubation at 37°C, plates were washed three times, after which substrate (ABTS; Kirkgaard & Perry, Gaithersburg, MD) was added and plates were incubated for 30 minutes at 37°C. The reactions were stopped and optical densities were read using a Molecular Devices Vmax microplate reader with Softmax Pro software (Molecular devices, Sunnyvale, CA). The endpoint titers were calculated using software (ELISA for Windows) kindly provided by Conrad Quinn, CDC Atlanta, GA [16]. For all calculations, values that were below the lower limit of detection were given an arbitrary value of “1” and those below the limit of quantitation were given an arbitrary value of “5”.

### Toxin neutralization assay (TNA)

The validated TNA was performed using a modified version of the protocol provided by Conrad Quinn, CDC Atlanta, GA [16]. Briefly, J774A.1 cells (mouse macrophage/monocyte cells – ATCC TIB-67) were used as targets for toxicity mediated by rPA and recombinant lethal factor (rLF). A working solution of anthrax toxin with a final concentration of rPA and rLF (List Biological Laboratories Inc., Campbell, CA) at 0.1 µg/ml and 0.08 µg/ml, respectively was used in the assay. Sample sera were diluted with a six-point 2-fold dilution scheme. Anti-AVA sera pool AVR801 was used as a positive control and standard curve. Normal human serum (NHS, Sigma, MO) was used as a negative control. The working solution of toxin was added to plates that contained serially diluted sample serum and after a 30 min incubation, the toxin/sera was added to J77A.1 cells and incubated for four hrs at 37°C. Cell viability was assessed by adding 25 µl of MTT [3-(4,5-dimethylthiazol-2-yl)-2,5-diphenyltetrazolium bromide] and incubating for an additional two hrs at 37°C. Prior to addition of 100 µl Solubilization Buffer [50% N, N-Dimethylformamide (DMF) (Sigma, St. Louis, MO) with 20% SDS (Sigma, St. Louis, MO), pH 4.7], which allows for visualization of MTT color changes, media was removed from plates by gently inverting and tapping the plates onto a paper towel. Plates were read using a Molecular Devices Vmax microplate reader with Softmax Pro software. The ED_50_ for antibody-mediated protection from killing was calculated using the “c” parameter (or the mid-point) from the serum sample ODs plotted in a 4-parameter curve from the Softmax Pro software. For all calculations, values that were below the lower limit of detection were given an arbitrary value of “1” and those below the limit of quantitation were given an arbitrary value of “10”.

### Lymphocyte proliferation assay (LPA)

The LPA was modified from previously published assays [17]. Briefly, whole blood was collected in acid citrate dextrose (ACD) citrate tubes and PBMC were separated by ficoll-hypaque density centrifugation. Five million PBMC were set-aside for LPA and the remaining PBMC were cryopreserved and stored in liquid nitrogen. Triplicate wells of 100,000 fresh PBMC were incubated in 96-well U-bottom plates with the following antigens or mitogens: 1, 5 or 10 µg/ml of rPA (List Biological Laboratories Inc., Campbell, CA), 2.5 or 5 µg/ml of tetanus toxin (List Biological Laboratories Inc., Campbell, CA). In separate plates 100,000 fresh PBMC were stimulated with 2 µg/ml phytohaemagglutinin, 1.25 mg/ml pokeweed mitogen and 20 µg/ml concavalinA. After three and six days of incubation at 37°C respectively for the mitogens and antigens, cells were pulsed with one µCi/well of [3H]-thymidine for six hr. Radioactivity incorporated into dividing cells was assessed by measuring counts per minute (cpm) in a Wallac Micobeta (Perkin Elmer, Shelton, CT) scintillation counter. The data is expressed as a lymphocyte stimulation index [LSI  =  (mean cpm of stimulated cells)/(mean cpm of unstimulated cells)] to define antigen specificity. Samples were designated as positive if the LSI was greater than or equal to 5. Assays were considered invalid if there was documentation of technical failure due to lack of tritium incorporation, high background cpm, not enough cells to complete the assay or lack of response to the mitogens.

### Ethics

The study was approved by independent institutional review boards both at the Walter Reed Army Institute of Research and the Human Subjects Review Board of the US Army Medical Research and Materiel Command. Each participant gave written informed consent and completed an exam of understanding prior to engaging in any study-related procedure.

### Statistical methods

Safety assessments for all volunteers were analyzed for all time points using Fisher's exact test. Immunogenicity data were analyzed using Wilcoxon rank-sum and Kruskal-Wallis tests. Correlation between ELISA and TNA results were assessed by calculating the linear regression and applying the Spearman correlation test. Prism 5 for Mac OS X (Graphpad, La Jolla, CA) was used for all statistical calculations.

## Results

This phase I trial was designed to examine the safety and immunogenicity of rPA formulated with either in phosphate buffered saline (PBS) or Alhydrogel®. The median age of enrolled study subjects was 28 years; 51% were female. Of the 73 participants enrolled in the study, 60 (82.2%) completed all vaccinations and follow-up visits, providing samples for immunogenicity assessment (Figure 1).

### Safety and adverse events

**Figure 2:**
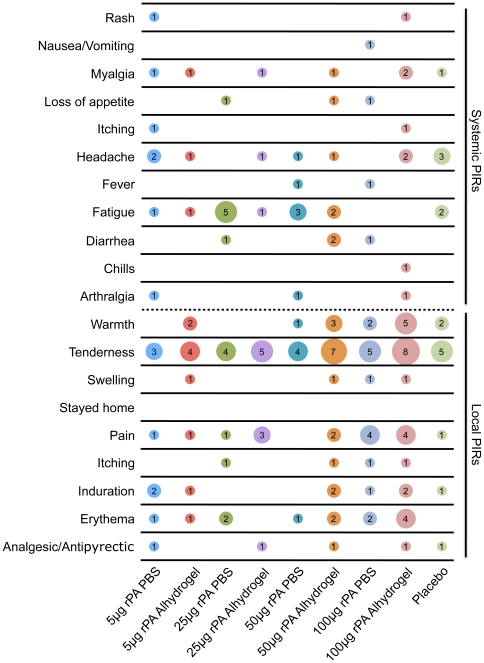
Post-injection reactions (PIRs). The X axis displays the dosage groups along with all of the placebos, who were combined into one group. The Y axis displays the PIRs that were assessed in the study. The data displayed in the matrix represents the number of individuals who reported a given PIR and the size of the circle is proportional to the reported number. The top half of the graph consists of systemic PIRs while the bottom half of the graph consists of local PIRs.

Common local and systemic adverse events associated with vaccines were solicited by clinic staff with the aid of diary cards filled out by the volunteer and referred to as “post-injection reactions” or PIRs. PIRs were defined as adverse events (AEs) that occurred within the first 6 days after vaccination, which, due to the close temporal (and as applicable, spatial) relation to injection, were regarded as probably or definitely related to inoculation as determined by the principal investigator. If any PIR had a duration that lasted beyond 6 days post vaccination, it was recorded as both a PIR and an AE. All local PIRs were of mild severity. Overall, 47/62 (76%) rPA recipients had a local PIR, the majority of these subjects (40/47: 85%) experienced tenderness (Figure 2). Warmth, pain and erythema were the next most common PIRs observed. The most common local PIRs for placebo recipients were tenderness (5/11, 45%) and warmth (2/11, 18%) (Figure 2). The rate of local reactions for placebo recipients (7/11: 64%) was not statistically different from those of vaccine recipients (Fisher's exact test, p = 0.46). Overall, 28/62 (45%) of rPA recipients and 4/11 (36%) of placebo recipients experienced a systemic PIR again with no statistical difference between rPA and placebo recipients (Fisher's exact test, p = 0.75). The most common systemic PIR was fatigue for both vaccine (13/62, 20%) and placebo (3/11, 27%) recipients (Figure 2). All systemic PIRs observed were mild except for a single febrile episode graded as “moderate” (temperature measured as 101.7°C 2 days after the first injection) in a volunteer who received 50 µg of rPA without adjuvant. There appeared to be no relationship to dose or adjuvant observed with regard to systemic reactions, however the groups receiving 50 µg of rPA with adjuvant, as well as those receiving 100 µg of rPA with or without adjuvant appeared to have the most local reactions (Figure 2). After receiving the first injection, 39% of rPA recipients had a local PIR and 40% had a systemic PIR. The percentage of rPA recipients with a local PIR went up to 58% after the second injection, while the percentage of reported systemic PIRs went down to 15%. Of the placebo recipients, 55% and 27% reported local and systemic PIRs, respectively, after the first injection. After the second injection, 27% and 9% reported local and systemic PIRs, respectively.

**Figure 3:**
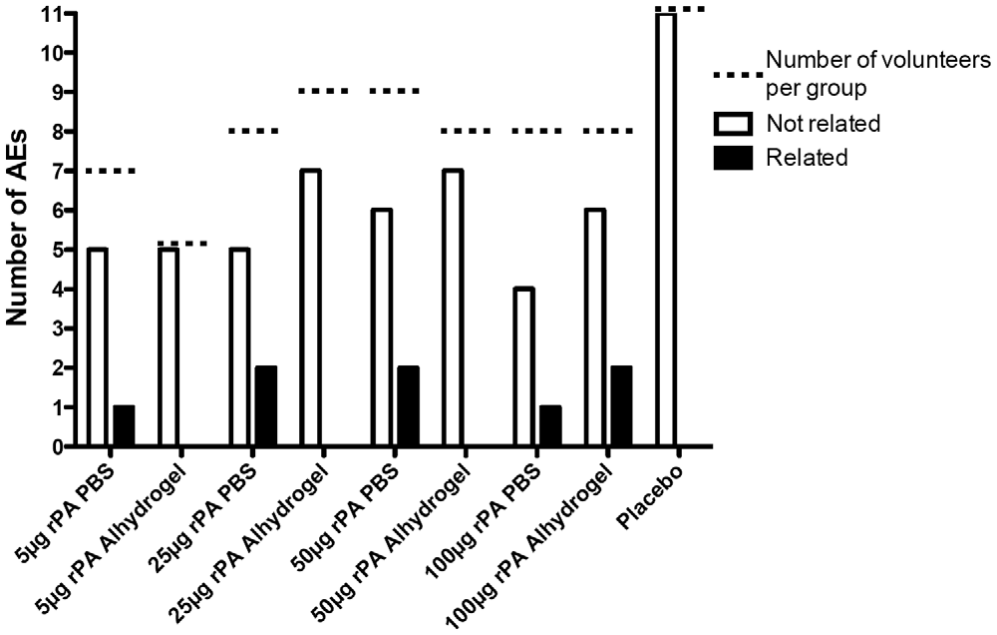
Adverse events (AEs). The number of AEs was tallied for each dosage group, as well as all of the placebos, who were combined into one group. The empty bars represent AEs that were determined to be not related to the vaccine. The filled bars represent AEs that were determined to be related to the vaccine. The dotted line indicates the total number of volunteers in each group. All AEs that were determined to be related to the vaccine were mild in severity.

There were a total of 54 out of 62 subjects who reported AEs that occurred 6 days after receipt of rPA (or PIRs that continued in duration past 6 days), however, only 8 were judged to be possibly, probably or definitely related to the vaccine and all were of mild severity (Figure 3). Of these, 3 were mild fatigue and 4 were mild, prolonged injection site reactions, the longest lasting 10 days. The remaining related AE was a case of hypoesthesia in the forearm opposite the administration arm of an individual in the 100 µg rPA/adjuvant group. Only one serious adverse event occurred, an idiopathic cardiomyopathy arising 5 months after the second vaccination with 5 µg of rPA formulated with alum, but was judged to be unrelated to the vaccine. There were AEs in 10 of 11 placebo recipients, all being mild or moderate, none judged to be related to the injection. Urine and blood collected for laboratory analysis showed no difference in the frequency of abnormalities for each analyte as a function of vaccine or dose. Of the lab abnormalities, none were graded as an AE related to the vaccine. The proportion of all lab abnormalities observed in vaccine and placebo groups were similar with 49 lab abnormalities of 407 (12%) of placebo assessments compared to 254 abnormalities of 2183 lab assessments (11.6%) in the vaccine group. Further, there was no difference in the proportion of lab abnormalities as a function of dose group or adjuvant (data not shown).

### Overall Immunogenicity

Among the participants receiving rPA, all produced binding antibody except for three participants at the 5 µg dose formulated in PBS and one participant at the 25 µg dose formulated in PBS. Considering all three assays for immunogenicity, there was only one participant who received rPA (in the 25 µg PBS formulation group) that did not respond to the immunogen. The toxin neutralization assay (TNA) was able to detect responses in sera from all vaccinees, except the previously mentioned participant. The lymphocyte proliferation assays (LPA) response rate was lower compared to the other two assays, especially at the higher concentrations of rPA (50 µg and 100 µg). No rPA specific immune responses were detected by TNA or LPA in placebo recipients (data not shown); however, detectable, but unquantifiable, levels of anti-rPA specific antibodies were found in two participants who received placebo. For one of these participants, this activity was found in a single sample obtained prior to initiation of study injections, while for the other participant, the levels of anti-rPA antibody became quantifiable at a low level (15 µg/ml of anti-rPA antibodies) transiently. However, this sample was approximately 30 days post placebo injection (data not shown), indicating that these measurements were most likely due to non-specific binding.

### Humoral Immunogenicity

**Figure 4:**
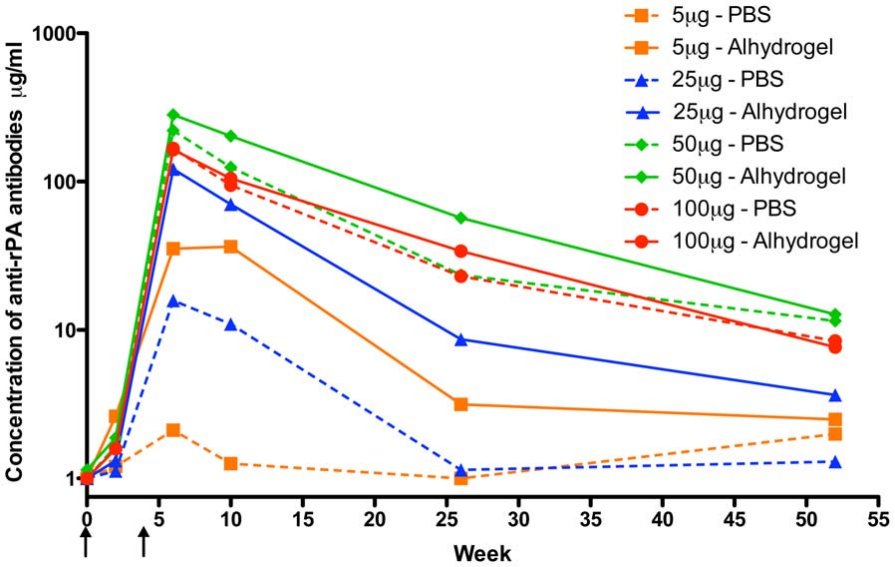
Geometric mean concentration of anti-rPA antibodies over time. The concentration of anti-rPA antibodies was assessed by ELISA at weeks 0, 2, 6, 10, 26, and 52 over the course of the entire study. Arrows indicate when the two injections occurred.

**Figure 5:**
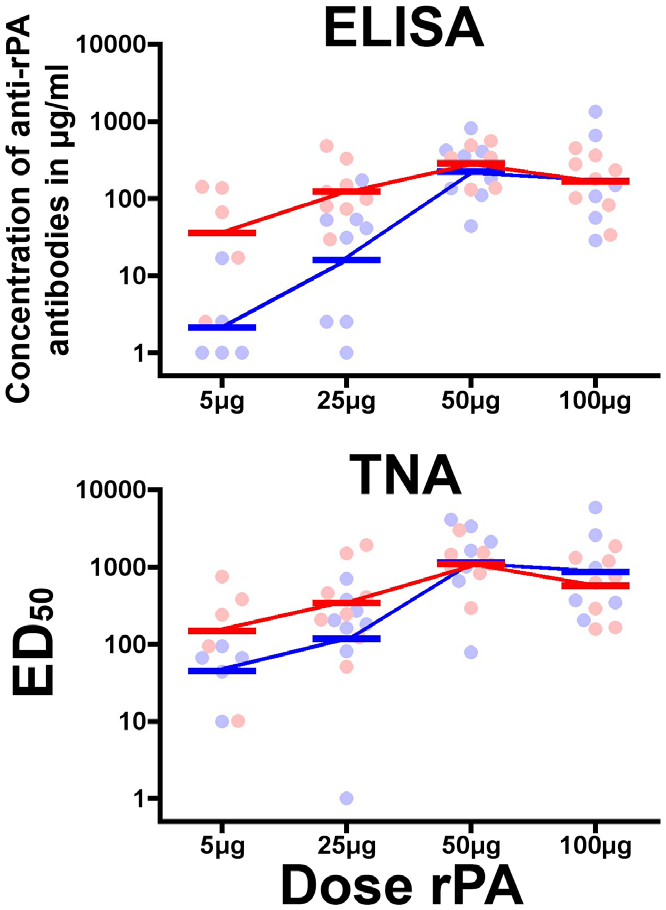
Vaccine induced humoral immunogenicity. (A) The geometric mean concentrations (GMC) of the anti-rPA antibodies and (B) the geometric mean reciprocal 50% titers (GMT) for the TNA were calculated for each group (horizontal bars) at study visit 8 (2 weeks post second vaccination). The scattergram of the individual responses displays participants who received rPA formulated without Alhydrogel® adjuvant (PBS) in blue and participants whom received rPA formulated with Alhydrogel® in red.

Anti-rPA specific antibodies responses among rPA recipients are shown in Figure 4. The peak geometric mean concentration (GMC) of anti-rPA antibodies was observed 2 weeks after the second vaccination and diminished over time to low, but detectable, levels by the final study visit. Antibody levels were quite low after the initial injection, while the second vaccination elicited a much stronger immune response. For all rPA doses, inclusion of adjuvant with the rPA stimulated GMCs equal to, and in most cases higher than GMC produced following receipt of rPA alone at 2 weeks post-second immunization. This was most pronounced in the 5 µg and 25 µg groups (Figures 4 and 5). Within the 5 µg dosage level group, the difference between adjuvanted and non-adjuvanted rPA approached statistical significance (Wilcoxon rank-sum p = 0.06) and achieved significance for the 25 µg dose group (Wilcoxon rank-sum p = 0.03). The 50 µg and 100 µg groups had similar GMCs 2 weeks after the second vaccination independent of the presence of adjuvant (Wilcoxon rank-sum p = 0.69 and 0.31 respectively). The relative difference between the two formulations was maintained for all groups through the final visit. Participants who received 5 µg rPA (both formulations) or 25 µg rPA with PBS no longer had quantifiable levels of anti-rPA antibodies at the final visit. Of the 24 volunteers within the groups who received the lower two doses of rPA, only 3 (12.5%) had both detectable and quantifiable concentrations of anti-rPA antibodies, all of whom were in the Alhydrogel®-formulated 25 µg rPA group. Of the volunteers within the groups who received the higher two doses of rPA formulated with PBS, 8 out of 14 (57%) had both detectable and quantifiable concentrations of anti-rPA antibodies at the final visit as compared to 9 out of 12 (75%) of the dose-matched group who received rPA formulated with adjuvant (data not shown). Overall, the volunteers who received either the 50 µg or 100 µg dose of rPA, regardless of formulation, had significantly higher GMCs of anti-rPA specific antibodies at the final visit than those who received 5 µg and 25 µg of rPA (Kruskal-Wallis p = 0.0063). Despite this significance, the range of GMCs of all eight groups was quite low (1.3–12.7 µg/mL).

To further assess the influence of rPA antigen dose and adjuvant inclusion on the elicitation of a humoral response, the concentrations of anti-rPA binding antibody and neutralizing antibody (TNA) effective dilution 50 (ED_50_) titers measured from participants 2 weeks after the second vaccination were plotted (Figure 5). The anti-rPA antibody titers escalated between groups receiving 5 µg to 50 µg of rPA, peaking in the group that received 50 µg; this was irrespective of rPA formulation. The Alhydrogel®-formulated rPA elicited higher anti-rPA antibody concentrations than rPA without adjuvant for the lower immunogen concentration groups (5 µg and 25 µg), however, there was no discernable difference between GMCs in the higher dosage groups (50 µg and 100 µg). A similar pattern was seen in the TNA. The geometric mean titers (GMTs) of the ED_50_s were higher at 5 µg and 25 µg of rPA doses of rPA in the Alhydrogel® receiving groups than those from the PBS groups (Figure 5). At 50 µg and 100 µg of rPA, the GMTs were almost equivalent. Similar to the ELISA, peak GMTs were observed at 50 µg.

**Figure 6:**
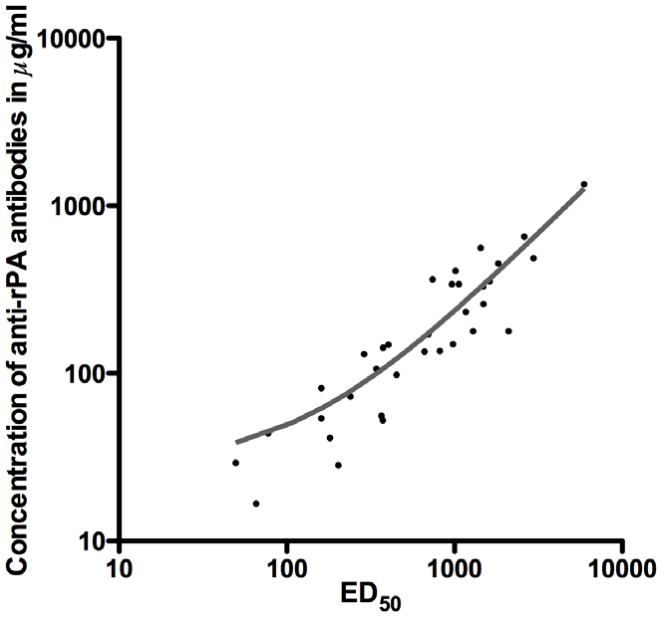
Correlation between the concentration of anti-rPA binding antibodies and the ED_50_ for the individual samples. The plot depicts the relationship between the level of anti-rPA antibody binding and the neutralization ED_50_ in the TNA. A linear regression (grey line) indicates a strong correlation between the two parameters (R^2^ = 0.86). All samples from rPA recipients are included in the linear regression calculation, however, only the data that were above the limit of quantitation for both assays are displayed.

To assess if the results obtained from the ELISA and the TNA correlated, the concentration of anti-rPA antibodies was plotted (Y axis) against the neutralizing ED_50_ (X axis) for each participant (Figure 6). The two assays were highly correlated, with a linear regression R^2^ of 0.86. Using the Spearman test for correlation, the R-value was 0.93 with a p value of <0.0001.

### Cellular Immunogenicity

**Figure 7:**
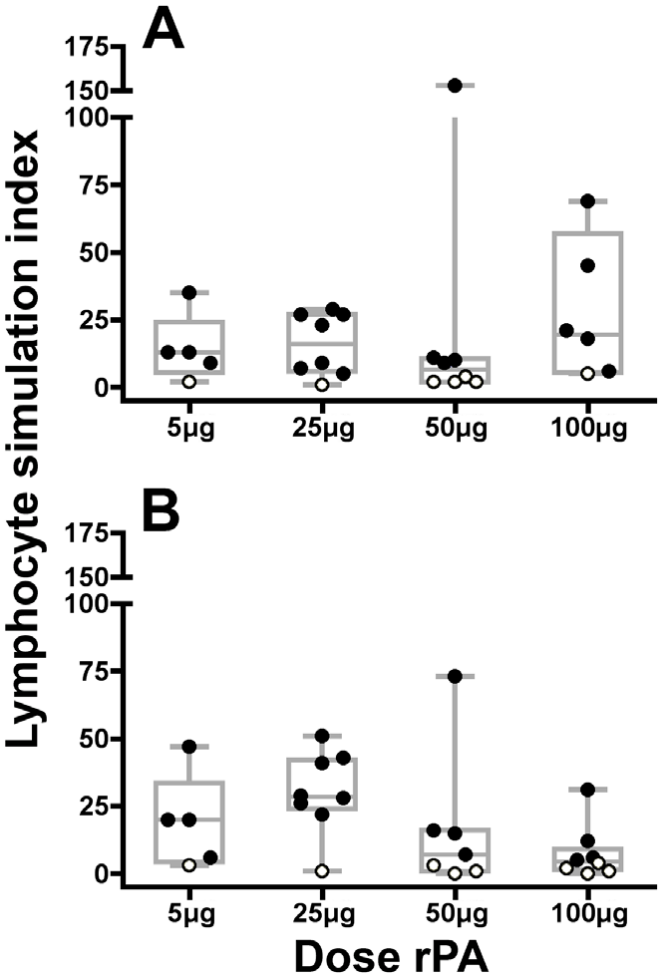
Vaccine induced lymphocyte proliferation. The LPA was performed on fresh cells obtained the day of the assay from participants at study visit 8 (2 weeks post second vaccination) incubated with 1 µg/ml of rPA. The resultant lymphocyte stimulation index (LSI) is plotted for all rPA-receiving participants. The individual data points are overlaid on box and whiskers plots, which show the median and percentiles for the data within each group. (A) Individuals whom received rPA formulated without Alhydrogel® adjuvant (PBS). (B) Individuals whom received rPA formulated with Alhydrogel®. Negative values (LSI ≤5) are denoted by open circles.

Cellular immune responses to rPA were assessed by incubating peripheral blood mononuclear cells (PBMC) with 10, 5 (data not shown) and 1 µg/ml of rPA; data are expressed as a lymphocyte stimulation index (LSI) where an LSI ≥5 is considered a positive response (closed circles). Responses to control mitogens were positive for all volunteers shown and tetanus responses were consistent across visits for each volunteer in the LPA (data not shown). Post vaccination, the median LSI to rPA ranged from 4.5 to 28.5. Two weeks after the second injection, the groups immunized with lower concentrations of rPA (5 µg/ml and 25 µg/ml) in both formulations showed a robust response to rPA with each group only having one non-responder (Figure 7). In the groups that received either 50 µg or 100 µg of rPA with Alhydrogel®, there were fewer rPA responders. While the same phenomenon was seen in the 50 µg PBS group, the 100 µg PBS group only had one non-responder. Only 2 of the 62 participants showed a proliferative response to rPA prior to vaccination with LSI of 6 and 8 respectively (data not shown).

## Discussion

This study assessed the safety of *E. coli* derived rPA in a phase I trial in humans. At the dosages and formulations tested, the vaccine was found to be safe and well tolerated. All PIRs were of mild severity, aside from one (graded moderate) febrile episode. The most common PIR reported as tenderness of the injection site (Figure 2). Among 54 rPA recipients reporting AEs, 8 were judged to be potentially related to receipt of vaccine 7 of which were general disorders and administration site conditions (Figure 3). In contrast to a previous report with a similar rPA vaccine [13], we did not observe a difference in reactogenicity between men and women (data not shown). However, the small number of study subjects in this trial precludes a robust comparison on this point.

Vaccine formulations of rPA with and without Alhydrogel® were immunogenic. The humoral responses, as measured by ELISA for binding antibodies and TNA for neutralizing antibodies, follow similar patterns (Figure 5) and the values correlate well (Figure 6), which is in line with prior observations following natural infection and vaccination with Biothax™ [13], [16], [18]. We show here, as have others [14] that formulation with Alhydrogel® tended to enhance antibody responses, especially at 5 µg and the 25 µg dosage levels where, at 2 weeks post-second vaccination (near the presumed peak of immunogenicity), the GMCs of anti-rPA antibodies were nearly a log higher in the groups receiving Alhydrogel®-formulated rPA than those in groups receiving PBS-formulated rPA (Figure 4). At later post-vaccination time points, the concentration of rPA antigen appears to influence the levels of anti-rPA antibodies more than the presence of adjuvant (Figure 5), however, due to the narrow range of GMCs for all eight groups it is unknown if there is biological relevance in terms of potential protection between these values.

The relatively rapid decrease in anti-rPA antibody titers after receipt of the second injection that we observed in this study has been recognized previously [18]. In the current Biothrax™ vaccination schedule, injections are given at 0, 4 weeks and 6, 12, and 18 months with annual boosts recommended to maintain high levels of anti-PA antibodies [8]. In humans, the previous schedule for Biothrax™ included three injections 2 weeks apart with 3 more injections occurring at 6, 12, and 18 months [19]. The fourth injection was shown to be critical for maintenance of higher levels of antibodies for prolonged periods of time [18]. However after only two Biothrax™ vaccinations, protection was still achieved with challenges two years post-vaccination in rhesus macaques [3], [20] despite relatively low levels of anti-PA antibodies present in the monkeys at the time. While efforts have been made to determine what levels of antibody are required for protection in both rabbits [21], [22] and guinea pigs [23], these values may or may not translate into protection for humans. A recent study using a monoclonal antibody (mAb) against PA in rabbits and monkeys found that a concentration of 40 mg/kg lead to 100% protection in rabbits and 90% protection in cynomolgus monkeys. While the dosage groups in our study did not reach this level of antibody, protection in the setting of a vaccine involves not only neutralizing antibodies present at the time of exposure, but also the time involved with stimulating the memory immune response. Interestingly, humans vaccinated with the United Kingdom's licensed human anthrax vaccine had measurable T cell immune responses 10–15 years after vaccination without boosts [24].

To our knowledge, this is the third published study that examines the safety and immunogenicity of a recombinant PA vaccine. Some trends become evident by comparing the studies. First, as has been noted [14], increased amounts of Alhydrogel® within the rPA vaccine formulation enhances humoral immunogenicity. Formulation of 704 µg (used in this study) or 800 µg [14] of elemental aluminum with 50 µg of rPA resulted in higher GMCs of anti-rPA binding antibodies and higher GMTs of neutralizing antibodies than formulation with 82.5 µg of Alhydrogel® [13] with rPA after two immunizations. To achieve similar GMCs and GMTs using less Alhydrogel®, a third immunization is required [13]. Second, when rPA is formulated with at least 704 µg of Alhydrogel®, doses of rPA above 50 µg unexpectedly show a slight decrease in immunogenicity (Figure 5 and [14]) instead of an increase.

The cellular response to anthrax toxins has rarely been studied, most likely because the role that anti-PA antibodies play in protection has been so well documented [7]. However, the cellular response is critical for the support of antibody production. Studies of cell-mediated immune responses in individuals exposed to anthrax spores from bio-terrorism attacks in the Capitol building in 2001 demonstrated that the CD4+ immune response is potently stimulated in a dose-dependent manner by anthrax spores [25]. The magnitude of cell-mediated responses elicited from rPA-based vaccines in humans has yet to be reported. Here we have seen that the groups of participants who received the higher doses of adjuvanted rPA had fewer responses than those who received the lower doses of adjuvanted rPA (Figure 7). This trend does not correlate with the ELISA or TNA data, where the higher doses of rPA resulted in higher humoral responses. Additionally, results with the higher doses of the non-adjuvanted rPA are somewhat conflicting. The 50 µg dose has fewer responders, appearing to follow the trend observed in the higher doses of the Alhydrogel®-formulated rPA. The 100 µg PBS-formulated dose, however, showed only one non-responder, similar to the lower doses (both formulations). The assessment of T cell stimulation through LPA provides indirect evidence of T cell help induced by the vaccine regimen. However, the observed T cell response did not directly correlate with the humoral response. Additionally, these data conflict with the data generated from spore exposure in humans [25]. It is probable that spores activate the T-helper cell response more potently than does rPA alone. One caveat is that the sample size for each group is quite small (n = 5–8, depending on the group), thus the current trends may be different than trends that would be observed in larger datasets. Further experiments are required to explore this issue.

The *E. coli* derived rPA product evaluated for the first time in humans in this study was safe, well-tolerated and produced humoral immune responses comparable to those observed in previous human trials of other recombinant PA vaccines. Based on the safety and immunogenicity data described here, 50 µg of rPA formulated with Alhydrogel® would provide the best immune response while minimizing the amount of antigen administered. Further study of this product with a late boost or novel adjuvants may enhance durability and peak immune response.

## Supporting Information

Checklist S1 CONSORT Checklist
(0.22 MB DOC)

Protocol S1 Trial Protocol
(10.72 MB PDF)
