# Supplementary material for: Phase I Study of Safety and Immunogenicity of an Escherichia coli-Derived Recombinant Protective Antigen (rPA) Vaccine to Prevent Anthrax in Adults
Source: PLoS One. 2010 Nov 5;5(11):e13849. doi: 10.1371/journal.pone.0013849 (PMC2974626; doi:10.1371/journal.pone.0013849)
Supplement: Protocol S1 — Trial Protocol (10.72 MB PDF) [file pone.0013849.s002.pdf]

CLINICAL PROTOCOL

A Phase 1 Study of Safety and Immunogenicity of *E. coli*-Derived Recombinant Protective Antigen (rPA), a New Anthrax Vaccine Administered by the Intramuscular (IM) Route in Healthy Adults

IND Number: 10588 / Protocol Numbers: rPA-EC-02 / RV147
VERSION 1.9, December 14, 2004

Principal Investigator:		Merlin Robb, MD<br>Director, HMJF HIV Program
Sponsor (IND Holder):		DynPort Vaccine Company LLC (DVC)<br>64 Thomas Johnson Drive<br>Frederick, MD 21702<br>POC: Gordon Wynant<br>Phone: (301) 607-5239<br>Fax: (301) 607-5099
Manufacturers:	**rPA**	**Production:**<br>Cambrex BioScience Inc.<br>Hopkinton, MA 01748<br><br>**Pilot Bioproduction Unit:**<br>Kenneth Eckels<br>Walter Reed Army Institute of Research (WRAIR)<br>Brookville Road<br>Forest Glen Annex, Building 501<br>Silver Spring, MD 20910
Manufacturers**:**	**Alhydrogel**	HCI Biosector<br>Elsenbakken 23<br>Frederikssund DK-3600<br>Distributor: E.M. Sergent, Clifton, NJ.
Clinical Site:		WRAIR / Henry M. Jackson Foundation (HMJF)<br>Vaccine Clinical Research Center<br>1600 East Gude Dr., Rockville, MD 20850
Clinical Study Coordinator:		Kathleen Duffy, R.N. (HMJF)
Local Medical Monitor:		CDR Sybil Tasker (NMRC)<br>Department of Infectious Disease<br>National Naval Medical Center<br>Bethesda, MD<br>301-295-4237 ext 8<br>cell phone 202-361-3923
Sponsor Medical Monitors:		Kelly McKee, Jr., MD, MPH<br>DynPort Vaccine Company LLC (DVC)<br>(301) 607-5061<br>24 hr phone: (301) 633-0056

The information contained herein is the property of DVC and WRAIR and may not be reproduced published or disclosed to others without written authorization from both parties.

PROTOCOL SUMMARY

Title of study: A Phase 1 Study of Safety and Immunogenicity of *E. coli*-Derived Recombinant Protective Antigen (rPA), a New Anthrax Vaccine Administered by the Intramuscular (IM) Route in Healthy Adults.

Sponsor (IND Holder): DynPort Vaccine Company LLC (DVC)

Project Phase: Phase 1

This study will provide preliminary safety and comparative immunogenicity data for the *E.coli*-derived rPA vaccine administered by intramuscular (IM) injection at Day 0 and Month 1.

Doses will range from 5 _g to 100 _g rPA, and at each dose-level, rPA will either be combined with phosphate-buffered saline (PBS) or adsorbed to Alhydrogel.

This study is exploratory and seeks to establish sufficient data to subsequently design further clinical studies.

Study Design:
This is a safety study with an open-label part (2 groups), followed by a dose-ranging part evaluating safety and immunogenicity using a double-blind, sequential-group design with randomization and placebo-control within each of the 6 groups. Volunteers in each dose group will receive two IM injections at Day 0 and Month 1 as outlined below:

Open-label, non-controlled part:
for each treatment group listed below, 5 study volunteers will receive doses as specified:

Group I 5ug rPA with PBS
Group II 5ug rPA with Alhydrogel

Double-blind, randomized, placebo-controlled part: for each treatment group listed below, 2 study volunteers will receive placebo (*PBS*) and 8 study volunteers will receive doses as specified:

Group III: 25 µg rPA with PBS
Group IV: 25 µg rPA with Alhydrogel
Group V: 50 µg rPA with PBS
Group VI: 50 µg rPA with Alhydrogel
Group VII: 100µg rPA with PBS
Group VIII : 100 µg rPA with Alhydrogel

Study volunteers:
70 individuals in good health, 18 to 40 years of age, and available for at least 12 months of follow-up from the time of first injection. Five (5) study volunteers will be enrolled in each of the two open label groups (Groups I - II) and 10 study volunteers in each subsequent group, 8 receiving active agent and 2 receiving a placebo.

Study Site: Walter Reed Army Institute of Research / Henry M. Jackson Foundation Vaccine Clinical Research Center, 1600 East Gude Dr., Rockville, MD 20850

Clinical Study Coordinator: Kathleen Duffy, R.N. (HMJF)

**Principal Investigator:**	Dr. Merlin Robb (HMJF)
**Co-Investigators:**	Dr. Mary Marovich (WRAIR)<br>Dr. Shirley Lee-Lecher (WRAIR)<br>COL Deborah Birx (WRAIR)<br>Dr. Darrell Eugene Singer (WRAIR)<br>Dr. Jose Sanchez (WRAIR)
**Local Medical Monitor**:	CDR Sybil Tasker (NMRC)
**Sponsor Medical Monitor:**	Dr. Kelly McKee (DVC)
**Laboratory Investigators**	Dr. Thomas Van Cott (HMJF)<br>Dr. Josephine Cox (HMJF)<br>Dr. Victoria Polonis (HMJF)
**Coordinating Data Center**:	Data Coordinating and Analysis Center<br>U.S. Military HIV Research Program<br>1600 East Gude Dr, Rockville, MD 20850

Product Description: Recombinant PA (rPA) derived from a commercially available, non-pathogenic, laboratory strain of *Bacillus anthracis* was produced in *Escherichia coli*. rPA is expressed as a single polypeptide chain of 767 amino acids. It was produced in 12 liter scale by Cambrex BioScience Inc. (formerly Marathon Biopharmaceuticals Inc.), Hopkinton, MA, and purified to >90% purity by 4 sequential column chromatographic steps. The bulk rPA was formulated (25 mM Sodium Phosphate, 150 mM NaCl, pH 8.0) and vialed at two separate concentrations. In addition, diluent was vialed and blank vials were processed by the WRAIR Pilot BioProduction Facility (Forest Glen, MD).

Alhydrogel (aluminum hydroxide) is a commonly used adjuvant licensed for use in human vaccines.

Placebo recipients will receive PBS in a similar volume and by similar route.

Route of Administration: Intramuscular (IM, 1 mL) injection into deltoid muscle.

IMMUNIZATION SCHEDULE

Group No.	Study Group	No. Volunteers	No. Placebo Volunteers
Group I	5 µg rPA with PBS	5	0
Group II	5 µg rPA with Alhydrogel	5	0
Group III	25 µg rPA with PBS	8	2
Group IV	25 µg rPA with Alhydrogel	8	2
Group V	50 µg rPA with PBS	8	2
Group VI	50 µg rPA with Alhydrogel	8	2
Group VII	100 µg rPA with PBS	8	2
Group VIII	100 µg rPA with Alhydrogel	8	2
TOTAL		58	12

STUDY ENDPOINTS

Safety and Tolerability (Adverse events and laboratory abnormalities): Study volunteers will be observed for 30 minutes following immunization for evidence of immediate local and systemic reactions. Study Volunteers will be instructed to maintain a diary for local and systemic reactions for 7 days post-immunization.

The study nurse or designee will contact all volunteers by telephone on the day following each vaccination to determine if any reactions have occurred. If significant reactions are reported by the volunteer following vaccination, the volunteer will be instructed to return to the clinic for evaluation including safety labs.

At 48-72 hours after immunization, the volunteers will return to the clinic for an interval evaluation and laboratory measurements of hematology, serum chemistry, and urinalysis to assess safety.

Two weeks after each injection, all volunteers will undergo a directed medical evaluation with a physician to review the diary card, interval history, physical exam and laboratory measurements of hematology, serum chemistry, and urinalysis to assess safety. Volunteers will be encouraged to report any adverse events at any time and may be asked to come into the clinic for evaluation by the research medical team. All adverse events will be recorded and their relationship to test product assigned by the Principal Investigator (PI) and reviewed by the Local Medical Monitor.

All adverse events will be tabulated by relationship to study product, organ system, severity and temporal relation to vaccination.

Immunogenicity Assays: Blood will be drawn for use in immunogenicity assays (see Study Flow Chart). Assays that will be used to evaluate the humoral immune response to rPA include the serum anti-rPA immunoglobulin G (IgG) enzyme-linked immunosorbent assay (ELISA), the

serum toxin neutralizing antibody (TNA) assay and the rPA-specific B cell antibody secreting cell assay (ASC).

Blood Requirement: Total blood volume: approximately 518 mL over 13 months.

Interim and Final Analysis: Preliminary safety and tolerability analysis will be performed after the initial immunization of all groups. Preliminary immunogenicity analyses will be performed after the second immunization. A final analysis will be performed after all volunteers have completed their final visits.

STUDY FLOWCHART

**Study visit (SV)**<br>**Telephone Contact (TC)**	1	2	3	TC	4	5	6	TC	7	8	9	10	11	12	13
Day	- 1<br>Pre-Screening	-2<br>Final Screen	0	1	2-3	14	28 +/- 7	29	32-34	42	70	112	182	252	364
Week	14 - 60 days<br>pre vaccination	3 -45 days<br>pre vaccination	0	24 hrs	48-72 hrs	2	4	24 hrs	48-72<br>hrs	6	10	16	26	36	52
Month			0	.1	0.25	0.5	1	1.1	1.25	1.5	2.5	4	6.5	9	13
**Administrative Requirement**															
Registration, Informed Consent and Test of Understanding	X														
Volunteer registry database sheet	X														X
**Immunization and Safety Monitoring**			**X**				**X**								
**Clinical Requirements**															
Telephone Contact				X				X							
Complete History & Physical	X														
Interim History &Physical and Assessment of AEs		X	X		X	X	X		X	X	X	X	X	X	X
Review and/or Collect Diary Card				X	X	X		X	X	X					
**Safety Labs:**															
HIV serology (4 mL clot tube)	4 mL														
Hepatitis serology (3 mL SST)	4 mL														
Troponin I and II (3 mL Heparin tube)			3 mL			3 mL				3 mL					
Safety Labs and UA *<br>(3 mL EDTA, 4 mL clot)	7 mL	7 mL#	7 mL		7 mL	7 mL	7 mL		7 mL	7 mL	7 mL	7 mL	7 mL	7 mL	7 mL
Serum Pregnancy Test +(no additional)	X		X				X								X
**Immunogenicity Labs**															
Anti-PA IgG ELISA/TNA (clot tubes)		10 mL	10 mL			10 mL	10 mL			10 mL	10 mL	10 mL	10 mL	10 mL	10 mL
CMI assays** (heparinized tubes)		30 mL	30 mL			30 mL				30 mL	30 mL	30 mL	30 mL	30 mL	30 mL
Sample for production of human monoclonal anti-PA antibodies										40 mL					
TOTAL Blood = 518 mL over 13 months	15 mL	47 mL	50 mL		7 mL	50 mL	17 mL		7 mL	90 mL	47 mL	47 mL	47 mL	47 mL	47 mL

+ Test results must be known within 24 hrs of vaccination
* Safety Labs include: (4 mL red top) electrolytes, creatinine, CPK, AST, ALT, GGT, Ca, Phosphate, BUN, uric acid, total bilirubin, (3 mL EDTA) CBC w/differential, and urine dipstick analysis.
# Safety Labs (blood draw) and UA done if necessary.
** Assays for cell-mediated immune response (CMI) under development: LPA (lymphocyte Proliferation Assay), _-IFN ELISPOT (ELISA-based assay for quantifying gamma-interferon producing lymphocytes), ICC (Intracellular Cytokine Assay)

TABLE OF CONTENTS **Page**

1.0 INVESTIGATORS AND STUDY ADMINISTRATIVE STRUCTURE 13
2.0 ETHICS 13
2.1 Institutional Review Board (IRB) 13
2.2 Informed Consent 14
2.3 Volunteer Registry Database 14
3.0 BACKGROUND AND RATIONALE 14
3.1 Biology of Bacillus Anthracis 15
3.2 Vaccination Against Anthrax 15
3.3 Protective Immunity Against Anthrax 16
3.4 Non Clinical Studies conducted with B. anthracis derived rPA Vaccine 17
3.4.1 Efficacy Studies with B. anthracis-derived rPA in Rabbits 17
3.4.1.1 Single-dose Studies in Rabbits 17
3.4.1.2 Dose-ranging Study in Rabbits (two injections) 20
3.4.2 Efficacy Studies with B. anthracis-derived rPA in Nonhuman Primates 21
3.4.2.1 Single-dose Study in Nonhuman Primates 21
3.4.2.2 Dose-ranging Study in Nonhuman Primates 23
3.5 History of rPA Expression in E. coli and the Involvement of WRAIR in the Development of a New Anthrax Vaccine 24
3.6 Non Clinical Studies conducted with E. coli- derived rPA Vaccine 25
3.6.1 Study in Rabbits Comparing E. coli-derived rPA / Alhydrogel to B. anthracis-derived rPA / Alhydrogel 25
3.6.2 Studies in Mice Comparing E. coli-derived rPA to B. anthracis -derived rPA and Comparing to Alhydrogel 25
3.7 ALHYDROGEL: 27
3.7.1 Alhydrogel 27
4.0 STUDY OBJECTIVES 28
4.1 Primary Study Objectives: 28
4.2 Primary Study Endpoints: 28
4.3 Immunologic Studies: 28
5.0 INVESTIGATIONAL PLAN 28
5.1 Overall Study Design and Plan 28
5.1.1 Recruiting of Anthrax Vaccine Naïve Volunteers 28
5.1.2 Screening and Informed Consent Procedures 29
5.1.3 Assignment to Treatment Group 29
5.1.4 Recording of Acute Adverse Experiences and Maintenance of Study volunteer's Diary 29
5.1.5 Criteria for Suspension of Vaccine Administration (“Stopping Rules”) 30

5.1.5.1 Suspension of Vaccine Administration within a Treatment Group 30
5.1.5.2 Suspension of ALL Vaccine Administrations 31
5.1.5.3 Premature Termination of the Clinical Trial 31
5.1.6 Dose-Escalation and Progression to the Next Treatment Group (Dose-Level) 31
5.1.6.1 Open label phase (Groups I –II) 32
5.1.6.2 Randomized Double Blind Phase (Groups III-VIII) 32

5.2 Rationale for the Immunization Schedule and Study Groups 33

5.3 Assessment of Immunogenicity 33
5.3.1 Primary Measures of Immunogenicity 33

5.4 Selection of Study Populations 34
5.4.1 Inclusion Criteria 34
5.4.2 Exclusion Criteria 35
5.4.3 Withdrawal of Study volunteers from Trial and/or Assessment 35
5.4.4 Replacement of Study volunteers 36

5.5 Administration of Vaccine and Placebo 37
5.5.1 Summary of Test Articles 37
5.5.1.1 Recombinant Protective Antigen 37
5.5.1.2 Alhydrogel 37
5.5.2 Formulation of rPA Anthrax Vaccine 37
5.5.2.1 Procedure for Reconstitution 38
5.5.2.2 Dosage, Administration, and Labeling 38
5.5.2.3 Disposition 39
5.5.2.4 Precautions to be Observed in Administering Study Vaccine 39
5.5.3 Method of Assigning Study volunteers to Study Groups 40
5.5.4 Controls/Blinding 40
5.5.5 Prior and Concomitant Medications/Vaccines 40
5.5.6 Study Procedures 43
5.5.6.1 Enrollment Visits 1 and 2. 43
5.5.6.2 Visit 3, Day 0: First Immunization: 43
5.5.6.3 Telephone Contact after the First Immunization (TC): 44
5.5.6.4 Visit 4, 48-72 Hour Safety Evaluation after the First Immunization: 44
5.5.6.5 Visit 5, Week 2 (window ± 7 days) Safety and Immunogenicity: 44
5.5.6.6 Visit 6, Week 4 (window ± 7 days) Second Immunization: 44
5.5.6.7 Telephone Contact after the Second Immunization (TC) 45
5.5.6.8 Visit 7, 48-72 Hour Safety Evaluation after the Second Immunization: 45
5.5.6.9 Visit 8, Week 6 (± 7 days) Safety and Immunogenicity Visit: 45
5.5.6.10 Visit 9, Week 10 (± 7 days) Safety and Immunogenicity Visit: 45
5.5.6.11 Visit 10, Week 16 (± 7 days) Safety and Immunogenicity Visit: 46
5.5.6.12 Visit 11, Week 26 (± 2 weeks) Clinical and Immunological Follow-up: 46
5.5.6.13 Visits 12, Week 36 (± 2 weeks): 46
5.5.6.14 Visit 13, Week 52 (± 2 weeks) Clinical and Immunological Follow-up: 46

5.6 Study Variables and Their Measurement 47
5.6.1 Immunogenicity Variables 47
5.6.1.1 Humoral Immune Responses to rPA: 47
5.6.1.2 Cellular Immune Responses to rPA: 47
5.6.1.3 Ancillary Studies: 49
5.6.2 Specimen Handling and Processing: 49

5.6.2.1 Sample collection 50
5.6.3 Safety Variables 50
5.6.3.1 Adverse Events: 50
5.6.3.2 Reporting of Adverse Events 52
5.6.3.3 Monitoring of Post-Immunization Events: 53
5.6.3.4 Post-immunization Reactions Occurring Immediately After Immunization 53
5.6.3.5 Telephone Contact after each Immunization (TC) 54
5.6.3.6 Safety Evaluation after each Immunization 54
5.6.3.7 Local Reactions Within 7 Days Post-immunization (Study Days 0 to 6) 54
5.6.3.8 Systemic Reactions Within 7 Days Post-immunization (Study Days 0 to 6) 54
5.6.3.9 Post-immunization Contact 54
5.6.3.10 Instructions to Study Volunteers Regarding Unusual or Severe Signs or Symptoms 55

5.7 Statistical Methods and Determination of Sample Size 55
5.7.1 Statistical Plan: Design & Analysis 55
5.7.2 Safety 56
5.7.2.1 Post-immunization Reactions (Local and Systemic) 56
5.7.2.2 Other Adverse Events 56
5.7.2.3 Clinical Laboratory 56
5.7.3 Immunogenicity 56

6.0 COMPENSATION, INSURANCE, INDEMNITY 57

6.1 Information on Compensation, Insurance and Indemnity 57

7.0 VACCINE ACCOUNTABILITY 57

8.0 LABORATORY REQUIREMENTS 57

9.0 CASE REPORT FORMS 57

10.0 STUDY MONITORING AND COMPLIANCE 58

11.0 INFORMED CONSENT 58

12.0 RETENTION OF RECORDS 59

13.0 USE OF INFORMATION AND PUBLICATION 59

14.0 PROTOCOL AMENDMENTS 59

15.0 REFERENCES 61

16.0 SIGNATURE PAGE 64

17.0 APPENDICES 66

17.1 STATEMENT OF OBLIGATIONS OF SPONSOR/MONITOR, MEDICAL MONITOR, AND CLINICAL INVESTIGATOR 66

17.2 BRIEFING DOCUMENT FOR STUDY VOLUNTEER'S ENROLLMENT PROCEDURE 69

17.3 DRAFT INFORMED CONSENT FORM 70

17.4 TEST OF UNDERSTANDING AND COMMITMENT 71

17.5 PREGNANCY REPORTING 72

17.6 MEDICAL MONITORS 73

17.7 TOXICITY GRADING SCALE ____ 74
17.8 VACCINE PREPARATION ____ 81
17.9 LABEL DESCRIPTION OF INVESTIGATIONAL PRODUCT ____ 82
18.0 VOLUNTEER REGISTRY DATA SHEET (USAMRDC 60-R) ____ 82

LIST OF FIGURES

FIGURE 1. AVERAGE WEEK 4 GEOMETRIC MEAN ELISA TITERS. ____ 19
FIGURE 2. ANTI-PA IGG TITERS AND CHALLENGE-SURVIVAL IN RABBITS AFTER RPA VACCINE SINGLE DOSES (SOLID DIAMONDS = SURVIVAL, OPEN DIAMONDS = DEATH) ____ 20
FIGURE 3. ANTI-PA IGG TITERS IN THE RABBITS AFTER ADMINISTRATION OF TWO DOSES OF RPA OR AVA ____ 21
FIGURE 4. ANTI-PA IGG TO AVA AND RPA IN THE NONHUMAN PRIMATE ____ 22
FIGURE 5. ANTI-PA IGG RESPONSE TO AVA AND RPA IN THE NONHUMANPRIMATE____23
FIGURE 6. PRODUCTION OF ANTI-PA IGG IN BALB/C, H-2D MICE FOLLOWING TWO DOSES (0 AND 28 DAYS) OF VACCINE CONTAINING 4 μG OF RPA ALONE OR IN COMBINATION WITH ALHYDROGE ____26
FIGURE 7. PRODUCTION OF ANTI-PA IGG IN BALB/C, H-2D MICE FOLLOWING TWO DOSES (0 AND 28 DAYS) OF VACCINE CONTAINING 20 μG OF RPA ALONE OR IN COMBINATION WITH ALHYDROGEL.____26

LIST OF TABLES

TABLE 1. CHALLENGE-SURVIVAL AND ANTI-PA TITERS IN RABBITS AFTER RPA SINGLE DOSE. ____ 18
TABLE 2. CHALLENGE-SURVIVAL IN RABBITS AFTER TWO DOSES OF RPA OR AVA ____ 20
TABLE 3. CHALLENGE-SURVIVAL IN NONHUMAN PRIMATES AFTER RPA SINGLE DOSE ____ 21
TABLE 4. MEAN TOXIN NEUTRALIZATION ANTIBODY (TNA) TITERS IN RHESUS MACAQUE, 5 WEEKS AFTER VACCINATION ____ 23
TABLE 5. CHALLENGE-SURVIVAL IN NONHUMAN PRIMATES AFTER TWO DOSES OF RPA ____ 23
TABLE 6. RPA IMMUNOGENICITY STUDY ____ 26
TABLE 7 SUMMARY OF TREATMENT GROUPS FOR THE DOSE-ESCALATION OF RPA (WITH OR WITHOUT ADJUVANT) ____ 31
TABLE 8 FORMULATION OF THE RPA ANTHRAX VACCINE ____ 38
TABLE 9 STUDY FLOW CHART ____ 41
TABLE 10 TOXICITY GRADING SCALE FOR ADVERSE EVENTS ____ 74

LIST OF ABBREVIATIONS

Ab	Antibody
ACD	Acid Citrate Dextrose
ADL	Activity of Daily Living
AE	Adverse Event
AL	Alhydrogel
ALT	Alanine Amino Transferase
ASC	Antibody Secreting Cell
AST	Aspartate Amino Transferase
AVA	Anthrax Vaccine Adsorbed (BioThrax™)
BSL-II	Biosafety Level 2
CBC	Complete Blood Count
CBER	Center for Biologics Evaluation and Research (FDA)
CDC	Centers for Disease Control
CDR	Commander
CFR	Code of Federal Regulations
CFU	Colony Forming Units
CGMP	Current Good Manufacturing Practices
CIOMS	Council of International Organization of Medical Sciences
cm	Centimeter
CMI	Cell-mediated Immune Response
COL	Colonel
CPK	Creatinine Phosphokinase
CPM	Counts Per Minute
CPMP	Committee for Proprietary Medicinal Products
CRF	Case Report Form
CTL	Cytotoxic T Lymphocyte
DCAC	Data Coordinating and Analysis Center
DEERS	Defense Eligibility and Enrollment Reporting System
dL	Deciliter
DNA	Deoxyribonucleic Acid
DVC	DynPort Vaccine Company
EDTA	Ethylene-diamine-tetra-acetic Acid
EF	Edema Factor of *Bacillus anthracis*
ELISA	Enzyme-Linked ImmunoSorbent Assay
ELISPOT	Enzyme-Linked ImmunoSorbent Spot Assay
FDA	U.S. Food and Drug Administration
GMT	Geometric Mean Titer
HBsAg	Hepatitis B Surface Antigen
HIV	Human Immunodeficiency Virus
HMJF	Henry M. Jackson Foundation for the Advancement of Military Medicine
HRP	Horseradish Peroxidase
HURC	Human Use Review Committee (WRAIR IRB)
HSRRB	Human Study Volunteers Research Review Board (MRMC IRB)
IB	Investigator's Brochure
ICC	IntraCellular Cytokine Assay
ICH	International Conference on Harmonization Regulations
_-IFN	Gamma Interferon
IgG, IgA	Immunoglobulin G, Immunoglobulin A
IL	Interleukin

IM	Intramuscular
IRB	Institutional Review Board
IV	Intravenous
LD_{50}	Lethal Dose (50%)
LF	Lethal Factor of *Bacillus anthracis*
LPA	Lymphocyte Proliferation Assay
LSI	Lymphocyte Stimulation Index
MC	Medical Corps
MedDRA	Medical Directory for Regulatory Activities
mg	Milligram
MHC	Major Histocompatibility Complex
mL	Milliliter
mM	Millimolar
MSD	Merck Sharp and Dohme
MTT	3-[4,5-dimethylthiazol-2-yl]-2,5-diphenyl-tetrazolium bromide
MW	Molecular Weight
NIH	National Institutes of Health
NMRC	Naval Medical Research Center
NZW	New Zealand White rabbit
PBS	Phosphate Buffered Saline
PI	Principal Investigator
PA	Protective Antigen of *Bacillus anthracis*
PBMC	Peripheral Blood Mononuclear Cells
PIN	Personal Identification Number
RBC	Red Blood Cell Count
RN	Registered Nurse
rPA	Recombinant Protective Antigen
RP-HPLC	Reverse Phase High Performance Liquid Chromatography
SAE	Serious Adverse Event
SST	Serum Separator Tube
TBD	To Be Determined
Th-1	Type 1 Helper T Cells
Th-2	Type 2 Helper T Cells
TNA	Toxin Neutralizing Antibody
TNF	Tumor Necrosis Factor
UA	Urinalysis
μg	Microgram
USAMRIID	US Army Medical Research Institute of Infectious Diseases
USC	United States Code
VAERS	Vaccine Adverse Event Reporting System
WBC	White Blood Cell Count
WRAIR	Walter Reed Army Institute of Research

1.0 INVESTIGATORS AND STUDY ADMINISTRATIVE STRUCTURE

**Principal Investigator:**	Dr. Merlin L. Robb (WRAIR)
**Clinical Investigators:**	Dr. Mary Marovich (WRAIR)<br>Dr. Shirley Lee-Lecher (WRAIR)<br>COL Deborah Birx (WRAIR)<br>Dr. Darrell Eugene Singer (WRAIR)<br>Dr. Jose Sanchez (WRAIR)
**Sponsor Medical Monitor**:	Dr. Kelly McKee<br>DynPort Vaccine Company LLC (DVC)
**Local Medical Monitor:**	CDR Sybil Tasker, MD, FACP<br>Department of Infectious Disease<br>National Naval Medical Center<br>Bethesda, MD
**Laboratory Investigators:**	Dr. Thomas VanCott<br>Dr. Josephine Cox<br>Dr. Victoria Polonis<br>WRAIR / HMJF<br>1600 East Gude Dr., Rockville, MD 20850
**Clinical Study Coordinator:**	Kathleen Duffy, R.N. (HMJF)<br>1600 East Gude Dr., Rockville MD 20850<br>Tel : 301-251-8350 / Fax: 301-424-3120
**Coordinating Data Center**:	Data Coordinating and Analysis Center<br>U.S. Military HIV Research Program<br>1600 East Gude Dr., Rockville, MD 20850

2.0 ETHICS

2.1 Institutional Review Board (IRB)

The principal investigator agrees to provide the IRB with all appropriate material, including the informed consent documents. This trial will not be initiated until appropriate IRB approval of the protocol, informed consent document, and all recruiting materials has been obtained in writing by the investigator and copies have been received by the sponsor. Appropriate reports on the progress of the study by the principal investigator will be made to the IRB and the sponsor in

accordance with applicable government regulations and in agreement with policy established by the sponsor.

2.2 Informed Consent

Written informed consent, in compliance with the Belmont Report, the guidelines of the Council of International Organization of Medical Sciences (CIOMS) and U.S. Code of Federal Regulations 21 CFR 50, shall be obtained from each study volunteer's prior to enrollment into the trial or prior to performing any unusual or non-routine procedure that involves risk to the study volunteer's.

The investigator shall provide a copy of the IRB-approved informed consent to the study volunteer and a signed copy shall be maintained in the study volunteer's record file. Attention is directed to the basic elements that are required to be incorporated into the informed consent under U.S. Federal Regulations for Protection of Human Study volunteers (21 CFR 50.25[a]). Additional elements of informed consent, if appropriate, must be included in the informed consent document (21 CFR 50.25[b]).

2.3 Volunteer Registry Database

It is the policy of USAMRMC that data sheets are to be completed on all volunteers participating in research for entry into the U.S. Army Medical Research and Materiel Command Volunteer Registry Database. The information to be entered into this confidential database includes name, address, social security number, study name, and dates. The intent of the database is twofold: first, to readily answer questions concerning an individual's participation in research sponsored by the USAMRMC; and second, to ensure that the USAMRMC can exercise its obligation to ensure research volunteers are adequately warned (duty to warn) of risks and to provide new information as it becomes available. The information will be stored at the USAMRMC for a minimum of 75 years. The investigator and the study volunteer must complete USAMRDC Form 60-R. (appendix 18). All completed form must be sent to the following address:

Commanding General, U.S. Army Medical Research and Materiel Command
ATTN: MCMR-RCQ-HR
504 Scott Street
Fort Detrick, Maryland 21702-5012

3.0 BACKGROUND AND RATIONALE

Bacillus anthracis (*B. anthracis*) is a human and animal pathogen with variable manifestations and prognosis depending on route of acquisition. Entry of spores through a break in the skin, ingestion or inhalation produce cutaneous, gastrointestinal and inhalation anthrax, respectively (1). Anthrax disease is normally a disease of animals, particularly herbivores, and produces sporadic or epidemic disease in humans who handle products of infected animals or ingest contaminated meat (2). Disease develops when spores of *B. anthracis* germinate and the vegetative bacilli express the principal virulence factors consisting of two exotoxins and an antiphagocytic capsule. Cutaneous anthrax is rarely fatal when treated with antibiotics and

gastrointestinal anthrax is associated with an intermediate prognosis (3). However, inhalation anthrax is usually fatal.

3.1 Biology of *Bacillus Anthracis*

The organism is a gram positive, non-motile spore-forming bacillus, which exists ubiquitously in the soil (4,5). The size and durability of the spore across a broad range of environmental conditions and the high mortality of inhalation anthrax from the easily aerosolized spores account for development of *B. anthracis* by several nations as a biological weapon (6). Widespread concerns that *B. anthracis* spores could be used in terrorist attacks have recently been realized in New York City, Florida and Washington, D.C. (7). Approaches to prevention of anthrax disease after such exposures include antibiotics, passive and active immunization and, theoretically, anti-toxin therapy (3). In animals exposed to lethal aerosols of anthrax spores, death may occur when effective chemoprophylaxis is withdrawn. The administration of the currently licensed vaccine, BioThrax™ , Anthrax Vaccine Adsorbed (AVA), affords durable protection against inhalation exposure in Rhesus macaques when co-administered with antibiotics (8). However, the short incubation period between exposure and disease precludes the use of vaccine alone to prevent inhalation anthrax under most circumstances (8,9). Availability and use of a safe and effective vaccine would discourage use of *B. anthracis* as a weapon by terrorists or rogue states.

Avirulent strains of *B. anthracis* exist in nature and have been developed as live attenuated vaccines for domesticated animals since 1937. The pathogenesis of anthrax disease is related to the presence of a capsule and expression of two exotoxins (10,11). Failure to express a capsule results in a 100,000-fold attenuation of anthrax in animals and absence of exotoxin results in a completely avirulent phenotype (12,13). Each toxin is formed when either Lethal Factor (LF) or Edema Factor (EF) binds to Protective Antigen (PA). The three toxin components (LF, EF and PA), which are encoded on plasmid pX01, are non-virulent alone. PA binds to the host cell receptors and following proteolytic cleavage, forms PA heptamers which bind either EF or LF at high affinity forming edema toxin and lethal toxin respectively. This bipartite complex constitutes the active toxin. The complex is transferred into the cell via endocytosis, and under conditions of low pH, the PA forms a β-barrel followed by translocation of the toxic enzyme into the cytosol (14,15).

Edema toxin is a calmodulin dependant adenylate cyclase and produces local edema and impairs neutrophil function *in vitro* and *in vivo* (16). Lethal toxin is a metalloprotease that inactivates mitogen-activated protein kinase-kinase (17,18). Lethal toxin induces overproduction of numerous lymphokines and expression results in macrophage lysis and release of large amounts of TNF-α and IL-1β, which contribute to the rapid lethality of systemic anthrax (18). The capsule inhibits phagocytosis and, together with the exotoxins, they alter and degrade host immune response to the vegetative anthrax bacilli and are responsible for the clinical manifestations and mortality of anthrax disease (3,19).

3.2 Vaccination Against Anthrax

The first anthrax vaccines were developed for animal use and consist of live attenuated strains of *B. anthracis* which lack either the plasmid encoding the capsule protein (e.g. Pasteur strain) or

the plasmid expressing the tripartite exotoxins of anthrax (e.g. Sterne strain). These attenuated strains have been successful in the control of anthrax among livestock. They are also used to generate the licensed vaccine products in the United States and the United Kingdom which are cell-free culture-filtrates of attenuated *B. anthracis* strains (20).

The only data supporting human efficacy of anthrax vaccines were generated in a placebo-controlled field trial of such a cell-free culture-filtrate vaccine produced by Merck Sharp and Dohme (the MSD vaccine) in mill workers handling imported, anthrax-contaminated goat hair (21). This study demonstrated a 92.5% protection against cutaneous anthrax (95% lower-bound confidence interval of 65%). However, this study did not have sufficient statistical power to assess protection against inhalation anthrax, but was shown to prevent inhalation anthrax in Rhesus macaques following aerosol challenge (22).

The licensed U.S. vaccine, Anthrax Vaccine Adsorbed (BioThrax™) is a cell-free culture-filtrate of an avirulent, nonencapsulated *B. anthracis* strain (V770-NP1-R) grown in a microaerophilic environment. It predominantly consists of PA and is adsorbed to aluminum hydroxide. In addition to PA, small amounts of LF and EF are found in some lots of AVA. AVA also contains 0.02% formaldehyde (stabilizer) and 0.0025% benzethonium chloride (preservative), and is stored at 2-8°C. AVA is administered subcutaneously at a dose of 0.5 mL at 0, 2, and 4 weeks, followed by boosters at 6, 12, 18 months and annually thereafter (23). Human studies with AVA identify the presence of PA antibody in 83% of study volunteers after the primary series and 91% after the full six shot series (24,25).

In an open-label safety study, 15,907 doses of BioThrax™ were administered to approximately 7,000 textile employees, laboratory workers and other at risk individuals. There were 24 reports (0.15% doses administered) of severe local reactions and 1373 reports (8.63% of doses administered) of mild local reactions. Four cases of systemic reactions were reported during a five-year reporting period (<0.06% of doses administered). These reactions, which were reported to have been transient, included fever, chills, nausea and general body aches.

Over 2 million doses of BioThrax™ have been administered in the United States, and Vaccine Adverse Event Report System (VAERS) reports were received for approximately 1850 vaccinated persons.

The most frequently reported adverse events were erythema, headache, arthralgia, fatigue, fever, peripheral swelling, pruritus, nausea, injection site edema, pain/tenderness and dizziness. Approximately 6% of the reported events were listed as serious.

The AVA vaccine has indirect evidence of protective efficacy in humans but requires frequent primary and booster injections to establish and maintain immunity. A vaccine with durable immune responses and decreased reactogenicity, with more economical dosing is highly desirable.

3.3 Protective Immunity Against Anthrax

In animals, efficacy of anthrax vaccines is primarily assessed by survival from challenge with virulent *B. anthracis* spores of the Ames strain or the Vollum strain. In most cases, the animals

are exposed to aerosols of 50-150 times LD_{50} approximately 4-12 weeks after vaccination, and survival is monitored for 3-12 weeks after challenge.

As a potential correlate for efficacy, the extent of the humoral immune response is assessed by measuring the anti-PA serum IgG titer using the enzyme-linked immunosorbant assay (ELISA) or by measuring the serum antibody concentration which neutralizes toxin-mediated cytolysis in the toxin-neutralizing antibody (TNA) assay (Section 5.6.1.1). Immunogenicity at the level of cell-mediated immune response (CMI) can be assessed by measuring the specific antigen stimulated proliferation of memory T cells by the Lymphocyte Proliferation Assay (LPA), and the antigen-specific induced production of IFN-gamma by memory T cells using both the intracellular cytokine assay (ICC) and the number of cytokine producing memory T cells, e.g. gamma-interferon (_-IFN, after specific antigen stimulation by the _-IFN ELISPOT assay (Section 6.6.1.2).

In literature, it was shown in two different animal models, that the extent of humoral immune response after AVA vaccination correlates with survival following challenge with lethal doses of anthrax:

- In guinea pigs, TNA titers have been found to be an accurate correlate of protection against lethal intradermal challenge, based on results from three independent studies of active or passive immunization. TNA titers from 1:25 to 1:300 showed a positive linear correlation ($r^2 = 0.92$ and 0.95, respectively) with survival. There was 50% survival at a TNA titer above 80 and 100% survival in all animals with TNA titers above 300. Anti-PA IgG ELISA values did not correlate as well with survival ($r^2 = 0.56$) (26).
- In the rabbit model, after immunization with AVA at weeks 0 and 4, both anti-PA IgG measured by ELISA and TNA titers at weeks 6 and 10 were predictive of survival following inhalation challenge with a lethal dose of anthrax spores at week 10 (27).

3.4 Non Clinical Studies conducted with *B. anthracis* derived rPA Vaccine

Preliminary data in rabbits and monkeys with rPA derived from an avirulent, non-toxigenic, non-sporulating *B. anthracis* strain at USAMRIID, show correlation of anti-PA ELISA titer with survival following aerosol challenge at week 4 after single-dose vaccination, as described in the Investigator's Brochure (IB).

3.4.1 Efficacy Studies with *B. anthracis*-derived rPA in Rabbits

3.4.1.1 Single-dose Studies in Rabbits

To evaluate the use of the rabbit model for assessing correlates of immunity effected by the rPA anthrax vaccine, four separate experiments with New Zealand White (NZW) rabbits were performed. Those studies are summarized below (Table 1; Figures 1 and 2) to show correlation of anti-PA IgG titer measured by ELISA with survival following aerosol challenge at week 4 after vaccination.

The rabbits were inoculated with a single dose of various concentrations of rPA adsorbed to Alhydrogel. They were bled weekly for quantitative titers of anti-PA IgG measured by ELISA and TNA, and were challenged four weeks after rPA injection with an aerosol of Ames spores

(80 LD_{50}). Survival, summarized in Table 1, was recorded at 21 days after challenge. The serum has not yet been evaluated for neutralization of cytotoxicity with the TNA assay.

Table 1. Challenge-Survival and anti-PA Titers in Rabbits after rPA Single Dose.

	**EXPERIMENT #1**			**EXPERIMENT #2**			**EXPERIMENT #3**			**EXPERIMENT #4**			**SUMMARY**		
rPA (μg)	Survived/ Total	%	Titer§	Survived/ Total	%	Titer	Survived/ Total	%	Titer§	Survived/ Total	%	Titer	Survived/ Total	%	Titer
***B. anthracis* rPA**															
100	n/d			n/d			15/16	94	64.72	13/14	93	33.69	28/30	93.3	49.21
25	8/10	80	36.96	14/24	58	16.33	14/16	88	51.84	6/14	43	11.16	42/64	65.6	29.07
5	3/10	30	13.92	n/d			7/15	47	25.36	6/12	50	24.00	16/37	43.2	21.09
1	2/10	20	13.40	n/d			n/d			2/12	17	2.35	4/22	18.2	7.88
0.2	1/10	10	2.10	n/d			n/d			n/d			1/10	10.0	2.10
0.08	0/10	0	0.24	n/d			n/d			n/d			0/10	0	0.24

§, Week 4 average anti-PA IgG ELISA titer (μg anti PA IgG/mL) with HRP anti-rabbit IgG(H+L).
n/d = not done

A dose-dependent increase of geometric mean titers (GMT) in the anti-PA IgG ELISA was observed with the *B. anthracis* -derived rPA vaccine preparation as shown in Figure 1. Survival against aerosol challenge with Ames spores seems to correlate with the titers measured in the anti-PA IgG ELISA, as shown in Figure 2. Since the rPA concentrations evaluated did not result in a humoral immune response with an upper plateau, the highest possible anti-PA IgG ELISA titers would probably require an administration of an even higher dose of rPA than the 100 μg tested. Statistical analysis of the data by logistic regression analysis suggest that the odds of survival increase with increasing GMT. However, the GMT at week 4 were the strongest predictor of survival ($p<0.0001$) with a 10.7 fold increase in odds of survival per 1 log increase in GMT.

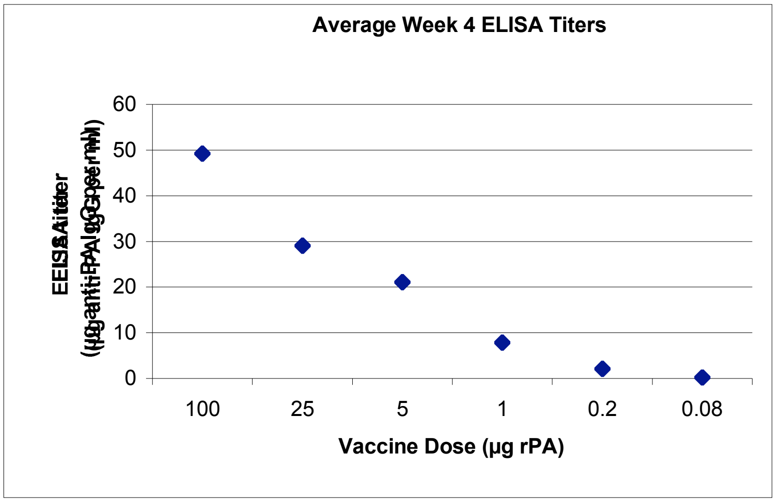

Figure 1. Average Week 4 Geometric Mean ELISA Titers.

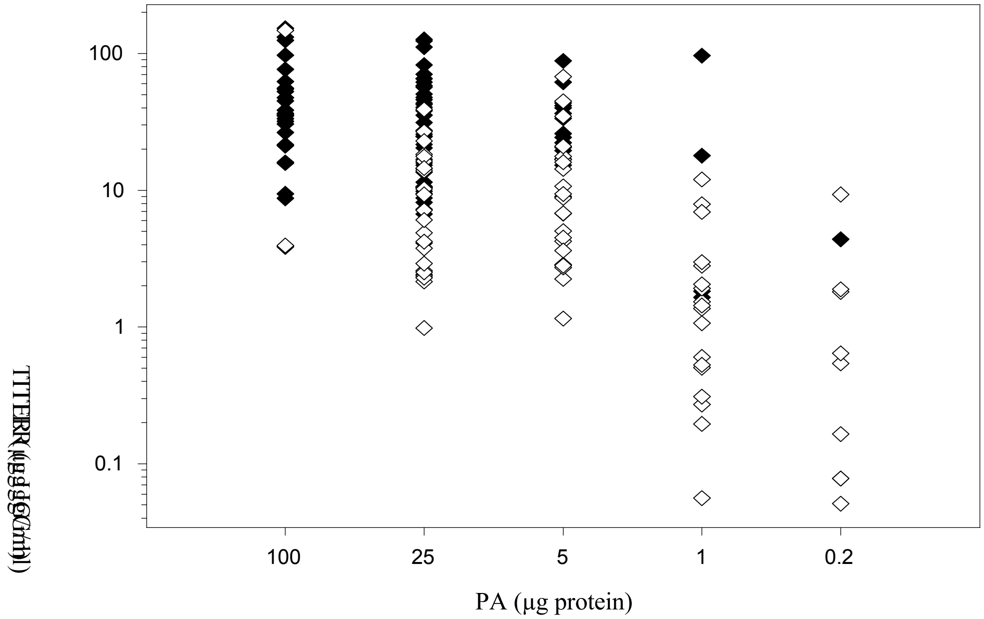

(solid diamonds = survival, open diamonds = death)

Figure 2. Anti-PA IgG Titers and Challenge-Survival in Rabbits after rPA Vaccine Single Doses

3.4.1.2 Dose-ranging Study in Rabbits (two injections)

A laboratory preparation of *B. anthracis*-derived rPA was tested using the rabbit animal model. This rPA was approximately 95% pure by RP-HPLC and was formulated in approximately 350 mM ammonium acetate, pH 10, 2 mM EDTA and 0.1 mM PMSF. Prior to injection, the rPA was formulated in Dulbecco's PBS (phosphate buffered saline) and adsorbed to Alhydrogel.

Ten NZW rabbits per group were vaccinated with 50, 5, or 0.5 _g of rPA adsorbed to Alhydrogel (725 _g aluminum/dose). The negative control group received Alhydrogel plus PBS, and the positive control group received a dose of AVA (Lot FAV018). Animals were immunized (IM) on days 0 and 28 and challenged by aerosol with a lethal dose (63 ± 2.9 LD_{50}) of *B. anthracis* spores of the Ames strain 3 months later. Survival data are summarized in Table 2.

Table 2. Challenge-Survival in Rabbits After Two Doses of rPA or AVA

Group	Vaccine	Survived Challenge
1	AVA	9/10
2	50 µg rPA/AL	10/10
3	5 µg rPA/AL	9/10
4	0.5 µg rPA/AL	6/10
5	AL/PBS	0/10

The rabbits were bled every 2 weeks and the GMT, measured in the anti-PA IgG ELISA, are presented in Figure 3.

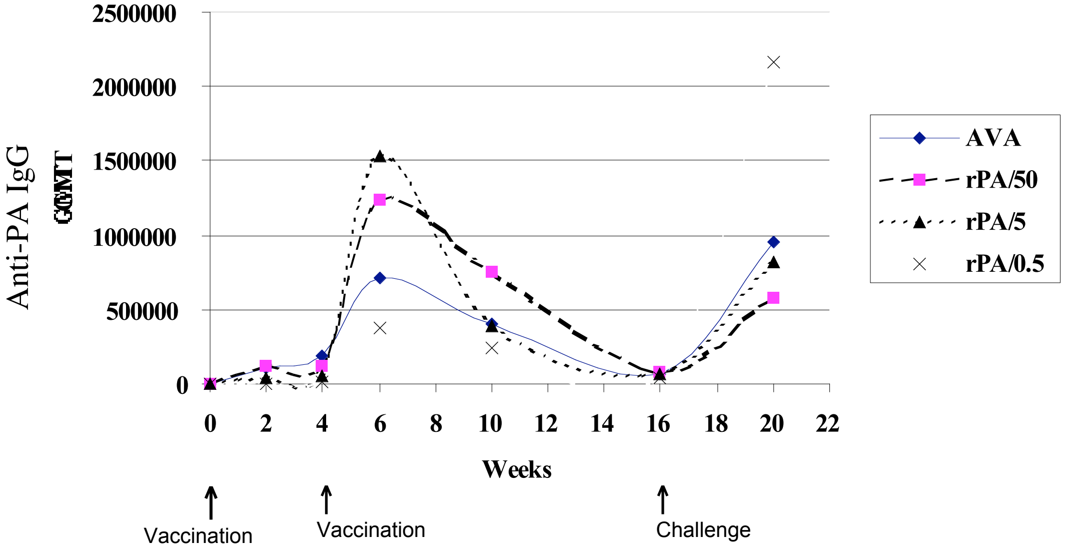

Figure 3. Anti-PA IgG titers in the Rabbits after administration of two doses of rPA or AVA

3.4.2 Efficacy Studies with *B. anthracis*-derived rPA in Nonhuman Primates

3.4.2.1 Single-dose Study in Nonhuman Primates

Another laboratory preparation of *B. anthracis*-derived rPA was tested in nonhuman primates. Rhesus monkeys were divided into two groups of 10, one group received 50 µg rPA adsorbed to Alhydrogel (725 _g aluminum/dose) and the other group received AVA (28). Three control rhesus monkeys received only Alhydrogel/PBS. The animals received only one dose IM and were exposed 6 weeks later to a lethal aerosol challenge with *B. anthracis* spores of the Ames strain (95 ± 53 LD_{50}).

All of the animals in both vaccine groups survived the challenge, whereas all of the three control animal died (Table 3).

Table 3. Challenge-Survival in Nonhuman Primates after rPA Single Dose

Group	Vaccine	Survived Challenge
1	AVA	10/10
2	50 µg rPA/AL	10/10
3	AL/PBS	0/3

The monkeys were bled at 2, 4, 5 and 6 weeks and anti-PA IgG titers were measured by ELISA (Figure 4).

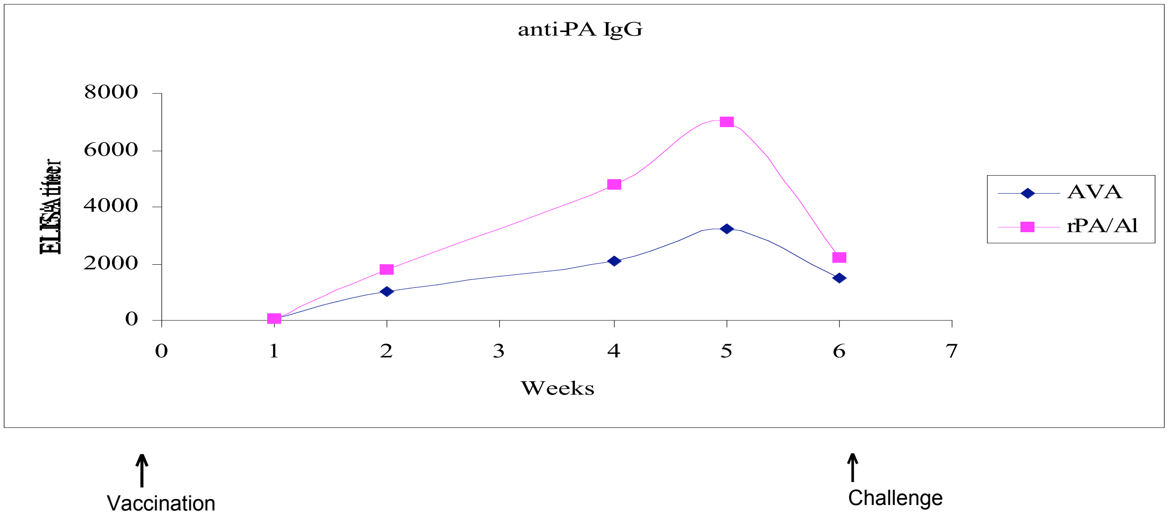

Figure 4. Anti-PA IgG to AVA and rPA in the Nonhuman Primate

Five weeks after immunization, animals were bled and serum was collected. Lethal toxin-neutralizing activity was assayed in J774A.1 cells (Table 4).

Table 4. Mean Toxin Neutralization Antibody (TNA) Titers in Rhesus Macaque, 5 Weeks After Vaccination

**Group**	**Vaccine**	**Geometric Mean TNA Titer**
1	AVA	424
2	PA + Alhydrogel	823
3	Controls	50

3.4.2.2 Dose-ranging Study in Nonhuman Primates

B. anthracis-derived rPA, pooled from three separate laboratory-scale preparations, was formulated in 50 mM ammonium acetate, pH 8.9, 325 mM NaCl, 2 mM EDTA, and 0.2 mM PMSF. Prior to injection, the rPA was formulated in Dulbecco's PBS (phosphate buffered saline) and adsorbed to Alhydrogel. Ten rhesus monkeys per group were vaccinated with 50, 5, or 0.5 _g of rPA adsorbed to Alhydrogel (725 _g aluminum/dose). The negative control group received Alhydrogel plus PBS, and the positive control group received AVA (lot FAV018). Animals were vaccinated by two IM injections (day 0 and 28), and were challenged by aerosol with a lethal dose (147 ± 82 LD_{50}) of *B. anthracis* spores of the Ames strain 3 months later (Table 5).

Five of five rhesus monkeys vaccinated with AVA and challenged survived with no bacteremia present post-challenge. A sixth monkey died during the vaccination period and prior to challenge and is not included in Table 5. For this monkey, the pathological examination could not establish a definitive cause of death. All negative control monkeys died between days 4 and 8 post-challenge.

Table 5. Challenge-Survival in Nonhuman Primates After Two Doses of rPA

**Group**	**Vaccine**	**Survived Challenge**
1	AVA	5/5
2	50 _g rPA/AL	9/9
3	5 _g rPA/AL	9/10
4	0.5 _g rPA/AL	9/10
5	AL/PBS	0/6

The monkeys were bled every week, and the anti-PA IgG titers were measured by ELISA (Figure 5).

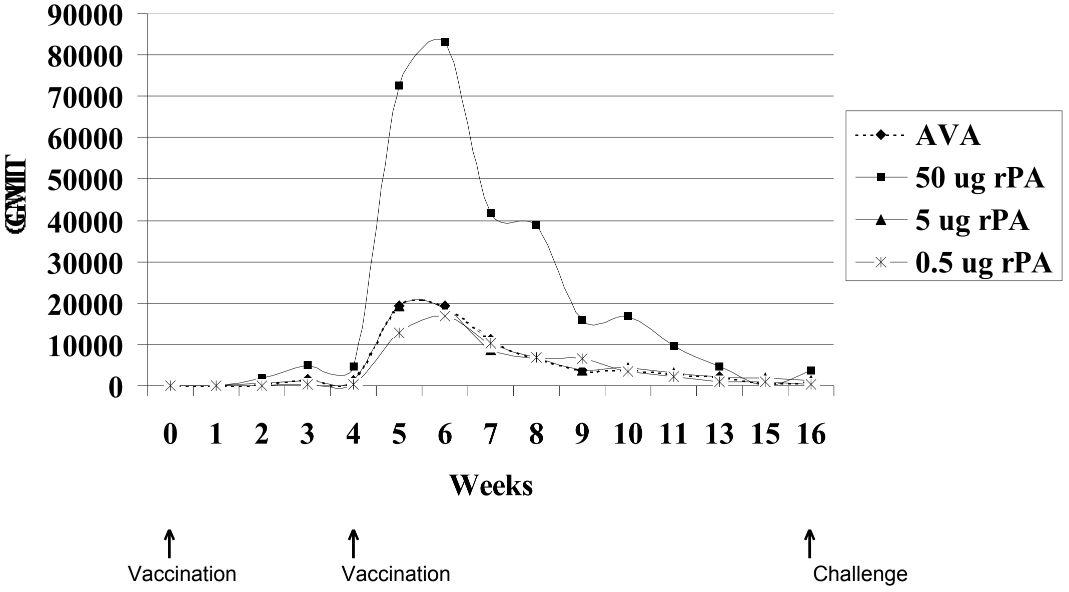

Figure 5. Anti-PA IgG Response to AVA and rPA in the Nonhuman Primate

3.5 History of rPA Expression in *E. coli* and the Involvement of WRAIR in the Development of a New Anthrax Vaccine

As the functionality of the anthrax toxin components on the molecular level has been discovered (17,18), and the functional domains of PA and LF have been defined, it was possible to construct chimeric LF proteins which lacked toxicity but, in combination with PA, still permitted translocation of peptides or proteins into the cytosol of target cells such as macrophages and lymphocytes (30). It has been shown that this strategy may be used to induce cytotoxic T-lymphocyte (CTL) responses *in vivo* (31,32).

The Division of Retrovirology at the WRAIR, established a Cooperative Research and Development Agreement with AVANT Immunotherapeutics, to manufacture and evaluate a hybrid protein composed of the N-terminal of LF fused to the HIV p24 antigen (LFn-p24) in combination with rPA, as an HIV vaccine to induce proliferation of anti-gag CTLs. For this evaluation, both the rPA and the LFn-p24 are produced as recombinant proteins using *E. coli* as host for a state-of-the-art expression system. A small animal experiment using rPA in combination with LFn-p24 demonstrated high titer anti-PA IgG ELISA titers.

The availability of rPA derived from *E. coli* for the development of a new anthrax vaccine based on purified PA, provides an opportunity to address potential interventions for the challenge of anthrax as a biological weapon and forms the basis for this protocol.

A general safety study has been performed using GMP manufactured and vialed rPA derived from *E. coli*. Mice and guinea pigs were administered an intraperitoneal injection of rPA. No overt signs of ill health (or unusual responses), death or weight loss during the test period were observed.

The *E. coli*-derived recombinant Protective Antigen (rPA) is very similar to the rPA derived from the avirulent, non-toxigenic, non-sporulating *B. anthracis* strain (see Section 3.4). *E. coli*-derived rPA has been utilized in fewer animal model challenge experiments, as described below and in the Investigator's Brochure (IB). However, currently available data in mice and New Zealand White (NZW) rabbits suggest that vaccination with *E.coli* derived rPA produces immune responses, which are qualitatively and quantitatively indistinguishable from the *B. anthracis*-derived product.

3.6 Non Clinical Studies conducted with *E. coli*- derived rPA Vaccine

Animal experiments have been conducted to demonstrate that the *B. anthracis*-derived rPA is effective as the immunogenic component of an anthrax vaccine (see Section 3.4). *In vitro* experiments have shown that the *E. coli*-derived rPA is biologically active and comparable to the *B. anthracis*-derived rPA. Therefore, the following two studies were designed to compare the *E. coli*-derived rPA to the *B. anthracis*-derived rPA *in vivo*.

3.6.1 Study in Rabbits Comparing *E. coli*-derived rPA / Alhydrogel to *B. anthracis*-derived rPA / Alhydrogel

NZW rabbits (24 per group) were given one dose of rPA followed by an extremely high aerosol challenge dose (449 LD_{50}) to maximize any statistical difference observed between groups.

The rabbits were inoculated at day 0 with 25 _g of either *E. coli*-derived rPA or *B. anthracis*-derived rPA both adsorbed to Alhydrogel (725 _g aluminum/dose). At day 28, the rabbits were challenged by aerosol exposure. For the *B. anthracis*-derived rPA, 14/24 animals survived, and for the *E. coli*-derived rPA, 12/24 animals survived. There was no statistical difference in survival between the two groups of animals, and all of the 8 control animals died. The mean titers from the anti-PA IgG ELISA two weeks after inoculation were 41.6 µg/ml for the *B. anthracis*-derived rPA and 38.7 _g /ml for the *E. coli*-derived rPA. The mean titers one week later (at three weeks after inoculation) were 25.6 _g g/ml and 28.2 _g /ml for the *B. anthracis* and *E. coli*-derived rPA, respectively.

This study demonstrated that the *E. coli*-derived rPA was comparable in anti-PA IgG response to the *B. anthracis*-derived rPA.

3.6.2 Studies in Mice Comparing E. coli-derived rPA to B. anthracis -derived rPA and Comparing to Alhydrogel

Immunogenicity studies in Balb/c (H-2d) mice were conducted to examine the effect of rPA alone and rPA adsorbed to Alhydrogel (0.28 mg aluminum)on the production of anti-PA IgG.

Both *B. anthracis*- and *E. coli*-derived rPA were used in this study, as summarized in Table 6.

Table 6. rPA Immunogenicity Study

Source of rPA	Amount of rPA	Adjuvant
B. anthracis	4 μg	None
B. anthracis	4 μg	Alhydrogel
E. coli	4 μg	None
E. coli	4 μg	Alhydrogel
B. anthracis	20 μg	None
B. anthracis	20 μg	Alhydrogel
E. coli	20 μg	None
E. coli	20 μg	Alhydrogel

Mice (5 per group) were immunized at 0 and 28 days with 4 μg or 20 μg of rPA and production of serum anti-PA IgG was measured for 15 weeks using the anti-PA IgG ELISA. GMTs of the 4 μg dose group are summarized in Figure 6, and GMTs of the 20 μg dose group are summarized in Figure 7.

Figure 6. Production of anti-PA IgG in Balb/c, H-2d mice following two doses (0 and 28 days) of vaccine containing 4 μg of rPA alone or in combination with Alhydrogel.

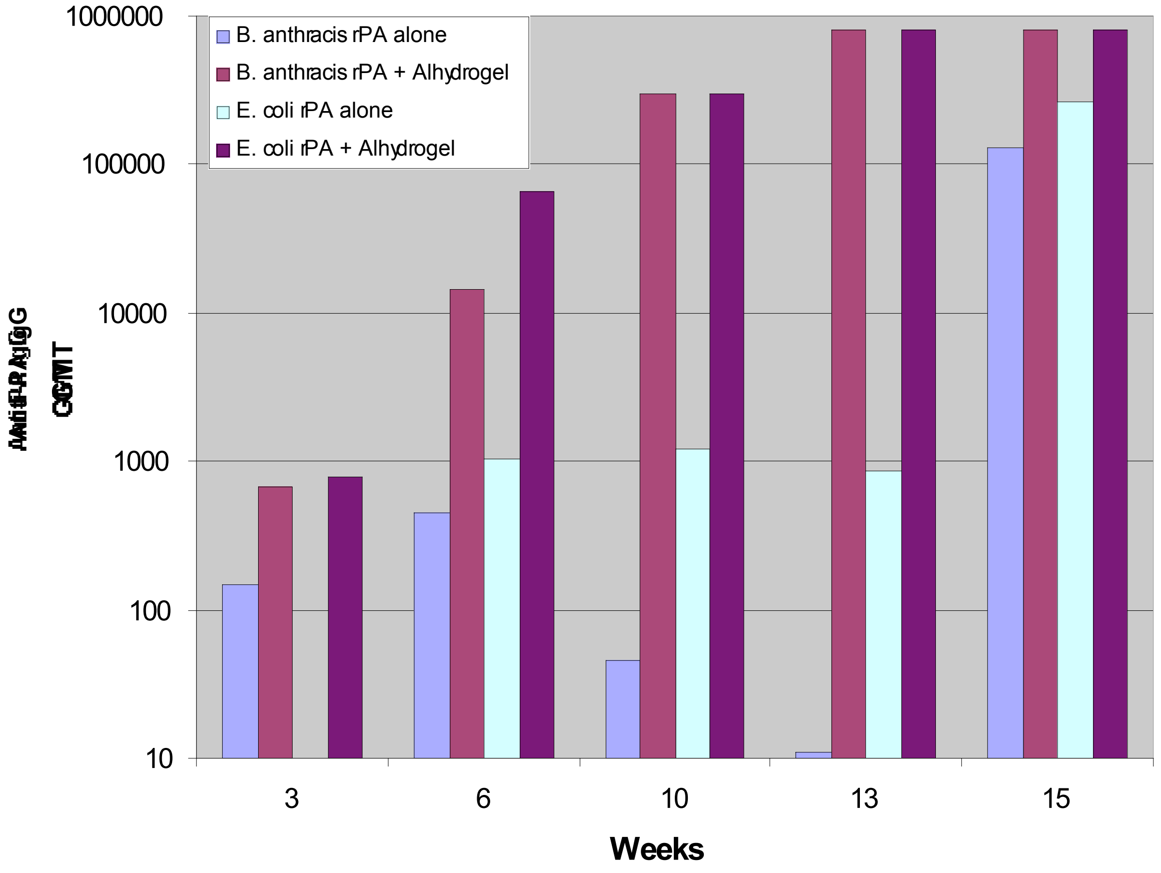

Figure 7. Production of anti-PA IgG in Balb/c, H-2d mice following two doses (0 and 28 days) of vaccine containing 20 µg of rPA alone or in combination with Alhydrogel.

Both studies indicate that *E. coli* derived rPA is comparable to *B. anthracis*-derived rPA. Also the addition of Alhydrogel improves the kinetics of antibody response.

3.7 ALHYDROGEL:

3.7.1 Alhydrogel

Alhydrogel (Al_2O_3) is a specific preparation of the mineral salt adjuvant aluminum hydroxide (35) and its use in human vaccines is well established. The adjuvant in AVA (Anthrax Vaccine Adsorbed) is aluminum hydroxide ($Al(OH)_3$), a preparation with less reproducible physico-chemical properties and thus more batch-to-batch variation.

4.0 STUDY OBJECTIVES

4.1 Primary Study Objectives:

- Evaluate the safety of rPA at 4 different doses of rPA with or without Alhydrogel.
- Evaluate the immunogenicity of 4 different doses of rPA with or without Alhydrogel

4.2 Primary Study Endpoints:

- Frequency and severity of local and systemic common adverse events following vaccination at 4 different doses of rPA with PBS or with Alhydrogel.
- IgG antibody titers to PA as measured by the anti-PA IgG ELISA (enzyme-linked immunosorbant assay)
- Toxin neutralizing antibody production as measured by the TNA (toxin neutralizing antibody) assay

4.3 Immunologic Studies:

Assays that will be used to evaluate the humoral immune response to rPA include the serum anti-rPA immunoglobulin G (IgG) enzyme-linked immunosorbent assay (ELISA), the serum toxin neutralizing antibody (TNA) assay and the rPA-specific B cell antibody secreting cell assay (ASC). Blood will also be drawn to assess Cell-Mediated Immunity (CMI). Antigen-specific CD4 T Cell helper responses will be assessed using a Lymphocyte Proliferation Assay LPA). Antigen-specific CD8 T cell responses will be measured using a flow cytometric-based assay to measure cell-specific intracellular γ -interferon cytokine (ICC) as well as an enzyme-linked immunosorbent SPOT assay measuring γ -interferon secretion (ELISPOT).

5.0 INVESTIGATIONAL PLAN

5.1 Overall Study Design and Plan

5.1.1 Recruiting of Anthrax Vaccine Naïve Volunteers

Healthy volunteers between 18 and 40 years old will be recruited using flyers, newspaper advertising, and direct mailing at local military installations, and to the general population of the greater Washington D.C. area.

In addition, the advertisement will state that the vaccine is experimental and is not known to provide protection against anthrax disease.

Military personnel will not be permitted to volunteer for the study. The reason military will be excluded is the potential requirement for subsequent vaccination with AVA, the licensed anthrax

vaccine. The potential risk and benefit of serial immunization with an experimental recombinant anthrax vaccine followed by the AVA is unknown and merits independent, controlled evaluation after the safety and efficacy of a recombinant product is demonstrated. Finally, individuals who may have been exposed to anthrax, who received anthrax vaccine or who are known to have had anthrax will be excluded for this study which seeks to define immunogenicity in true anthrax naïve persons.

5.1.2 Screening and Informed Consent Procedures

Study Volunteers will receive a briefing from the PI (Principal Investigator) or an associate investigator. The content of the briefing is provided as Appendix 17.2. The briefing is followed by an opportunity for questions from the volunteers. Study nurses will then review the consent form individually with potential volunteers and answer any questions. After review with the study nurse, an Informed Consent Form (see Appendix 17.3) will be signed, and a Test of Understanding (see Appendix 17.4) will be completed by all volunteers, prior to enrollment in the study. The test of understanding is reviewed "one-on-one" with the volunteer and a member of the study team. To determine eligibility, volunteers who have given written informed consent and have passed the test of understanding, will undergo a complete medical history and physical examination and screening laboratory assessments. During this evaluation, additional questions or concerns will be elicited from the volunteer.

Volunteers who qualify for inclusion into the study will be contacted after screening laboratory results are available, and if eligible, they will return to the clinic for a second screening visit. This will allow study staff to evaluate the reliability of volunteers, increase coordination for the vaccine visits, re-enforce the need for compliance on the designated vaccination days and review eligibility test results in person. Volunteers will be scheduled for an appointment for the initial vaccination visit.

Volunteers who do not qualify for the study may be asked to return to the clinic for counseling or discussion of lab results and appropriate referral as needed.

Women of child bearing potential will undergo serum pregnancy testing at screening, on the day of each immunization, and at the final visit (Table 9, Section 5.5.6).

5.1.3 Assignment to Treatment Group

Within Groups III to VIII (double-blind, placebo-controlled part), volunteers are randomized to placebo or active agent, according to a randomization list generated by the senior data manager.

To maintain the blind, this randomization list will only be available to the study pharmacist, the data manager, and, if required on behalf of the study volunteer's safety, the Local Medical Monitor. Vaccine will be delivered IM in the deltoid muscle at the intervals shown in the Study Flow Chart (Table 9, Section 5.5.6).

5.1.4 Recording of Acute Adverse Experiences and Maintenance of Study volunteer's Diary

Volunteers will be observed for 30 minutes following injection for acute adverse experiences. They will be instructed to maintain a diary of local and systemic reactions for 7 days post-

immunization. They will be provided with a ruler to measure erythema, induration or other observable reactions. In addition, volunteers will be provided with a thermometer to assess temperature on a daily basis. The study team will review the procedures with volunteers to ensure accuracy and completion of the diary card and will be given emergency contact information prior to volunteer departure from clinic.

The study nurse or designee will contact all volunteers by telephone on the day following each vaccination to determine if any reactions have occurred. If significant reactions are reported by the volunteer following vaccination, the volunteer will be instructed to return to the clinic for evaluation and safety labs will be obtained.

At 48-72 hours after each immunization, the volunteers will return to the clinic for an interval evaluation and laboratory measurements of hematology, serum chemistry, and urinalysis to assess safety

Two weeks after each vaccination, all volunteers will undergo a directed medical evaluation with a physician. To assess safety, the physician will review the diary card, conduct interval history and physical exam, and collect specimens for laboratory measurements of hematology, serum chemistry, and urinalysis to assess safety. Volunteers will be encouraged to report any adverse events at any time and may be asked to come into the clinic for evaluation by the research medical team. All adverse events will be recorded, and the relationship to the test product will be assigned by the PI and reviewed by the Local Medical Monitor. All adverse events will be tabulated by relationship to study product, organ system, severity and temporal relation to vaccination.

5.1.5 Criteria for Suspension of Vaccine Administration (“Stopping Rules”)

Since this is the first human experience with *E. coli*-derived rPA, “stopping rules” (i.e. criteria for suspension of vaccine administration) as outlined below were established to closely link the review of adverse experiences to study conduct:

5.1.5.1 Suspension of Vaccine Administration within a Treatment Group

- Vaccine administrations within a treatment group will be suspended after report of a single Grade 2, vaccine –related (possibly, probably, definitely) adverse event unless the Principal Investigator, Local Medical Monitor, and DVC Medical Monitor agree that the specific event does not pose an additional risk to the individual or to the study population.

- In the event of a second Grade 2 adverse event (possibly, probably, or definitely related) in the same (or related body system) or a single Grade 3 or 4 adverse event (possibly, probably, or definitely related) the study must be suspended pending notification of the IRB, HSRRB, HURC, and FDA.

- Vaccine administrations will resume pending the outcome of the review and upon verbal and or written approval from the respective entities.

5.1.5.2 Suspension of ALL Vaccine Administrations

- Vaccine administrations for all treatment groups will be suspended after report of a single Grade 2, vaccine –related (possibly, probably, definitely) adverse event unless the Principal Investigator, Local Medical Monitor, and DVC Medical Monitor agree that the specific event does not pose an additional risk to the individual or to the study population.

- In the event of a second Grade 2 adverse event (possibly, probably, or definitely related) in the same (or related body system) or a single Grade 3 or 4 adverse event (possibly, probably, or definitely related) the study must be suspended pending notification of the IRB, HSRRB, HURC, and FDA.

- Vaccine administrations will resume pending the outcome of the review and upon verbal and or written approval from the respective entities.

5.1.5.3 Premature Termination of the Clinical Trial

- The clinical trial will be suspended and all vaccine administrations **will be halted** if two study volunteers experience a related (possibly, probably, definitely) adverse event of Grade 2 or higher with the **same treatment group**.

- The causality relationship of the adverse event will be reviewed by the PI, Local Medical Monitor, DVC Medical Monitor, HURC, HSRRB and FDA.

- Vaccine administrations may resume pending the outcome of the review and upon verbal and or written approval from the PI, Local Medical Monitor, DVC Medical Monitor, HURC, HSRRB and FDA.

5.1.6 Dose-Escalation and Progression to the Next Treatment Group (Dose-Level)

Table 7 summarizes the treatment groups for this dose-escalation study, which evaluates the rPA vaccine with PBS and rPA adsorbed to Alhydrogel. Groups I and II pertain to the open-label part of the study, and the other 6 groups to the double-blind placebo-controlled part.

Table 7 Summary of Treatment Groups for the Dose-Escalation of rPA (With or Without Adjuvant)

Phase	Group No.	Study Group Vaccination at 0 and 1 month	No. Volunteers	No. Placebo Volunteers
Open	Group I	5 μg rPA with PBS	5	0
Label	Group II	5 μg rPA with Alhydrogel	5	0
Randomized	Group III	25 μg rPA with PBS	8	2

**Double Blind**				
	**Group IV**	**25 μg rPA with Alhydrogel**	**8**	**2**
	**Group V**	**50 μg rPA with PBS**	**8**	**2**
	**Group VI**	**50 μg rPA with Alhydrogel**	**8**	**2**
	**Group VII**	**100 μg rPA with PBS**	**8**	**2**
	**Group VIII**	**100 μg rPA with Alhydrogel**	**8**	**2**
▼	**TOTAL**		**58**	**12**

The outlined order of treatment groups was designed to ensure that at each new dose-level of rPA, the vaccine would first be administered without adjuvant (rPA with PBS; Groups I, III, V, VII) then adsorbed to Alhydrogel (Groups II, IV, VI, VIII).

Volunteers in each dose group will receive two IM injections at Day 0 and one month as outlined in Table 7.

The use of placebo recipients, who will receive PBS alone, is primarily to provide negative controls for the cellular immunity assays.

In addition to the stopping rules outlined in section 5.1.5 above, the following rules for progressing to the next treatment group were established, in order to closely link the review of adverse experiences to study conduct.

5.1.6.1 Open label phase (Groups I –II)

Before progressing from Treatment Group I to Group II, the PI or an associate investigator must document that there were NO safety or tolerability concerns related to the **first vaccination** during the 24 hr follow up telephone contact, the 48-72 hr visit (visit 4) and study visit 5 (14 days) for each study volunteer enrolled in Group I.

Before progressing from Treatment Group II to Group III, the PI or an associate investigator must document that there were NO safety or tolerability concerns related to the **second vaccination** during the 24 hr follow up telephone contact, the 48-72 hr visit (visit 4) and study visit 8 (14 days) for each study volunteer enrolled in Group I and II.

5.1.6.2 Randomized Double Blind Phase (Groups III-VIII)

Progression from rPA and PBS to rPA and Alhydrogel within a dosing cohort may occur following the first vaccination. The PI or an associate investigator must document during the 24 hr follow up telephone contact, the 48-72 hr visit and study visit 5 (14 days) that there were NO safety or tolerability concerns related to the **first vaccination**.

Progression to the next higher dosing cohort will not occur until the safety results from the 14 day visit after the **second vaccination** have been reviewed for all study volunteers within the group. For example, progression from Group IV (25 ug rPA and Alhydrogel) to Group V (50 ug rPA with PBS) will not occur until the safety results have been reviewed from study visit number 8, which occurs 14 days after the second vaccination has been given to the Group IV subjects.

5.2 Rationale for the Immunization Schedule and Study Groups

The dosing regimen for AVA is inconvenient, expensive and may permit an unacceptable window of susceptibility before boosting achieves potentially protective titers. There is substantial literature on protein vaccines in children and adults to provide some generalizations regarding potential dosing schedules for rPA. Compressed schedules achieve high titer antibody responses earlier than extended schedules. However, dosing schedules extended over 6-12 months achieve higher peak titers and better durability of antibody responses. In many settings, particularly post-exposure prophylaxis, rapidly achieving protective antibody responses is critical.

The Day 0, and Month 1 dose regimen represents a compromise between rapid and 6-12 month dosing regimens as used for AVA. It is desirable to achieve the highest possible and most durable antibody titers with a minimum number of immunizations over the shortest time possible. The current study will provide useful data of an intermediate approach as well as critical safety and immunogenicity data. It is anticipated that the optimal doses derived from this study will be used subsequently in a formal schedule assessment study comparing different regimens.

Since this vaccine has never been used in humans, four doses of rPA will be tested in a dose escalating fashion (5 _g, 25 _g, 50 _g and 100 _g). The selection of these four doses of rPA for human use is based on data from animal challenge experiments (same dose range) summarized in the Investigator's Brochure. The dose of Alhydrogel is based upon human experience of safety, tolerability and induction of humoral immunity.

5.3 Assessment of Immunogenicity

5.3.1 Primary Measures of Immunogenicity

Immunogenicity Endpoints: Description of the following primary measures of immunogenicity:

Humoral
1) Anti-PA (rPA and native PA) antibody as measured by ELISA
2) Toxin neutralizing antibody production as measured by the TNA assay

Cellular
(1) Antigen-specific CD4 T Cell helper responses will be assessed using a Lymphocyte Proliferation Assay LPA). Antigen will include whole rPA. Additional controls to include positive antigen control (Tetanus toxoid), negative antigen control and mitogens.

(2) Antigen-specific CD8 T cell responses will be measured using a flow cytometric-based assay to measure cell-specific intracellular γ -interferon cytokine (ICC). Individual and/or pools of 15-20 mer synthetic peptides will be used to stimulate T cells for γ -interferon production. Alternatively, or in addition, Antigen-specific CD8 T cell responses will be measured using an enzyme-linked immunosorbent SPOT assay measuring γ -interferon secretion (ELISPOT).

5.4 Selection of Study Populations

The total study population consists of 70 healthy adults. Study volunteers will be 18 through 40 years of age.

Women will agree to practice effective contraception prior to study entry and until 3 months after the second immunization. Study volunteers will be followed for 11 months after the second immunization and will then have their study termination visit according to the protocol schedule of events. Two screening visits will determine eligibility for trial entry based on the criteria listed in Sections 5.4.1. and 5.4.2. If more than 45 days have elapsed between the second screening visit and the anticipated day of first vaccination, repeat safety laboratories will be performed. If a delay in screening or first vaccination visit (Visit 3, day 0) occurs, respective volunteers will be re-screened and re-consented.

Military personnel and reservists *will not* be enrolled to insure that they may safely receive the licensed anthrax vaccine, AVA, if so directed by command authority.

5.4.1 Inclusion Criteria

Volunteers are eligible for this study if they meet all the following criteria:

1. Healthy Citizen or Permanent Residents of the U.S.
2. Age 18 to 40 years.
3. For women, a negative serum pregnancy test will be required at study entry and within 24 hours prior to each vaccination, as well as verbal assurance that adequate birth control measures are applied prior to initial vaccination and for 3 months after the last vaccination.
4. Good health as determined by medical history, physical examination, and clinical judgment.
5. Normal Baseline Clinical Laboratory Values at screening including:

- Complete Blood Count (CBC) including:
  - White Blood Cell Count: 3.8 -10.8
  - Red Blood Cell Count (Mill/MCL)
    - Male: 4.20 - 5.80
    - Female: 3.80 - 5.10
  - Hemoglobin (G/DL)
    - Male: 13.2 - 17.1
    - Female: 11.7 – 15.5
  - Hematocrit (%)
    - Male: 38.5- 50.0
    - Female: 35.0 - 45.0
  - Platelet Count: 140 – 440 (THOUS/MCL)
  - Differential
- Urine dipstick for protein and blood: negative or trace. If either is ≥ 1+, obtain complete urinalysis (UA). If microscopic UA confirms evidence of hematuria or proteinuria ≥ 1+, the volunteer is ineligible.
- Negative serology for HIV infection (ELISA test).
- CPK $\leq$ 300 with no evidence of clinical disease
- Hepatic Function Tests including AST, ALT, ALK PHOS.
- Total bilirubin, BUN, serum creatinine, serum electrolytes (BUN and Creatinine may be below the lower limit of normal)

6. Availability for at least 13 months of follow-up from the time of the screening visit.
7. Successful completion of the Test of Understanding defined as 90% correct with three opportunities to take test. Errors will be reviewed with volunteer after each test.
8. Commitment for trial participation and signature of the approved consent form.

<u>5.4.2 Exclusion Criteria</u>

Individuals will not be enrolled into the study if they meet at least one of the following criteria:

1. Have a history of anthrax disease or receipt of anthrax vaccine.
2. Have active tuberculosis or other systemic infectious process by review of systems and physical examination.
3. Laboratory evidence of active hepatitis B or C.
4. Have a history of immunodeficiency, chronic illness requiring continuous or frequent medical intervention, autoimmune disease, use of immunosuppressive medications, or any known history of cardiac disease.
5. Have evidence of psychiatric, medical and/or substance abuse problems during the past 6 months that the investigator believes would adversely affect the volunteer’s ability to participate in the trial.
6. Have occupational or other responsibilities that would prevent completion of participation in the study.
7. Licensed vaccines are not exclusionary but should be given at least 2 weeks before or after immunization (if live vaccine: 60 days before or after immunization) to avoid potential confusion of adverse reactions.
8. Have used experimental therapeutic agents within 30 days of study entry and through the entire 12-month period of on-study evaluation.
9. Have a history of anaphylaxis or other serious adverse reactions to vaccines.
10. Are pregnant or lactating.
11. Are or have been on cancer chemotherapy.
12. Are receiving ongoing therapy with immunosuppressive therapy such as systemic corticosteroids within 3 months prior to the study. Inhaled and topical steroids are permitted. Also “burst” therapy of steroids is permitted except within two weeks prior to vaccination.
13. Are found to be HIV infected during screening, or become HIV infected during the study.
14. Are a member of the U.S. military or reservist.
15. US military or reservist who may receive the licensed anthrax vaccine BioThrax™.
16. US military or reservist who served in theater during the Persian Gulf War during the period of January 1991 through May 1991.
17. <u>You may not participate in the study if your occupation may lead to an exposure to anthrax or may require you to take the licensed anthrax vaccine, for example postal workers.</u>

<u>5.4.3 Withdrawal of Study volunteers from Trial and/or Assessment</u>

A study volunteer’s may withdraw his/her consent to participate in the study at any time without prejudice. Additionally, the investigator may withdraw a study volunteer’s if, in his/her clinical judgment, it is in the best interest of the study volunteer or if the study volunteer cannot comply with the protocol. Participants will be removed from active vaccination if they become pregnant, become HIV positive, receive immunomodulators or experimental agents contrary to the

inclusion/exclusion criteria or must receive the licensed AVA vaccine for anthrax. Volunteers may not participate in interventional experimental trials until the study is complete. Wherever possible, the tests and evaluations listed for the termination visit should be carried out if the study volunteer refuses follow-up according to the protocol visit schedule. The Sponsor should be notified of all study withdrawals within 24 hours. Study volunteers who withdraw from the study will be replaced only as long as the study is still open for enrollment.

All study volunteers who receive at least one immunization will be included in the safety analysis. Only study volunteers who have received two injections and have had the visit 8 blood draw (2 weeks post second injection) will be included in the final immunogenicity analysis, though immunogenicity data may be used in the analysis of earlier time points. If a study volunteer's misses an immunization visit, no further immunizations will be performed, but the study volunteer will continue to be followed according to the protocol visit schedule.

If a study volunteer's misses a non-immunization visit, immunization will continue as planned. A missed visit is defined according to the time window listed by visit in section 5.5.6. If a study volunteer's does not complete the immunization schedule secondary to a serious adverse event or toxicity, he or she will continue to be followed according to the protocol visit schedule, and, at a minimum, until the adverse event/toxicity is resolved and/or the cause is identified.

A genuine effort will be made to determine the reason(s) why a study volunteer's fails to return for the necessary visits. If the study volunteer is unreachable by telephone, a registered letter, at the minimum, will be sent to the study volunteer requesting contact with the clinic. This information will be recorded on the appropriate source document. For study volunteers who become pregnant before completing the vaccine series, no further immunizations will be given regardless of outcome. The study volunteer will be followed for all remaining scheduled visits according to the schedule of procedures for safety evaluation. For all study volunteers who become pregnant while participating in the trial, a Pregnancy Case Report Form (CRF) will be completed. The site will maintain contact with pregnant study volunteers to obtain pregnancy outcome information for the Pregnancy Follow-up CRF (see Appendix 17.5).

Should a study volunteer withdraw or be withdrawn from the study for any reason, the volunteer will be requested to undergo clinical test(s) and examination(s) as determined by the attending physician as necessary for the well being and health of the volunteer. These tests or examinations may include any of those listed in the study flowchart, page 6, or any additional studies as determined as necessary for the health and well being of the volunteer.

5.4.4 Replacement of Study volunteers

If a study volunteer's withdraws from the study, the principal investigator/study coordinator must contact the Data Coordinating Analysis Center (DCAC) to receive a replacement number. Replacement will only be permitted during the active enrollment phase. A fax or telephone message must be sent to the WRAIR clinical monitor providing the study volunteer identification number of the withdrawn participant.

5.5 Administration of Vaccine and Placebo

5.5.1 Summary of Test Articles

Production of the rPA component was performed by Cambrex BioScience Inc. Cambrex BioScience Inc. is an FDA-inspected CGMP manufacturer with a facility designed for commercial contract use. rPA was vialed at 100 μg/mL and 300 μg/mL at the WRAIR Pilot BioProduction Facility.

Alhydrogel is an aluminum hydroxide preparation manufactured under CGMP by HCI BioSector and supplied as a sterile product for injection by E.M. Sergent.

5.5.1.1 Recombinant Protective Antigen

Recombinant PA derived from a commercially available, non-pathogenic, laboratory strain of *B. anthracis* was produced in *E. coli*. Recombinant PA is expressed as a single polypeptide chain of 767 amino acids. It was produced in 12 liter scale and purified to >90% purity by 4 sequential column chromatographic steps. The bulk rPA was formulated (25 mM Sodium Phosphate, 150 mM NaCl, pH 8.0) and vialed at two separate doses by WRAIR. In addition, diluent was vialed and blank vials were processed by the WRAIR Pilot BioProduction Facility (Forest Glen, MD) in July/August 2001. rPA is vialed at 100 μg/mL and 300 μg/mL to increase dosing accuracy.

5.5.1.2 Alhydrogel

Alhydrogel, 1.3% solution of aluminum hydroxide (6.4 mg elemental aluminum/mL) is manufactured under CGMP by HCI BioSector and supplied as a sterile product for injection by E.M. Sergeant.

5.5.2 Formulation of rPA Anthrax Vaccine

For the Phase 1 clinical trial, rPA will be adsorbed to Alhydrogel within 12 hours of administration. Preliminary studies conducted at USAMRIID indicated that less than 60% of the rPA bound to the Alhydrogel in 25 mM sodium phosphate, 150 mM NaCl, pH 8.0. However, when the phosphate concentration was reduced to approximately 8.5 mM, greater than 90% of the rPA bound to the Alhydrogel. Since the rPA Final Drug Product is in 25 mM sodium phosphate, 150 mM NaCl, pH 8.0, the rPA Final Drug Product will be diluted in saline to obtain a final phosphate concentration of 8.33 mM upon mixing with Alhydrogel to facilitate adsorption of rPA to Alhydrogel.

Formulation of the rPA vaccine doses for the Phase 1 clinical trial will occur within 12 hours of administration of the vaccine. To obtain the correct formulations, the rPA Final Drug Product will be:

1) Diluted to the appropriate concentration and injected
2) Diluted, adsorbed to Alhydrogel and injected

After the vaccine formulations have been prepared, they will be stored at 4°C and used within 12 hours. Table 8 lists the dilution schemes and formulations to be used in the Phase 1 clinical trial. The final dose of vaccine contains 725 μg of elemental aluminum.

Table 8 Formulation of the rPA Anthrax Vaccine

Formulation	# Doses Prepared	rPA 0.1 mg/ml (ml)	rPA 0.3 mg/ml (ml)	Sodium Phosphate Buffer* (ml)	Saline 0.9% NaCl (ml)	Adjuvant 1.3% Alhydrogel (ml)	Final Phosphate Conc (mM)
5 ug rPA	9	0.50	0.00	6.50	3.00	0.0	17.50
5 ug rPA + Alhydrogel	9	0.50	0.00	2.83	5.54	1.13	8.33
25 ug rPA	2	0.63	0.00	1.12	0.75	0.00	17.50
25 ug rPA + Alhydrogel	2	0.63	0.00	0.20	1.38	0.29	8.33
50 ug rPA	1	0.70	0.00	0.28	0.42	0.00	17.5
50 ug rPA + Alhydrogel	4	0.00	0.70	0.70	2.33	0.47	8.33
100 ug rPA	2	0.00	0.70	0.77	0.63	0.00	17.5
100 ug rPA + Alhydrogel	2	0.00	0.70	0.00	1.16	0.24	8.33

*Sodium Phosphate Buffer = 25 mM sodium phosphate, 150 mM NaCl, pH 8.0

5.5.2.1 Procedure for Reconstitution

rPA with PBS and rPA with Alhydrogel will be prepared according to DVC SSP number 01-rPA-EC-02 (appendix 17.8) under a BSL-II safety hood by a trained technician or pharmacist. Alhydrogel mixtures will be prepared and adsorbed on ice for a minimum of 30 minutes using a single vial to accommodate injections for 1-10 volunteers. The formulated vaccine(s) will be used within 12 hrs of reconstitution. This will insure accuracy of formulation and conserve materials. The rPA is frozen and only needs to be thawed. The other constituents are provided in sterile unit dose vials and will be combined into a single syringe.

5.5.2.2 Dosage, Administration, and Labeling

To maintain blinding, a pharmacist (or a technician not involved in the evaluation of the study volunteers) will mix each dose, ensuring that the vaccine doses within and between each group have a similar injection volume.

Doses of all test articles are to be maintained at 4°C in a secure refrigerator until needed and are to be administered within 12 hours of reconstitution of vaccine (See Section 5.5.2).

Contents of the syringe are to be administered (after preparation of the injection site with alcohol) by intramuscular injection (IM, 1 mL) into the left deltoid muscle.

The investigational product is labeled as follows:

<u>0.3 mg vial</u>

Therapore rPA (0.3 mg) vial number

BPR No. BPR-488-01 Lot nNo.:0894

Contents;0.8 mL +/- 1-% Storage:-80 +/- 10C

Caution: "New drug limited by Federal Law to investigational use."

Date of Mfg: 17 Jul 01

Manufactured by: WRAIR, Silver Spring, MD 20910

<u>0.1 mg vial</u>

Therapore rPA (0.1 mg/mL) vial number

BPR No. BPR-491-00 Lot nNo.:0851

Contents;0.8 mL +/- 1-% Storage:-80 +/- 10C

Caution: "New drug limited by Federal Law to investigational use."

Date of Mfg: 24 Jul 01

Manufactured by: WRAIR, Silver Spring, MD 20910

A 3-part label (for source document, case record form, syringe bag) will document the study volunteer's initials, the study number (3-digit), randomization number, date and time of vaccine preparation.

5.5.2.3 Disposition

The Investigator or designee will be responsible for the accountability of all clinical supplies, including keeping an inventory that documents receipt, dispensing and disposition of these supplies. The supplies and inventory record must be made available for inspection by DVC and any monitors reviewing study records on behalf of DVC.

All used vaccine vials must be retained in a secure refrigerator. The sponsor (DVC) will periodically audit the retained vaccine vials to ensure accurate dispensing/administration of the vaccines. The used vials will then be destroyed by WRAIR.

Upon completion or termination of the study, the Investigator will retain all remaining clinical supplies as directed by DVC along with a copy of the inventory record.

5.5.2.4 Precautions to be Observed in Administering Study Vaccine

Study vaccine must not be administered to individuals with hypersensitivity to any component of the vaccine (refer to Section 5.4.2.).

As with any parenteral vaccine, epinephrine, and corticosteroids must be available for immediate use should an immediate hypersensitivity reaction such as anaphylaxis occur. All study vaccines must be injected deep into muscle. **DO NOT** inject intravenously.

5.5.3 Method of Assigning Study volunteers to Study Groups

At the first screening visit, study volunteers will be assigned personal identification numbers (PIN) and screening numbers. The screening number will become the study number, after confirming the eligibility of a volunteer as defined in the inclusion and exclusion criteria (Sections 5.4.1 and 5.4.2). Thus, study numbers of volunteers actually included into the study might be discontinuous, lacking the screening numbers of study volunteers that are not eligible.

In general, volunteers are assigned to a group in consecutive order. Randomization to placebo or active agent occurs within each group by consecutive assignment according to a randomization list prepared by the study data manager. If an individual drops out of the study during active enrollment, that group and randomization slot can be replaced with the next available volunteer. Once active enrollment is completed, i.e. all required volunteers have been enrolled; replacements of drop-outs will not be permitted.

5.5.4 Controls/Blinding

Volunteers and investigative site personnel will be blinded within Groups III- VIII. An unblinded pharmacist or designee not involved with study volunteer's evaluation will prepare each vaccine dose and deliver the doses to the research nurse for injection**.** Within each group, the volume of injection will be consistent. The investigative site personnel, as well as WRAIR personnel involved in the monitoring or conduct of the trial, will be blinded to the study vaccine code. However, in case of an emergency situation occurring in one of the study volunteers, the code of this study volunteer's can be broken. The sponsor must be informed as soon as possible if a code is broken.

5.5.5 Prior and Concomitant Medications/Vaccines

The following medications are prohibited while the study volunteer is on the study:

- Immunomodulatory agents
- Immunosuppressive agents (i.e., systemic steroids, chemotherapy)
- Other experimental agents

Table 9 Study Flow Chart

**Study visit (SV)**<br>**Telephone Contact (TC)**	1	2	3	TC	4	5	6	TC	7	8	9	10	11	12	13
Day	- 1<br>Pre-Screening	-2<br>Final Screen	0	1	2-3	14	28 +/- 7	29	32-34	42	70	112	182	252	364
Week	14 - 60 days<br>pre vaccination	3 -45days<br>pre vaccination	0	24 hrs	48-72 hrs	2	4	24 hrs	48-72<br>hrs	6	10	16	26	36	52
Month			0	.1	0.25	0.5	1	1.1	1.25	1.5	2.5	4	6.5	9	13
**Administrative Requirement**															
Registration, Informed Consent and Test of Understanding	X														
Volunteer registry database sheet	X														X
**Immunization and Safety Monitoring**			**X**				**X**								
**Clinical Requirements**															
Telephone Contact				X				X							
Complete History & Physical	X														
Interim History &Physical and Assessment of AEs		X	X		X	X	X		X	X	X	X	X	X	X
Review and/or Collect Diary Card				X	X	X		X	X	X					
**Safety Labs:**															
HIV serology (4 mL clot tube)	4 mL														
Hepatitis serology (3 mL SST)	4 mL														
Troponin I and II (3 mL Heparin)			3 mL			3 mL				3 mL					
Safety Labs and UA *<br>(3 mL EDTA, 4 mL clot)	7 mL	7 mL	7 mL		7 mL	7 mL	7 mL		7 mL	7 mL	7 mL	7 mL	7 mL	7 mL	7 mL
Serum Pregnancy Test (no additional)	X		X				X								X
**Immunogenicity Labs**															
Anti-PA IgG ELISA/TNA (clot tubes)		10 mL	10 mL			10 mL	10 mL			10 mL	10 mL	10 mL	10 mL	10 mL	10 mL
CMI assays** (heparinized tubes)		30 mL	30 mL			30 mL				30 mL	30 mL	30 mL	30 mL	30 mL	30 mL
Sample for production of human monoclonal anti-PA antibodies										40 mL					
TOTAL Blood = 518 mL over 13 months	15 mL	47 mL	50 mL		7 mL	50 mL	17 mL		7 mL	90 mL	47 mL	47 mL	47 mL	47 mL	47 mL

*Safety Labs include: (4 mL red top) electrolytes, creatinine, CPK, AST, ALT, GGT, Ca, Phosphate, BUN, uric acid, total bilirubin, (3 mL EDTA) CBC w/differential, and urine dipstick analysis. # Safety Labs (blood draw) and UA done if necessary.** Assays for cell-mediated immune response (CMI) under development: LPA (lymphocyte Proliferation Assay), _-IFN ELISPOT (ELISA-based assay for quantifying gamma-interferon producing lymphocytes), ICC (Intracellular Cytokine Assay)

5.5.6 Study Procedures

5.5.6.1 Enrollment Visits 1 and 2.

Two screening visits will determine eligibility for trial inclusion based on the criteria listed in Sections 5.4.1. and 5.4.2. If more than 45 days have elapsed between the second screening visit and the anticipated day of first vaccination, repeat safety laboratories (CBC, complete serum chemistries as noted below, and UA) will be repeated. If a delay in screening or first vaccination visit (Visit 3, day 0) occurs secondary to product shortage, respective volunteers will be re-screened and re-consented.

- Review and obtain volunteer's signature on vaccine consent form. Provide a copy to the study volunteer.
- Complete Test of Understanding
- Complete history and physical.
- ELISA and if needed Western blot HIV serology (4 mL red top).
- CBC with differential, serum chemistries, Glucose, Urea nitrogen, Bilirubin, Creatinine, BUN/Cr ratio, Na, K, Chloride, CO2, AST (SGOT), ALT (SGPT), GGT, Uric acid, Cholesterol, Triglycerides, LDH, Phosphate, Calcium, Iron, Alkaline Phosphatase, Total Protein, Albumin, Globulin, (3 mL EDTA + 4 mL red top).
- CPK
- HbsAg, and Hep C
  (4 mL serum separator tube (SST) red top).
- Serum pregnancy test for female volunteers
- Urine Analysis (UA), with microscopic exam if UA abnormal.
- Collect cell and serum archives at second screening visit for Anti-PA IgG ELISA/TNA and CMI assays (30 mL for ACD and 10 mL red top)

5.5.6.2 Visit 3, Day 0: First Immunization:

- Review all screening data and inclusion/exclusion criteria to ensure eligibility.
- Perform serum pregnancy test for women within 24 hours before immunization. **(Results must be known prior to immunization.)**
- Obtain interim history since previous visit, including vital signs and symptoms assessment.
- Collect samples for UA and CBC, complete serum chemistries (3 mL EDTA, 4 mL red top), and Troponin I and II (3 mL Heparin).
- Collect cell and serum archives for Anti-PA IgG ELISA/TNA and CMI assays (30 mL for ACD and 10 mL red top)
- Archive cells, plasma, and serum.
- Assign study volunteers to treatment group using randomization code.
- **(All of above to be performed prior to immunization.)**
- Administer first injection in the left deltoid muscle by deep intramuscular (IM, 1 mL) route.

PROPRIETARY

- Examine the injection sites for local reactions at 30 minutes post-injection and instruct study volunteers in the evaluation of these local reactions; observe and instruct while the study volunteer takes temperature (as guided for subsequent evaluations); enter findings on appropriate CRF page.
- Study volunteers are instructed in measurement of local reactions and temperature assessment and will record daily symptoms for 7 days post-immunization (including the day of immunization), and will contact the investigator if any untoward effects occur.

5.5.6.3 Telephone Contact after the First Immunization (TC):

The study nurse or designee will contact all volunteers by telephone on the day following the vaccination to determine if any reactions have occurred. Volunteers will be queried using a predetermined review of systems/reactogenicity worksheet. If the adverse effects experienced by the volunteer are deemed “moderate or severe”, he/she will be asked to return to clinic for evaluation by the investigator and safety labs will be obtained. Additional contacts will be arranged as needed.

5.5.6.4 Visit 4, 48-72 Hour Safety Evaluation after the First Immunization:

Study volunteers will return to the clinic within 48 to 72 hours post-immunization for:

- Interim History and Physical
- AE elicitation and review of the diary card.
- Collect samples for safety labs: UA and CBC, complete serum chemistries (3 mL EDTA, 4 mL red top).

5.5.6.5 Visit 5, Week 2 (window ± 7 days) Safety and Immunogenicity:

- Interim History and Physical (AE elicitation) and review/collect diary card.
- Collect samples for UA and CBC, complete serum chemistries (3 mL EDTA, 4 mL red top), and I and II (3 mL Heparin).
- Collect cell and serum archives for Anti-PA IgG ELISA/TNA and CMI assays (30 mL for ACD and 10 mL red top)
- Archive cells, plasma, and serum.

5.5.6.6 Visit 6, Week 4 (window ± 7 days) Second Immunization:

- Interim History and Physical (AE elicitation).
- Perform serum pregnancy test for women within 24 hours before immunization. **[Results must be known prior to immunization.]**
- Collect samples for safety labs: UA and CBC, complete serum chemistries (3 mL EDTA, 4 mL red top).
- Collect samples for immunogenicity (one 10 mL red top): binding antibody.
- Archive cells, plasma, and serum. **(All of these are to be performed prior to second immunization.)**
- Administer second injection in the left deltoid muscle as deep intramuscular (IM, 1 mL).

- Examine the injection sites for local reactions at 30 minutes post-injection and instruct study volunteers in the evaluation of these local reactions; observe and instruct while the study volunteer takes temperature (as guided for subsequent evaluations); enter findings on appropriate CRF.
- Study volunteers are instructed in measurement of local reactions and temperature assessment and will record daily symptoms for 7 days post-immunization (including the day of immunization), and will contact the investigator if any untoward effects occur.

5.5.6.7 Telephone Contact after the Second Immunization (TC)

The study nurse or designee will contact all volunteers by telephone on the day following the vaccination to determine if any reactions have occurred. Volunteers will be queried using a predetermined review of systems/reactogenicity worksheet. If the adverse effects experienced by the volunteer are deemed "moderate or severe", he/she will be asked to return to clinic for evaluation by the investigator and safety labs will be obtained. Additional contacts will be arranged as needed.

5.5.6.8 Visit 7, 48-72 Hour Safety Evaluation after the Second Immunization:

Study volunteers will return to the clinic within 48 to 72 hours post-immunization for:

- Interim History and Physical
- AE elicitation and review of the diary card.
- Collect samples for safety labs: UA and CBC, complete serum chemistries (3 mL EDTA, 4 mL red top).

5.5.6.9 Visit 8, Week 6 (± 7 days) Safety and Immunogenicity Visit:

- Interim History and Physical (AE elicitation) and review/collect diary card
- Collect samples for UA and CBC, complete serum chemistries (3 mL EDTA, 4 mL red top), and Troponin I and II (3 mL Heparin).
- Collect cell and serum archives for Anti-PA IgG ELISA/TNA and CMI assays (30 mL for ACD and 10 mL red top)
- Collect sample for Monoclonal Antibodies (anti PA) 40mL.
- Archive cells, plasma, and serum.

5.5.6.10 Visit 9, **Week 10** (± 7 days) Safety and Immunogenicity Visit:

- Interim History and Physical (AE elicitation)
- Collect samples for safety labs: UA and CBC, complete serum chemistries (3 mL EDTA, 4 mL red top).
- Collect cell and serum archives for Anti-PA IgG ELISA/TNA and CMI assays (30 mL for ACD and 10 mL red top)
- Archive cells, plasma, and serum.

5.5.6.11 Visit 10, Week 16 (± 7 days) Safety and Immunogenicity Visit:

- Interim History and Physical (AE elicitation)
- Collect samples for safety labs: UA and CBC, complete serum chemistries (3 mL EDTA, 4 mL red top).
- Collect cell and serum archives for Anti-PA IgG ELISA/TNA and CMI assays (30 mL for ACD and 10 mL red top)
- Archive plasma and serum.

5.5.6.12 Visit 11, Week 26 (± 2 weeks) Clinical and Immunological Follow-up:

- Perform abbreviated history and physical examination.
- Collect samples for safety labs: UA and CBC, complete serum chemistries (3 mL EDTA, 4 mL red top).
- Collect cell and serum archives for Anti-PA IgG ELISA/TNA and CMI assays (30 mL for ACD and 10 mL red top)
- Archive cells, plasma, and serum.

5.5.6.13 Visits 12, Week 36 (± 2 weeks):

- Perform abbreviated history and physical examination.
- Collect sample for CBC (3 mL EDTA).
- Collect sample for complete serum chemistries (4 mL red top).
- Collect urine for dipstick protein/blood. If urine protein or blood is 1+ or greater, obtain a complete UA.
- Collect cell and serum archives for Anti-PA IgG ELISA/TNA and CMI assays (30 mL for ACD and 10 mL red top)
- Archive cells, plasma, and serum

5.5.6.14 Visit 13, Week 52 (± 2 weeks) Clinical and Immunological Follow-up:

- Perform abbreviated history and physical examination.
- Collect sample for CBC (3 mL EDTA).
- Collect sample for complete serum chemistries (4 mL red top).
- Collect urine for dipstick protein/blood. If urine protein or blood is 1+ or greater, obtain a complete UA.
- Collect serum for pregnancy test for women
- Collect cell and serum archives for Anti-PA IgG ELISA/TNA and CMI assays (30 mL for ACD and 10 mL red top)
- Archive cells, plasma, and serum

5.6 Study Variables and Their Measurement

5.61 Immunogenicity Variables

5.6.1.1 Humoral Immune Responses to rPA:

Anti-PA IgG ELISA

ELISA antibody responses to the PA antigens will be evaluated using sera collected on day 0 and at every subsequent visit. The assay will be performed by following an assay developed by Conrad Quinn from the CDC for the AVA dose reduction study.

Toxin Neutralizing Antibody Assay

Anthrax lethal toxin neutralizing assay: PA, LF, and EF are the three major proteins of the two *B. anthracis* toxins, the lethal toxin and the edema toxin. Binding of PA to LF yields the *B. anthracis* lethal toxin, which is capable of intoxicating and lysing certain monocyte/macrophage cells and cell lines. This intoxication may be neutralized using antibodies or serum from immunized study volunteers. On the basis of available information on *B. anthracis* infection and anthrax toxin challenge, the immune response to the lethal toxin complex may be central to protection. Measurement of the neutralizing capacity of serum is therefore an appropriate marker of the human immune response for anthrax vaccines.

The purpose of this toxin neutralization assay (TNA) is to quantify the ability of the human humoral immune response to neutralize the lytic effects of anthrax lethal toxin *in vitro*. The assay will be used to evaluate the neutralizing capacity of serum from clinical trial participants.

The Study Specific Procedure was supplied by Dr. Conrad Quinn from the Centers for Disease Control, who is collaborating with WRAIR and DVC in conducting the TNA. Specifically, the TNA is designed to measure and quantify the functional ability of anti-PA antiserum to neutralize *B. anthracis* lethal toxin activity using an *in vitro* cytotoxicity assay. Cell viability is determined colorimetrically using a tetrazolium salt, 3-[4,5-dimethylthiazol-2-yl]-2,5-diphenyl-tetrazolium bromide (MTT), as the reporter or signal system. Serum-mediated neutralization of anthrax lethal toxin manifests as a suppression of lysis resulting in an increase in cell viability as determined using the MTT dye.

Sera from the immunized volunteers will be measured by following an assay developed by Conrad Quinn from the CDC for the AVA dose reduction study utilizing the J774A.1 cells.

5.6.1.2 Cellular Immune Responses to rPA:

Cellular immunogenicity will be assessed using the following assays:

1) Antigen-specific CD4 T Cell helper responses will be assessed using a Lymphocyte Proliferation Assay (LPA). Antigen will include whole rPA. Additional controls to include positive antigen control (Tetanus toxoid), negative antigen control and mitogens.
2) Antigen-specific CD8 T cell responses will be measured using a flow cytometric-based assay to measure cell-specific intracellular γ-IFN cytokine (ICC). Individual and/or pools of 15-20 mer synthetic peptides will be used to stimulate T cells for γ-IFN production.

Alternatively, or in addition, Antigen-specific CD8 T cell responses will be measured using an enzyme-linked immunosorbent SPOT assay measuring γ -interferon secretion (ELISPOT). Individual and/or pools of 15-20 mer synthetic peptides will be used to stimulate T cells for γ-IFN production. Cells will be collected for cellular immunogenicity at visits 1,4,7,8,9 and 10.

Lymphocyte Proliferation Assay (LPA). PBMC proliferative responses to antigens and mitogens will be measured by incubating 1 x 10^5 cells per well in 96-well U-bottom plates with serial antigen concentrations of rPA, control protein and tetanus toxoid and in separate plates with serial dilutions of mitogens PHA, PWM and ConA. After 3 days of incubation with the mitogens and 6 days with the antigens, cells are pulsed with 1 μCi/well of [^{3}H]-thymidine for 6 hr, harvested and counted. The data will be expressed as an LSI (Lymphocyte Stimulation Index) [LSI = (mean cpm of stimulated cells) / (mean cpm of unstimulated cells)] to define antigen specificity. Samples will be designated as positive if the LSI is greater than or equal to 5.

Gamma -interferon ELISPOT. This assay will be performed as described (36). Between 50,000 and 200,000 PBMC will be incubated with the following antigens and/or control reagents: 1) *E. coli* rPA protein and a control protein that has been made in the same manner as the rPA (LFn-p24); 2) PHA mitogen, and; 3) control pool of 23 MHC class I restricted peptides from CMV, EBV and influenza viruses. For each ELISPOT assay, 96-well mixed cellulose-ester plates will be coated overnight with an antibody specific for _-IFN. The plates will be washed, blocked and approximately 2x10^5 fresh PBMC will be added in duplicate or triplicate to each well and incubated with the antigens and mitogens indicated above. The plates will be incubated for 24h and the production of _-IFN by T cells will be detected by the addition of labeled antibody specific for _-IFN. After washing, the spots will be developed with an appropriate substrate, and the spots will be evaluated with an Automated Elispot Reader System by an independent scientist in a blinded fashion. The results will be expressed as the number of _-IFN secreting cells/10^6 PBMC (SFC/10^6 PBMC). A positive response will be defined as >3-fold above background and >20 SFC/10^6 PBMC.

Intracellular Cytokine Assay**.** Approximately 1x10^6 PBMC in 1 ml of complete RPMI media containing 10% fetal calf serum will be incubated with rPA, control protein or with a mixture of PMA and Iononmycin. The cultures will be incubated at 37°C in a 5% CO_2 incubator for one hour, followed by an additional 5 h incubation with 10 _g/ml of the secretion inhibitor Brefeldin-A. After washing, the cells will be surface stained with directly conjugated CD3, CD8, CD4 antibodies (or other surface markers of interest) for 30 minutes on ice. After another wash, cells will be fixed, permeabilized, stained with fluorochrome-conjugated _-IFN and CD69 antibodies for 30 minutes on ice and analyzed by flow cytometry.

5.6.1.3 Ancillary Studies:

Peripheral blood mononuclear cells will be harvested two weeks post the final injection to establish human monoclonal B cell lines with a view to produce and characterize monoclonal antibodies.

These monoclonal antibodies may help determine critical epitopes for vaccine induced protection and may be developed into products for treatment and diagnosis of Anthrax disease. This element of the study will be conducted based on availability of funds. Separate funding through the NIH has been sought by Dr. Susan Zolla-Pasner at NYU, an established expert in this field of endeavor.

Human specimens collected under this protocol and donated by the volunteer by written consent may be used to address other questions deemed appropriate by the HURC and HSRRB. The U.S. Army Medical Research and Materiel Command will be informed of and consulted regarding these ancillary studies. These results will be discussed by the aforementioned parties prior to public disclosure.

B-Cell antibody secreting cells (ASC). PBMC will also be collected to measure antibody secreting cells (ASC) specific for rPA. For each ELISPOT assay, 96-well mixed cellulose ester plates will be coated overnight either with an antibody specific for human IgG or with rPA. The plates will be washed, blocked and approximately $2x10^5$ fresh PBMC will be added in duplicate or triplicate to each well and incubated overnight and the secretion of total IgG or rPA-specific IgG will be detected by the addition of labeled antibody specific for human IgG. After washing, the spots will be developed with an appropriate substrate, and the spots will be evaluated with an Automated Elispot Reader System by an independent scientist in a blinded fashion. The results will be expressed as the number of antibody secreting cells/10^6 PBMC (SFC/10^6 PBMC) or per 10^6 IgG secreting cells. A positive response will be defined as >3-fold above background and >20 SFC/10^6 PBMC.

5.6.2 Specimen Handling and Processing:

- Venipuncture will be done according to institutional guidelines.
- Samples will be collected in the appropriate tubes as outlined in the attached table; substitution of tube types may be made as long as these are appropriate and do not interfere with the performance of the studies in question.
- All biological samples to be analyzed by the clinical laboratory will be collected and processed according to institutional guidelines.
- All samples sent to the WRAIR laboratory will be labeled with the following information
    - Bar Code of the volunteer's study number
    - Study volunteer's PIN number
    - Study visit number
    - Date
    - Study number (RV-147) and group #
    - Type of sample (serum, plasma, blood etc.)

- Samples that will be analyzed after freezing should be prepared, labeled, and stored at the appropriate temperature. Specimens that are shipped will be correctly prepared, labeled, and maintained at the correct temperature; all necessary shipping licenses/permits will be obtained.
- All safety laboratory samples drawn for the study will be sent to Quest Laboratory with the following information:

  - Study number (RV-147-xxx-DES), where xxx is the volunteer's study number and DES is the subject's initials
  - Subject's PIN
  - Date of birth
  - sex
  - Date drawn

5.6.2.1 Sample collection

Laboratory study	Type	Storage	Tube*	Amount
Hematology	blood	fresh	EDTA	3mL
Chemistry	serum	fresh	red	4mL
Troponin I and II	blood	fresh	green	3 mL
Serology	serum	fresh/frozen	red	4mL
rPA antibody	ser/plas	frozen	red	10 mL
Lymphocyte proliferation	cells	fresh/frozen	HEPARIN	10 mL/8 mL
γ-interferon ELISPOT	cells	fresh/frozen	HEPARIN	10 mL/8 mL

*Equivalent, appropriate substitutions to tube types are allowed

5.6.3 Safety Variables

5.6.3.1 Adverse Events:

An **adverse event** is any undesired, noxious or pathological change in a patient or study volunteer's as indicated by physical signs, symptoms, and/or laboratory changes that occurs following administration of one of the vaccines, whether or not considered vaccine related. This definition includes intercurrent illnesses or injuries, and exacerbation of pre-existing conditions.

Local reactions to immunization and selected systemic events will be solicited via study volunteer's interview and will be recorded on the post-injection reaction CRF form. Reactions occurring within 7 days post-immunization and all other adverse events will be recorded on the adverse event form. All adverse events occurring up to 30 days after the second injection will be collected. Subsequently during the 12-month study period only adverse events that are serious and/or necessitate a physician's visit or a prescribed medication will be collected. The study volunteer should be followed carefully until the condition is resolved and/or the cause is identified. Any medication or other therapeutic measure taken to relieve symptoms of the

medical problem must be recorded on the appropriate case report form page(s) in addition to the outcome of the adverse event.

Where a diagnosis is possible, it is preferable to report this rather than a series of terms relating to the diagnosis. When reporting a syndrome, indicate the associated signs and symptoms parenthetically following the syndrome rather than as separate events.

The **severity of events** reported on the adverse event form will be determined by the investigator, based on the Toxicity Grading Scale (Appendix 17.7) or if the event is not listed the event will be graded following these guidelines:

**Mild**	(Grade 1)	Transient or mild discomfort. No limitation in normal activities of daily living related to school or work.
**Moderate**	(Grade 2)	Some limitation in normal activities of daily living related to school or work.
**Severe**	(Grade 3)	Unable to perform normal activities of daily living related to school or work.
**Serious**	(Grade 4)	Life threatening.
**Death**	(Grade 5)	

The **relationship of an adverse event (AE) to immunization** will also be determined by the investigator, based on the following definitions:

Not Related:
- AE obviously explained by another cause; ***OR***
- The time of occurrence of AE is not reasonably related to vaccination.

Remotely Related:
- AE more likely explained by causes other than vaccination.

Possibly Related:
- Vaccine administration and AE occurrence reasonably related in time; ***AND***
- AE explained equally well by causes other than vaccination.

Probably Related:
- Vaccine administration and AE occurrence reasonably related in time; ***AND***
- AE more likely explained by vaccination than by other mechanisms.

Definitely Related:

- Vaccine administration and AE occurrence reasonably related in time; ***AND***
- Vaccination most likely explains the AE; ***AND***
- AE is consistent with pattern of vaccine-related events.

The **definition of a serious adverse event** (SAE) is as follows:

A **serious adverse event** is defined as any untoward medical occurrence that:

1. Results in death. **Report all deaths, whether or not suspected as being related to immunization.**
2. Is life threatening (i.e., the study volunteer was, in the opinion of the Investigator, at immediate risk of death from the event as it occurred).
3. Requires or prolongs inpatient hospitalization.
4. Results in persistent or significant disability/incapacity, (i.e., the event causes a substantial disruption of a person's ability to conduct normal life functions).
5. Results in congenital anomaly/birth defect.
6. Is an important and significant medical event that, based upon appropriate medical judgment, may jeopardize the patient or study volunteer's and may require medical or surgical intervention to prevent one of the other outcomes defining serious.

5.6.3.2 Reporting of Adverse Events

Adverse experiences that are both serious and unexpected will be immediately reported by telephone to the USAMRMC Office of Regulatory Compliance and Quality (301-619-2165) (non-duty hours call 301-619-2165 **and** send information by facsimile to 301-619-7803). A written report will follow the initial telephone call within 3 working days. Address the written report to the U.S. Army Medical Research and Materiel Command, ATTN: MCMR-RCQ, 504 Scott Street, Fort Detrick, Maryland 21702-5012.

In addition, i**mmediate reporting** to the following parties is required:
Office of Research Management, WRAIR, and the HSRRB immediately and to the Local Medical Monitor and the DVC (sponsor) Medical Monitor.

**Regulatory Affairs and Quality Control**	**DynPort Vaccine Company (Sponsor)**	**Local Medical Monitor**
Beryl Wessner, PharmD		CDR Sybil Tasker, MD, FCAP
Chief Regulatory Affairs	Kelly McKee, Jr., MD, MPH	Department of Infectious Disease
Henry M. Jackson Foundation	DynPort Vaccine Company	National Naval Medical Center
	60 Thomas Johnson Drive	
One Taft Ct., Suite 250	Frederick, MD 21702	Bethesda, MD
Rockville, MD 20850	Phone: 1-866-DYNPORT (396-	Phone: (301)-252-5077

	7678)	
Phone: (301) 251- 5037	Phone Direct: (301)-607-5061	
	Cell Phone: (443)-812-2596	Cell Phone (202)-361-3923
		(24-hour availability)
FAX: (301) 294-1898	FAX: (301)-607-5098	FAX: (301)-762-4177
Email: bwessner@hivresearch.org	Email: mckeek@dynport.com	Email:

Serious and unexpected adverse events which are unlikely to be related to the test article and are NOT within the expected life experience of the general population will be reported to the HSRRB immediately by phone to the Deputy Chief of Staff for Regulatory Compliance and Quality, 301-619-2165. The sponsor and medical monitor will be similarly notified.
A written report to follow the initial telephone call should be provided within 3 days. Follow-up information should be provided when available. All serious adverse event reports should include the study title and HSRRB Log No, description of event, subject status, any actions taken in response to the event and the name of the individual making the report and their relationship to the study. Telephonic reporting must be followed by a written report within 24 hours to the following Local Medical Monitor.

All other adverse events including serious and unexpected adverse events which are unlikely to be related to the test article and are within the expected life experience of the general population will be listed at regular intervals, reviewed by the local medical monitor and reported to the HSRRB, the sponsor medical monitor, and the HURC will be similarly notified. A written report by the Principal Investigator and a written concurrence and/or commentary by the Local Medical Monitor to include the study title and HSRRB Log No, description of events, subject status, any actions taken in response to the event and their relationship to the study.

Further immunizations should not be given to any study volunteers who have experienced a serious adverse event, whether related to vaccine or not. **Decisions to continue immunizations should be made only after consultation and written consent of the medical monitors.**

5.6.3.3 Monitoring of Post-Immunization Events:

Selected local and systemic adverse events are routinely monitored in vaccine clinical trials as indicators of vaccine reactogenicity. It is recognized that each of these events, and particularly those of a systemic nature, may under some circumstances be unrelated to study vaccines. However, as a matter of convenience and in accordance with common clinical practice, all such events occurring within 7 days of immunization (including the day of immunization) are herein termed "*post-immunization reactions*".

5.6.3.4 Post-immunization Reactions Occurring Immediately After Immunization

Following each vaccine administration, study volunteers will be observed in the clinic for 30 minutes. Study personnel will then evaluate the study volunteer for any signs or symptoms of

local or systemic reactions. These will be noted in the case report form. Possible local reactions that should be recorded include erythema and induration (measured using the ruler indicated on the diary card that will be provided to the study volunteer's), pain, tenderness, and warmth at the injection site. Systemic symptoms to be noted on the diary include fever (>38^{0}C), fatigue, headache, myalgia, arthralgia, chills, anorexia, nausea, vomiting, shortness of breath, chest pain, and rash. For each immunization, oral temperature will be recorded prior to immunization and 30 minutes after immunization.

5.6.3.5 Telephone Contact after each Immunization (TC)

The study nurse or designee will contact all volunteers by telephone on the day following the vaccination to determine if any reactions have occurred. Volunteers will be queried using a predetermined review of systems/reactogenicity worksheet. If the adverse effects experienced by the volunteer are deemed "moderate or severe", he/she will be asked to return to clinic for evaluation by the investigator and safety labs will be obtained. Additional contacts will be arranged as needed.

5.6.3.6 Safety Evaluation after each Immunization

All study volunteers will return to the clinic within 48 to 72 hours post-immunization for:

- Interim History and Physical
- AE elicitation and review of the diary card.
- Collect samples for safety labs: UA and CBC, complete serum chemistries

5.6.3.7 Local Reactions Within 7 Days Post-immunization (Study Days 0 to 6)

The study volunteer will be asked to note occurrences of erythema and induration (measured using the ruler included on the diary card), and pain, tenderness, or warmth at the injection sites **daily** for 7 days. These occurrences should be recorded on the diary card provided to serve as a reminder to the study volunteer for the next clinic visit. The study site will keep a copy of the diary card.

5.6.3.8 Systemic Reactions Within 7 Days Post-immunization (Study Days 0 to 6)

The study volunteer will also be asked to note occurrences of fever (>38^{0}C), fatigue, headache, myalgia, arthralgia, chills, nausea, vomiting, anorexia, and rashes **daily** for 7 days and record these occurrences on the diary card mentioned previously. Oral temperature should be recorded by the study volunteer 6 hours after immunization, and then record evening temperatures daily for the next 7 days (for a total of 7 days of observation). If a medication is taken for treatment of fever, the temperature should be retaken and recorded 4 hours after the antipyretic dose to see if further treatment is necessary.

5.6.3.9 Post-immunization Contact

The study nurse or designee will contact the study volunteer by telephone on the day following the vaccination to determine if any reactions have occurred. Volunteers will be queried using a

predetermined review of systems/reactogenicity worksheet. If the adverse effects experienced by the volunteer are deemed "moderate or severe", he/she will be asked to return to clinic for evaluation by the investigator and safety labs will be obtained. Additional contacts will be arranged as needed.

5.6.3.10 Instructions to Study Volunteers Regarding Unusual or Severe Signs or Symptoms

The study volunteer will be instructed to call the specified study personnel immediately if any unusual or severe sign or symptom appears after immunization. The study volunteers should be seen in the clinic at the time of maximal symptoms, if possible, and will be followed up clinically until resolution of symptoms.

5.7 Statistical Methods and Determination of Sample Size

5.7.1 Statistical Plan: Design & Analysis

This is not a study designed to resolve a hypothesis and assessment of power is not provided. Rather, this study is designed to provide preliminary data, which can inform the design of subsequent studies designed to definitively address a hypothesis.

Data analysis will be performed at WRAIR. Any data analyses carried out independently by investigators should be submitted to DVC before publication or presentation.

The primary safety measurements include data from the clinical laboratory tests, observed immunization reactions (local and systemic) and adverse events. The primary immunogenicity data include serum antibody responses to vaccine antigens as evaluated by anti-PA IgG ELISA and TNA. Cellular responses will be assayed by LPA, ICC, and _-IFN ELISPOT.

All study volunteer's receiving at least one injection of vaccine will be included in the safety and tolerability analyses. The study volunteers will be excluded from immunogenicity analyses at each time point if their blood draw occurs more than 7 days outside the acceptable visit window. All data exclusions, including premature terminations, will be detailed and tabulated according to vaccine group and site. Data listings will include all study volunteers.

Demographic data obtained during the baseline visit will be listed for each study volunteer. Summary statistics will be tabulated for each vaccine group. Study volunteers will be assessed for comparability at baseline. Descriptive statistics will be presented for continuous variables (age, height, weight). Frequency counts and percents will be presented for categorical variables (age, sex and race).

Non-parametric tests will be used to evaluate Ab titers. ELISPOT and LPA responses will be compared using the chi-squared analysis, with the presence or absence of cumulative ELISPOT responses being positive or negative. Statistically significant results will be annotated by their degree (*P<0.05;**P<0.01;***P<0.001). Statistical significance will be considered if the p-value is less than 0.05.

5.7.2 Safety

All study volunteers receiving an injection of vaccine will be included in the safety and tolerability analyses.

5.7.2.1 Post-immunization Reactions (Local and Systemic)

The maximal severity of local and systemic post-immunization reactions occurring in the seven days following each immunization and "any immunization" will be tabulated by group. Local reactions tabulated will include: injection site pain, erythema, induration, and temperature. Systemic reactions reported within seven days post-immunization will include: chills, nausea, malaise, myalgia, arthralgia, headache, rash and fever (oral temperature greater than or equal to 38ºC (100.4ºF). Additionally, pain on injection will be noted. Frequencies and percentages of study volunteers experiencing each reaction will be presented for each symptom severity. If a reaction occurs more than once for a study volunteer's, the reaction will be classified according to the highest occurring severity and closest vaccine relationship.

Summary tables showing the occurrence of any local or systemic reactions overall and at each time point will also be presented.

5.7.2.2 Other Adverse Events

The original terms used by investigators on the CRF will be translated to MedDRA terms and grouped into body system and presented as frequency tables. For each and any immunization, the number and percent of study volunteers with adverse events will by tabulated, by group. When adverse events occur more than once, the maximal severity and relationship to vaccine will be counted. Three summaries will be generated: (1) serious adverse events (SAEs); (2) adverse events that are definitely, probably or possibly related to vaccination, and (3) adverse events that are remotely or not related to vaccination. Data listings of all adverse events will be provided by study volunteers, as will subset listings summarizing study volunteers withdrawn from the study because of an adverse event.

5.7.2.3 Clinical Laboratory

Listings and descriptive statistics will be generated for all laboratory parameters that are abnormal, by group. Data listings will be provided by study volunteer's summarizing all abnormal values, by parameter (e.g., WBC count, hemoglobin, platelet count, ALT, creatinine, UA).

5.7.3 Immunogenicity

Humoral Assessment: For each group, least squares geometric mean titer (GMTs), associated 95% confidence intervals, and median, minimal and maximal titers to rPA and native PA will be calculated based on results from the anti-PA IgG ELISA. The toxin-neutralization assay will be used to determine the reciprocal of the dilution of a serum sample that effects a 50% neutralization of lethal toxin. Neutralization is based on cell viability following incubation with serum and toxin using a colorimetric assay based on dye exclusion. The Study Specific

Procedures for these assays are being provided by Dr. Conrad Quinn from the Centers for Disease Control who developed the assays for an AVA dose reduction study.

Cellular Assessment: Lymphocyte proliferation will be described as a stimulation index (LSI = CPM/Medium Control). For each group (and site), geometric mean LSI, median minimal and maximal LSI, as associated 95% confidence intervals will be calculated. ELISPOT data will be analyzed in bulk and after CD4 and CD8 cell depletion. The number of ELISPOT's for each pool of peptide will be enumerated and the geometric mean, median, minimal and maximal ELISPOT response, and associated 95% confidence intervals will be calculated.

6.0 COMPENSATION, INSURANCE, INDEMNITY

6.1 Information on Compensation, Insurance and Indemnity

If the study volunteer is a DEERS eligible Federal Government employee participating in this study outside normal duty hours, the study volunteer will receive $100.00 after each blood draw, including the last visit, for a total of $1300 as compensation for time, transportation and inconvenience.

If the study volunteer is not a Department of Defense beneficiary, he/she will receive $100.00 after each blood draw, including the last visit, for a total of $1300 as compensation for time, transportation and inconvenience.

7.0 VACCINE ACCOUNTABILITY

The investigator or designee must maintain accurate records of dates and quantities, and lots of product(s) received, to whom dispensed (study volunteer's-by-study volunteer's accounting), and accounts of any product accidentally or deliberately destroyed. The investigator must retain all unused or expired product until the study monitor has confirmed accountability data.

At the conclusion of vaccine administration, all vaccine supplies (including used and unused vials) will be disposed of according to the procedure agreed upon. An overall summary of all vaccine supplies received, used and returned must be prepared at the conclusion of the study.

8.0 LABORATORY REQUIREMENTS

Clinical laboratory evaluations for safety will be performed at each time point during the trial.

9.0 CASE REPORT FORMS

Case report forms (CRF) will be provided for each study volunteer's. Correction to data on CRFs may be made only by putting a single line through the incorrect data and writing the correct values, allowing the original text to remain legible. Each correction should be initialed and dated by the person making the change. If corrections are made after review and signature by the investigator, he/she must be made aware of the changes and document this awareness.

It is the policy of WRAIR and DVC that the study data must be verifiable to the source data, which necessitates access to all original recordings, laboratory reports, and study volunteers' records. The investigator must therefore agree to allow access to study volunteers' records, and source data must be made available for all study data.

10.0 STUDY MONITORING AND COMPLIANCE

All aspects of the study will be carefully monitored by the sponsor, authorized representatives of the sponsor, or representatives of the DoD with respect to current good clinical practices and standard operating procedures for compliance with applicable government regulations. These individuals will have access, both during the trial and after trial completion, to review and audit all records necessary to ensure integrity of the data, and will periodically review progress of the study with the principal investigator.

Protocol violations (deviations from protocol-specified performance) may be identified by any member of the study team, monitors or DVC monitors. Dr. Beryl Wessner, Chief Regulatory Affairs will promptly report these deviations to the HMJF, HSRRB, DVC, and HURC.

Every attempt must be made to follow the protocol and to obtain and record all data requested for each study volunteer's at the specified times. However, ethical reasons may warrant the failure to obtain and record certain data, or to record data at the times specified. If this becomes necessary, the reasons for such deviation must be clearly documented on the case report form.

11.0 INFORMED CONSENT

A written informed consent document in compliance with 21 CFR 50 will be provided to the Investigator by DVC. Any changes made by an Investigator to this template must be approved by the WRAIR Principal Investigator prior to submission to his/her IRB.

Prior to enrollment in the study each volunteer will be informed in detail about the vaccination, the procedures to be performed, the nature of the clinical investigation and the risks and discomforts to be expected. The basic elements of informed consent as specified by the FDA (21 CFR 50.25) will be followed. Written consent will be obtained from each volunteer using the HURC- and HSRRB-approved consent form. This is in accordance with Title 10, Section 980 of the United States Code (10 USC 980), which requires that "funds appropriated to the Department of Defense may not be used for research involving a human being as an experimental study volunteer's unless:

1) The informed consent of the study volunteer is obtained in advance; or
2) In the case of research intended to be beneficial to the study volunteer's, the informed consent may be obtained from a legal representative of the study volunteer's.

The person explaining the consent and a witness will verify consent. Each volunteer will be given a copy of the consent form. Volunteers will be told that they are free to withdraw their consent and discontinue participation at any time without prejudice or loss of benefits to which the volunteer is otherwise entitled.

Successful completion of the "Test of Understanding" (Section 17.2) defined as 90% correct with three opportunities to take the test. Errors will be reviewed with the volunteer after each test.

12.0 RETENTION OF RECORDS

Because data from clinical trials sponsored by WRAIR may be used to support regulatory filings in several countries throughout the world, the policy concerning record retention reflects the most stringent current guidelines (those of the Committee for Proprietary Medicinal Products, or CPMP, in Europe). To comply with the CPMP guidelines, WRAIR requests that the investigator arrange for the retention of case report forms, source records, and other supporting documentation for a minimum of 15 years.

13.0 USE OF INFORMATION AND PUBLICATION

It is understood by the investigator that the information generated in this study will be used by DVC in connection with the development of the product and therefore may be disclosed to government agencies in various countries. To allow for the use of information derived from the study, it is understood that the investigator is obliged to provide the sponsor with complete test results, all study data, and access to all study records. DVC recognizes the importance of communicating medical study data and therefore encourages their publication in reputable scientific journals and at seminars or conferences. Any results of medical investigations and or publication/lecture/manuscripts based thereon, shall be exchanged and discussed by the investigator, DVC representative(s) and the U.S. Army Medical Research and Materiel Command 60 days prior to submission for publication or presentation.

Results from investigations shall not be made available to any third party by the investigating team outside the publication procedure as outlined previously. WRAIR will not quote from publications by investigators in its scientific information and/or promotional material without full acknowledgment of the source (i.e., author and reference).

14.0 PROTOCOL AMENDMENTS

Amendments to the protocol will be made only after consultation and agreement between sponsor and principal investigator. **"All protocol modifications (including but not limited to changes in the principal investigator, inclusion/exclusion criteria, number of subjects to be enrolled at study site, or procedures) must be submitted as a written amendment for local IRB/HURC approval before implementation of the changes, with copies of the amendment and the local IRB/HURC approval furnished to the HSRRB for their records. All amendments that increase the level of risk to the study participants must receive HSRRB approval in addition to local approval prior to implementation of the changes".** A list of proposed modifications or amendments to the protocol and an explanation of the need for these modifications will be submitted, along with a revised protocol incorporating the modifications. The only exception occurs when the investigator considers that a study volunteer's safety is

compromised without immediate action. In these circumstances, immediate approval of the chairman of the WRAIR IRB (HURC) must be sought, and the investigator should inform the sponsor and the WRAIR IRB (HURC) and the HSRRB within 5 working days after the emergency occurred.

15.0 REFERENCES

1. Hanna P. Anthrax pathogenesis and host response. Curr. Top. Microbiol. Immunol. (1998); 225:13-35.

2. Friedlander AM. Anthrax. In: "Medical Aspects of Chemical and Biological Warfare", Editors: Sidell, Takafuji and Franz. Published by Office of the Surgeon General of the Army, (1997); 467-478.

3. Dixon TC, Meselson M, Guillemin J, Hanna PC. Anthrax. NEJM (1999) 341:815- 826.

4. Kaufman AF. Observations on the occurrence of anthrax as related to soil type and rainfall. Salisbury Med Bull Suppl.(1990); 68: 16-17.

5. De Vos V. The ecology of anthrax in the Kruger National Park, South Africa. Salisbury Med. Bull. Suppl. (1990); 68: 19-23

6. Christopher GW, Cieslak TJ, Pavlin JA, Eitzen EM Jr. Biological Warfare: A historical perspective. JAMA (1997); 278: 412-417.

7. Inglesby T.V., et al. Anthrax as a biological weapon: medical and public health management. Working group on civilian biodefense. J. Am. Med. Assoc. (1999); 281: 1735-1745.

8. Friedlander AM, Welkos SL, Pitt MLM, et al. Postexposure prophylaxis against experimental inhalation anthrax. J. Infect. Dis. (1993); 167:1239-1243.

9. Messelson M, Guillemin J, Hugh-Jones M, et al. The Sverdlovsk anthrax outbreak of 1979. Science (1994);266:1202-1208.

10. Brachman PS, Friedlander AM. Anthrax. In: Plotkin SA, Orenstein WA. (eds.), Vaccines (3rd Edition). W.B. Saunders, Philadelphia, PA. p. 629-637, 1999.

11. Leppla SH. The anthrax toxin complex. Sourcebook of bacterial protein toxins. Academic Press, (1991); 277-302.

12. Ivins BE, Welkos SL. Cloning and expression of the *Bacillus anthracis* protective antigen in Bacillus subtitis. Infect. Immun. (1986); 54: 537-2.

13. Uchida I, Hashimoto K, Terakato N. Virulence and immunogenicity in experimental animals of Bacillus anthracis strains harboring or lacking 110 MDa and 60 MDa plasmids. J. Gen. Microbiol. (1986); 132: 557-9.

14. Petosa C, Collier RJ, Klimpel KR, Leppla SH, Liddington RC. Crystal structure of the anthrax toxin protective antigen. Nature (1997); 385: 833-838.

PROPRIETARY

15. Miller CJ, Elliott JL, Collier RJ. Anthrax protective antigen: prepore-to-pore conversion. Biochemistry (1999); 39(32):10432-10441.

16. Leppla SH. Anthrax toxin edema factor: A bacterial adenylate cyclase that increases cyclic AMP concentration of eukaryotic cells. Proc. Natl. Acad. Sci. USA (1982); 79: 3162-6.

17. Klimpel KR, Arora N, Leppla SH. Anthrax toxin lethal factor contains a zinc metalloprotease consensus sequence which is required for lethal toxin activity. Mol. Micro. (1994); 13: 1093-100.

18. Duesbery NS, Webb CP, Leppla SH, Gordon VM, Klimpel KR, Copeland TD, Ahn AG, Oskarsson MK, Fukasawa K, Paull KD, Vander Woude GF. Proteolytic inactivation of MAP-Kinase-kinase by anthrax lethal factor. Science (1998); 280: 734-7.

19. Bradley KA, Mogridge J, Mourez M, Collier RJ, and Young JA. Identification of the cellular receptor for anthrax toxin. Nature. (2001); 414(6860):225-229.

20. Turnbull PCB. Anthrax vaccines: past, present and future. Vaccine (1991); 9:533-539.

21. Brachman PS, Gold H, Plotkin SA, Fekety FR, Werrin M, Ingraham NR. Field evaluation of a human anthrax vaccine. Am. J. Public Health (1962); 52:632-445.

22. Wright G, Green TW, Kanode RG Jr. Studies on immunity in anthrax. V. Immunizing activity of alum-precipitated protective antigen. J. Immunol. (1954); 73:387-391.

23. Friedlander AM, Pittman PR, Parker GW. Anthrax vaccine: evidence for safety and efficacy against inhalation anthrax. JAMA (1999); 282(22):2104-2106.

24. Johnson-Winegar, A. Comparison of enzyme-linked immunosorbant and hemagglutination assays for determining anthrax antibodies. J. Clin. Microbiol. (1984); 20:357-361.

25. Buchanan TM, Feeley JC, Hayes PS, Brachman PS. Anthrax indirect microhemagglitunation test. J. Immunol. (1971); 107:1631-1636.

26. Reuveny S, White MD, Adar YY, Kafri Y, Altboum Z, Gozes Y, Kobiler D, Shafferman A, Velan B. Search for correlates of protective immunity conferred by anthrax vaccine. Infect. Immun. (2001);69 (5):2888-2893.

27. Pitt MLM, Little SF, Ivins BE, Fellows P, Barth J, Hewetson J, Gibbs P, Dertzbaugh M, Friedlander AM. In vitro correlate of immunity in a rabbit model of inhalation anthrax. Vaccine (2001); 19:4768-4773.

28. Ivins BE, Pitt ML, Fellows PF, Farchaus JW, Benner GE, Waag DM, Little SF, Anderson GW Jr, Gibbs PH, Friedlander AM. Comparative efficacy of experimental anthrax vaccine candidates against inhalation anthrax in rhesus macaques. Vaccine 16(11-12):1141-1148, 1998.

29. Duesbery NS, Webb CP, Leppla SH, Gordon VM, Klimpel KR, Copeland TD, Ahn AG, Oskarsson MK, Fukasawa K, Paull KD, Vander Woude GF. Proteolytic inactivation of MAP-Kinase-kinase by anthrax lethal factor. Science 1998; 280: 734-7.

30. Milne JC, Blanke SR, Hanna PC, Collier RJ. Protective antigen-binding domain of Anthrax lethal factor mediates translocation of a heterologous protein fused to its amino- or carboxyl-terminus. Mol Microbiol 1995; 15: 661-6.

31. Arora N, Williamson LC, Leppla SH, Halpern JL. Cytotoxic effects of a chimeric protein consisting of tetanus toxin light chain and anhtrax toxin lethal factor in non-neuronal cells. J Biol Chem 1994; 269: 26165-7.

32. Ballard JD, Collier RJ, Starnbach MN. Anthrax toxin-mediated delivery of a cytotoxic T-cell epitope in vivo. Proc Natl Acad Sci USA 1996; 93: 12531-4.

33. Goletz TJ, Klimpel KR, Arora N, Leppla SH, Keith JM, Berzofsky JA. Targeting HIV proteins to the major histocomatibility complex class I processing pathway with a novel gp120-anthrax toxin fusion protein. Proc Natl Acad Sci USA 1997; 94: 12059-64.

34. Ballard JD, Doling AM, Beauregard K, Collier RJ, Starnbach MN. Anthrax toxin-mediated delivery in vivo and *in vitro* of a cytotoxic T-lymphocyte epitope from ovalbumin. Infect Immun 1998; 66: 615-9.

35. Gupta RK, Relyveld EH, Lindblad EB, Bizzini B, Ben-Efraim S, Gupta CK. Adjuvants: a balance between toxicity and adjuvanticity. *Vaccine* (1993) 11: 293-306.

36. Currier J, Kuta E, Turk E, Earhart LB, Loomis-Price L, Janetzki S, Ferrari G, Birx, DL and Cox, JH. 2002. A panel of MHC class I restricted viral peptides for use as a quality control for vaccine trial ELISPOT assays. J. Immunol. Meth. 260: 157-172.

16.0 SIGNATURE PAGE

Protocol Entitled: **A Phase 1 Study of Safety and Immunogenicity of *E. coli*-Derived Recombinant Protective Antigen (rPA), a New Anthrax Vaccine Administered by the Intramuscular (IM) Route in Healthy Adults**

Approved by:

____

Merlin Robb, MD **Date**
Principal Investigator
Chief, HIV Vaccine Development Program, WRAIR

____

Deborah Birx, COL, MC **Date**
Director, Division of Retrovirology, WRAIR

I have read the foregoing protocol and agree to conduct the study as outlined. In addition, I agree to conduct the study in compliance with all applicable regulations and guidelines as stated in the protocol and other information supplied to me.

____

Principal Investigator's Signature Typed Name Date

On behalf of DVC, I confirm that the sponsor will comply with all obligations as detailed in all applicable regulations and guidelines. In addition, I will ensure that the investigator is informed of all relevant information that becomes available during the conduct of this study.

____

Medical Monitor's Signature Typed Name Date

17.0 APPENDICES

17.1 STATEMENT OF OBLIGATIONS OF SPONSOR/MONITOR, MEDICAL MONITOR, AND CLINICAL INVESTIGATOR

Sponsor/Monitor
The sponsor or his/her designated representative, the monitor, will:

1. Conduct a pre-investigation visit to:
   a. Establish the acceptability of the facility and record this in a written report (memorandum or form).
   b. Discuss the proposed clinical trial with the investigator, supply the Case Report Form, the Investigator's Brochure, and the draft Protocol for review and approval.
   c. Discuss with the investigator FDA requirements with respect to Informed Consent, Institutional Review Board (IRB, HURC) approval of the trial, the Protocol, including Protocol amendments and Informed Consent changes.

2. Conduct periodic on-site visits to:
   a. Assure adherence to the Protocol.
   b. Review Case Report Forms and medical records for accuracy and completeness of information.
   c. Examine pharmacy records for documentation of: quantity and date of receipt of investigational vaccine, dispensation and accountability data for vaccine administration to each study volunteer's, loss of materials, contamination, etc, and unused supplies.
   d. Record, report (summarize) observations on the progress of the trial and continued acceptability of the facilities; prepare an on-site visit report.
   e. Review investigator files for required documents, i.e., protocols, protocol amendments, IRB approvals (Protocols, amendments, Informed Consent, etc), IRB charter and membership, communications to and from the IRB and the monitor.

Clinical Investigator

1. Institutional Review Board (IRB)
   The investigator must assure the monitor that the IRB:
   a. Meets FDA regulations as defined in 21 CFR Part 56.
   b. Has authority delegated by the parent institution and found in IRB by-laws, operation guidelines or charter to approve, or disapprove, clinical trials and Protocols including Informed Consent and other documents (protocol amendments, information to be supplied to study volunteers concerning Informed Consent, etc.).

c. Complies with proper personnel makeup of IRB.
d. Convenes meetings using acceptable rules of order for making decisions, recording such decisions and implementing them.
e. Files contain (a) documentation of its decisions such as are found in IRB minutes and correspondence, (b) written guidelines or by-laws governing IRB functions, (c) protocols, (d) protocol information to be supplied to the study volunteer's, (e) correspondence between IRB and investigator (consent changes, protocol amendments, etc.).

2. Informed Consent of Human Study volunteers.
The investigator must assure monitor that the Informed Consent for a study volunteer's:
a. Meets FDA regulations as defined in 21 CFR Part 50 Informed Consent.
b. Has been approved by the IRB, including, when required, information to be given to the study volunteer regarding the trial he/she is enrolled in.
(1) Informed Consent includes the basic elements and any additional elements necessary.
(2) The study volunteer and a study site representative sign the form, and the study volunteer is given a copy.

3. Storage and Dispensing of Vaccine Supplies.
The investigator (or pharmacist) must assure (demonstrate to) the monitor that:
a. Adequate and accurate written records show receipt and disposition of all vaccine supplies, including dates, serial or lot number, quantities received, each quantity dispensed, administered or used with identification of each study volunteer's.
b. Purpose and reasons are given in written records for vaccine disposal, i.e., the amount contaminated, broken, or lost, etc., and quantity returned to the sponsor.

4. Case Report Forms.
The investigator must assure the monitor that:
a. Case Report Forms, when completed, accurately reflect the medical records on each study volunteer's or patient.
b. Case Report Forms and medical records will be accessible to the monitor or FDA inspectors' on-site visits.

5. Files and Records.
The investigator must assure the quality, integrity, and content of his/her files that will be inspected by the monitor and FDA inspectors. The files must contain, as a minimum:
a. Correspondence to and from IRB and the monitor.
b. Documents that include:
(1) IRB-approved protocols.
(2) IRB-approved protocol amendments.

(3) IRB-approved informed consent and information supplied to the study volunteer or study volunteer's.
(4) IRB charter, membership, and their qualifications.

c. Documents and records must be retained for a minimum of 15 years.

b. Clinical supplies:
(1) Record of receipt, date and quantity, batch or lot number.
(2) Disposition dates and quantity administered to each study volunteer's.
(3) Inventory records.

<u>Local Medical Monitor</u>

1. A Local Medical Monitor will be assigned to the study.

2. The medical monitor should have adequate training, experience and appropriate credentials to provide medical care to research study volunteers for conditions that may arise during the conduct of the study.

3. Duties:
   a. Monitor the study volunteers during the conduct of the study.
   b. Review all serious and unexpected adverse events (per ICH definitions) associated with the protocol and provide an unbiased written report of the event within 10 calendar days of the initial report.
   c. Provide a written comment on the outcomes of the adverse event (AE) and relationship of the AE to the test article.
   e. Indicate whether he/she concurs with the details of the AE or SAE reports provided by the study investigator.

17.2 BRIEFING DOCUMENT FOR STUDY VOLUNTEER'S ENROLLMENT PROCEDURE

PROPRIETARY

17.3 DRAFT INFORMED CONSENT FORM

17.4 TEST OF UNDERSTANDING AND COMMITMENT

1. Anthrax is a disease in humans and cattle. T or F
2. The vaccine I am going to receive has only a single component of the Anthrax bacteria called "protective antigen". T or F
3. I may take other experimental products while I am participating in this study. T or F
4. I may become pregnant or father a child during the course of this study. T or F
5. This experimental vaccine study is designed to determine if it is safe and produces immune response to "Protective Antigen". T or F
6. I will come to clinic for 13 visits including the visit today and will be contacted by telephone the following day after each immunization. T or F
7. If I have a reaction or problem while receiving vaccine I can contact the study team through the clinic or a pager system. T or F
8. I will receive general medical care at the vaccine clinic while I am in the study T or F
9. The experimental vaccine I am receiving is known to protect against anthrax. T or F
10. I may withdraw from the study at any time if I choose or my participation can be halted if the research team determines it is in by best interest. T or F

17.5 PREGNANCY REPORTING

For study volunteers who become pregnant after receiving any immunization: The study volunteer may continue on the study with all scheduled visits, but no further immunizations will be given. For all study volunteers who become pregnant while on the trial (including those mentioned above), a Pregnancy Follow-up CRF should be completed as soon as possible. The site should maintain contact with pregnant study volunteers to obtain pregnancy outcome information for the Pregnancy Follow-up CRF.

Specific information to be collected includes the following:

1. Pregnancy Report:

   Date of last menstrual period, date pregnancy confirmed, estimated date of confinement, history of children born with congenital abnormalities, and history of spontaneous abortions.

2. Pregnancy Follow-up:

   a) Date of delivery or termination, outcome of pregnancy (i.e., spontaneous abortion, therapeutic abortion, ectopic pregnancy, stillborn delivery, live born delivery) and the presence or absence of congenital abnormalities in the infant.

   b) Abnormal Pregnancy Outcome-mother (To be completed in the event of a delivery of an infant with congenital abnormalities.)

   c) Complications during pregnancy, labor and delivery; information regarding prenatal care, infections, illnesses, and medications taken during pregnancy; use of drugs, alcohol, or other conditions during the course of the pregnancy.

3. Abnormal Pregnancy Outcome-infant (to be completed in the event of a delivery of an infant with congenital abnormalities):

The sex, weight, estimated gestational age, and description of abnormalities present in the infant.

PROPRIETARY

17.6 MEDICAL MONITORS

Local Medical Monitor:

CDR Sybil Tasker, MD, FCAP
Department of Infectious Disease
National Naval Medical Center
Bethesda, MD
Phone: (301)-252-5077
Cell Phone (202)-361-3923
(24-hour availability)
FAX: (301)-762-4177

Sponsor Medical Monitor:

DynPort Vaccine Company

Kelly McKee, Jr., MD, MPH
DynPort Vaccine Company
64 Thomas Johnson Drive
Frederick, MD 21702
Phone: 1-866-DYNPORT
Phone Direct: 301-607-5061
Mobile Phone: 443-812-2596
FAX: 301-607-5098
Email: mckeek@dynport.com

PROPRIETARY

17.7 TOXICITY GRADING SCALE

Table 10 Toxicity Grading Scale for Adverse Events

**HEMATOLOGY**				
**PARAMETER**	**GRADE 1**<br>***MILD***	**GRADE 2**<br>***MODERATE***	**GRADE 3**<br>***SEVERE***	**GRADE 4**<br>***POTENTIALLY LIFE THREATENING***
Hemoglobin	9.5 g/dL - 10.5 g/dL_8.0 g/dL - 9.4 g/dL_7.9 g/dL - 6.5 g/dL_<6.5 g/dL__Absolute Neutrophil Count_1000 - 1500/mm^3_750 - 999/mm^3_500 - 749/mm^3_<500/mm^3__WBC_>13,000_>15,000_>20,000_>30,000 or <1,000__Percent Polys + Bands_>80%_90%_≥95%________Platelets_ 100,000 - 120,000/mm^3 400,000 – 500K	75,000 - 99,999/mm^3 > 500K	50,000 - 74,999/mm^3 > 1M	20,000 - <50,000/mm^3
CD4 Counts<br>Uninfected	300 - 400/mm^3<br><300 or <20%	300/mm^3<br><200 or <18%	200/mm^3<br><100 or 15%	<100/mm^3<br><50 or <12%
Fibrinogen	100 - 200 mg/dL OR 400 - 600 mg/dL	<100 mg/dL OR >600 mg/dL	<50 mg/dL or associated with gross bleeding OR associated with disseminated coagulation	-----
Prothrombin Time (PT)	>1.0 - 1.24 x ULN	>1.25 - 1.49 x ULN	>1.5 - 3.0 x ULN	>3.0 x ULN
PTT	>1.0 - 1.66 x ULN	>1.66 - 2.33 x ULN	>2.33 - 3.0 x ULN	>3.0 x ULN
**CHEMISTRY**				
**PARAMETER**	**GRADE 1**<br>***MILD***	**GRADE 2**<br>***MODERATE***	**GRADE 3**<br>***SEVERE***	**GRADE 4**<br>***POTENTIALLY LIFE THREATENING***
CPK	≥4 ULN	≥6 ULN	≥10 ULN	≥20 ULN
Creatinine	>1.0 - 1.5 x ULN	>1.5 - 1.9 x ULN	>2.0 - 6.0 x ULN	>6.0 x ULN

SODIUM<br>Hyponatremia<br>Hypernatremia	130 - 135 meq/L<br>146 - 150 meq/L	123 - 129 meq/L<br>151 - 157 meq/L	116 - 122 meq/L<br>158 - 165 meq/L	<116 meq/L<br>>165 meq/L
POTASSIUM<br>Hyperkalemia<br>Hypokalemia	5.0-5.5 meq/L<br>3.2 - 3.4 meq/L	5.6 - 6.0 meq/L<br>3.0 - 3.1 meq/L	6.1 - 6.5 meq/L<br>2.5 - 2.9 meq/L	>6.6 meq/L<br><2.5 meq/L
PHOSPHATE<br>Hypophosphatemia	2.0 - 2.4 mg/dL	1.5 - 1.9 mg/dL	1.0 - 1.4 mg/dL	<1.0 mg/dL
CALCIUM (corrected for albumin)<br>Hypocalcemia<br>Hypercalcemia	7.8 - 8.4 mg/dL<br>10.6 - 11.5 mg/dL	7.0 - 7.7 mg/dL<br>11.6 - 12.5 mg/dL	6.1 - 6.9 mg/dL<br>12.6 - 13.5 mg/dL	<6.1 mg/dL<br>>13.5 mg/dL
MAGNESIUM<br>Hypomagnesemia	1.2 - 1.4 meq/L	0.9 - 1.1 meq/L	0.6 - 0.8 meq/L	<0.6 meq/L
BILIRUBIN<br>Hyperbilirubinema	>1.0 - 1.5 x ULN	>1.5 - 2.5 x ULN	>2.5 - 5 x ULN	>5 x ULN
GLUCOSE<br>Hypoglycemia<br>Hyperglycemia (nonfasting and no prior diabetes)	55 - 64 mg/dL<br>116 - 160 mg/dL	40 - 54 mg/dL<br>161 - 250 mg/dL	30 - 39 mg/dL<br>251 - 500 mg/dL	<30 mg/dL<br>>500 mg/dL
Triglycerides	-----	400 - 750 mg/dL	751 - 1200 mg/dL	>1200 mg/dL
URIC ACID<br>Hyperuricemia	7.5 - 10.0 mg/dL	10.1 - 12.0 mg/dL	12.1 - 15.0 mg/dL	>15.0 mg/dL
LIVER TRANSAMINASE<br>AST (SGOT)<br>ALT (SGPT)<br>GGT<br>Alk. Phosphatase	1.51 - 2.5 x ULN<br>1.51 - 3.0 x ULN<br>1.51 - 2.5 x ULN<br>1.51 - 2.5 x ULN	>2.5 - 5.0 x ULN<br>>3.0 - 5.0 x ULN<br>>2.5 - 5.0 x ULN<br>>2.5 - 5.0 x ULN	>5.0 - 10.0 x ULN<br>>5.0 - 10.0 x ULN<br>>5.0 - 10.0 x ULN<br>>5.0 - 10.0 x ULN	>10.0 x ULN<br>>10.0 x ULN<br>>10.0 x ULN<br>>10.0 x ULN
PANCREATIC ENZYMES<br>Amylase<br>Pancreatic amylase<br>Lipase	>1.0 - 1.5 x ULN<br>>1.0 - 1.5 x ULN<br>>1.0 - 1.5 x ULN	>1.5 - 2.0 x ULN<br>>1.5 - 2.0 x ULN<br>>1.5 - 2.0 x ULN	>2.0 - 5.0 x ULN<br>>2.0 - 5.0 x ULN<br>>2.0 - 5.0 x ULN	>5.0 x ULN<br>>5.0 x ULN<br>>5.0 x ULN
**URINALYSIS**				
**PARAMETER**	**GRADE 1**<br>***MILD***	**GRADE 2**<br>***MODERATE***	**GRADE 3**<br>***SEVERE***	**GRADE 4**<br>***POTENTIALLY LIFE THREATENING***

Proteinuria<br>Random urine<br><br>24 Hour Urine	1+<br><br>200 mg - 1 g loss/day OR <0.3% OR <3 g/L	2-3+<br><br>1-2 g loss/day OR 0.3 - 1.0% OR 3-10 g/L	4+<br><br>2-3.5 g loss/day OR >1.0% OR >10 g/L	Nephrotic syndrome<br><br>Nephrotic syndrome OR >3.5 g loss/day
Hematuria	Microscopic only ≤10 rbc/hpf	>10 rbc/hpf	Gross, with or without clots OR RBC casts	Obstructive OR transfusion req

WRAIR 1893
PROPRIETARY

**CARDIO-VASCULAR**				
**PARAMETER**	**GRADE 1**<br>***MILD***	**GRADE 2**<br>***MODERATE***	**GRADE 3**<br>***SEVERE***	**GRADE 4**<br>***POTENTIALLY LIFE THREATENING***
Cardiac Arrhythmia	-----	Asymptomatic; transient dysrhythmia, no Rx req	Recurrent/persistent dysrhythmia; symptomatic Rx req	Unstable dysrhythmia, hospitalization and Rx req
Hypertension	Transient, increase >20 mm Hg diastolic BP; no Rx req	Recurrent; chronic increase >20 mm Hg diastolic BP; Rx req	Acute Rx req; outpatient OR hospitalization possible	Hospitalization req OR end organ damage
Hypotension	Transient orthostatic hypotension with heart rate increased by >20 beats/min OR decreased by <10 mm Hg systolic BP, no Rx req	Symptoms OR BP decreased by <20 mm Hg systolic, correctable with oral fluid Rx	IV fluid req OR hospitalization	Mean arterial pressure <60 mm HG OR end organ damage OR shock, vasopressor Rx req
Pericarditis	Minimal effusion	Mild/mod asymptomatic effusion, no Rx	Symptomatic effusion, pain, EKG changes	Tamponade OR pericardiocentesis OR surgery req
Hemorrhage, blood loss	-----	Mildly symptomatic, no Rx req	Gross blood loss OR 1-2 units transfused	Massive blood loss OR >2 units transfused

WRAIR 1893

PROPRIETARY

Nausea	Mild OR transient; reasonable intake maintained	Mod discomfort OR intake decreased for <3 days	Severe discomfort OR minimal intake for ≥3 days_Hospitalization req__Vomiting_Mild OR transient; 2-3 episodes per day OR mild vomiting lasting <1 week_Mod OR persistent; 4-5 episodes per day; OR vomiting lasting ≥1 week_Severe vomiting of all food/fluids in 24 hrs OR orthostatic hypotension OR IV Rx req_Hypotensive shock OR hospitalization req for IV Rx req__Diarrhea_Mild OR transient, 3-4 loose stools per day OR mild diarrhea lasting <1 week_Mod OR persistent; 5-10 loose stools per day OR diarrhea lasting ≥ 1 week_>10 loose stools/day, bloody diarrhea; OR orthostatic hypotension OR electrolyte imbalance, >2 L IV fluid req	Hypotensive shock OR severe electrolyte imbalance
Oral Discomfort/Dysphagia	Mild discomfort, no difficulty swallowing	Difficulty swallowing but able to eat and drink	Unable to swallow solids	Unable to drink fluids; IV fluids req
**RESPIRATORY**				
**PARAMETER**	**GRADE 1**<br>***MILD***	**GRADE 2**<br>***MODERATE***	**GRADE 3**<br>***SEVERE***	**GRADE 4**<br>***POTENTIALLY LIFE THREATENING***
Cough (for aerosol studies)	Transient; no Rx	Treatment associated cough; inhaled bronchodilator	Uncontrolled cough; systemic Rx req	-----

Bronchospasm Acute	Transient; no Rx; FEV1 or peak flow reduced to 70% - 80%	Rx req; normalizes with bronchodilator; FEV1 or peak flow 50%-69%	No normalization with bronchodilator; FEV1 or peak flow 25%-49%, retractions	Cyanosis; FEV1 or peak flow <25% OR intubated
Dyspnea	Dyspnea on exertion	Dyspnea with normal activity	Dyspnea at rest	Dyspnea requiring O_2 therapy
**NEUROLOGIC**				
**PARAMETER**	**GRADE 1**<br>***MILD***	**GRADE 2**<br>***MODERATE***	**GRADE 3**<br>***SEVERE***	**GRADE 4**<br>***POTENTIALLY LIFE THREATENING***
Neuro-cerebellar	Slight incoordination OR dysdiadochokinesia	Intention tremor OR dysmetria OR slurred speech OR nystagmus	Ataxis requiring assistance to walk or arm incoordination interfering with ADLs	Unable to stand
Neuro-psych/mood	-----	-----	Severe mood changes requiring medical intervention; suicidal ideation	Acute psychosis req hospitalization; suicidal gesture/attempts
Paresthesia (burning, tingling, *etc.*)	Mild discomfort; no Rx req	Mod discomfort; non-narcotic analgesia req	Severe discomfort; OR narcotic analgesia req with symptomatic improvement	Incapacitating; OR not responsive to narcotic analgesia
Neuro-motor	Mild weakness in muscle of feet but able to walk and/or mild increase or decrease in reflexes	Mod weakness in feet (unable to walk on heels and/or toes), mild weakness in hands, still able to do most hand tasks and/or loss of previously present reflex or development of hyperreflexia and/or unable to do deep knee bends due to weakness	Marked distal weakness (unable to dorsiflex toes or foot drop, and mod proximal weakness *e.g.*, in hands interfering with ADLs and/or req assistance to walk and/or unable to rise from chair unassisted	Confined to bed or wheel chair because of muscle weakness

Neuro-sensory	Mild impairment (decreased sensation, *e.g.*, vibratory, pinprick, hot/cold in great toes) in focal area or symmetrical distribution	Mod impairment (mod decreased sensation, *e.g.*, vibratory, pinprick, hot/cold to ankles) and/or joint position or mild impairment that is not symmetrical	Severe impairment (decreased or loss of sensation to knees or wrists) or loss of sensation of at least mod degree in multiple different body areas (*i.e.*, upper and lower extremities)	Sensory loss involves limbs and trunk
**MUSCULO-SKELETAL**				
**PARAMETER**	**GRADE 1**<br>***MILD***	**GRADE 2**<br>***MODERATE***	**GRADE 3**<br>***SEVERE***	**GRADE 4**<br>***POTENTIALLY LIFE THREATENING***
Arthralgia/Arthritis	Arthralgia	Arthralgia with joint effusion or moderate impairment of activity	Frank arthritis with or without effusion OR resulting in severe impairment of activity	-----
Myalgia	Myalgia without limitation of activity	Muscle tenderness at other than injection site or with moderate impairment of activity	Frank myonecrosis OR with severe impairment of activity	-----
**CUTANEOUS**				
**PARAMETER**	**GRADE 1**<br>***MILD***	**GRADE 2**<br>***MODERATE***	**GRADE 3**<br>***SEVERE***	**GRADE 4**<br>***POTENTIALLY LIFE THREATENING***
Rash/Dermatitis	Erythema, pruritus	Diffuse maculopapular rash OR dry desquamation	Vesiculation OR moist desquamation OR ulceration	ANY ONE: mucous membrane involvement, suspected Stevens-Johnson (TEN), erythema multiforme, necrosis req surgery, exfoliative dermatitis
Local Reaction	Erythema OR induration <15 x 15 cm (225 cm^2)	Erythema, induration, or edema >15 x 15 cm (225 cm^2)	Ulceration OR super infection OR phlebitis	Necrosis of the skin
**MISCELLANEOUS**				

PARAMETER	GRADE 1<br>***MILD***	GRADE 2<br>***MODERATE***	GRADE 3<br>***SEVERE***	GRADE 4<br>***POTENTIALLY LIFE THREATENING***
Fever<br>oral >12 hours	37.7 - 38.9C<br>(100.0 - 101.5F)	39.0 - 39.5C<br>(101.6 - 102.9F) OR max temp of 103F	39.6 - 40.5C<br>(103 - 105F) OR max temp of 103.5F	>40.5C (105F) OR max temp >105F
Headache	Mild, no Rx req, OR non-narcotic analgesia Rx	Mod; OR responds to initial narcotic Rx	Severe; intractable; OR req repeated narcotic Rx	Req hospitalization, associated with neurologic, respiratory or cardiovascular abnormalities
Allergic Reaction	Pruritus without rash at injection site	Localized urticaria at injection site	Generalized urticaria angioedema	Anaphylaxis
ADL<br>(Activities of Daily Living)	Normal activity reduced <48 hours	Normal activity reduced 25%-50% >48 hours	Normal activity reduced >50%; cannot work >48 hours	Unable to care for self
Eye		Mild pain, visual changes, conjunctival erythema, abnormal slit lamp	Loss of vision, clinically diagnosed uveitis, moderate to severe pain, glaucoma	-----
Constipation		Moderate abdominal pain 78 hours with impaction require output prescription	Requiring disimpaction or hospital treatment	Distention with vomiting OR obstipation

17.8 VACCINE PREPARATION

Refer to the DVC Study Specific Procedure # 01-rPA-EC-02

17.9 LABEL DESCRIPTION OF INVESTIGATIONAL PRODUCT

Therapore rPA (0.3mg) vial label *

*Note: This label contains an error that has been documented in DVC's manufacturing files. It should read Therapore rPA (0.3mg per ml).

Therapore rPA (0.1mg per ml) vial label

18.0 VOLUNTEER REGISTRY DATA SHEET (USAMRDC 60-R)

VOLUNTEER REGISTRY DATA SHEET (USAMRDC 60-R)

THIS FORM IS AFFECTED BY THE PRIVACY ACT OF 1974

1. **AUTHORITY: 5 USC 301; 10 USC 1071-1090; 44 USC 3101; EO 9397**

2. **Principal and Routine Purposes: To document participation in research conducted or sponsored by the U.S. Army Medical Research and Materiel Command. Personal information will be used for identification and location of participants.**

3. **Mandatory or Voluntary Disclosure: The furnishing of the SSN is mandatory and necessary to provide identification and to contact you if future information indicates**

that your health may be adversely affected. Failure to provide information may preclude your participation in the research study.

PART A - INVESTIGATOR INFORMATION
(To Be Completed By Investigator)

PLEASE PRINT, USING INK OR BALLPOINT PEN

1. Study Number:_________________ **2. Protocol Title:**

3. Contractor (Laboratory / Institute Conducting Study):

4. Study Period: From: _____/_____/_____ **To: ______/______/______**
DD MM YY **DD MM YY**

5. Principal / Other Investigator(s) Names(s): **6. Location / Laboratory**

1. _____________________________________ **________________/______________**
2. _____________________________________ **________________/______________**
3. ______________________________________ **________________/______________**

PROPRIETARY

VOLUNTEER REGISTRY DATA SHEET (USAMRDC 60-R) (Continued)

PART B - VOLUNTEER INFORMATION
(To Be Completed by Volunteer)

PLEASE PRINT, USING INK OR BALLPOINT PEN
7. SSN: _______/______/______ 8. Name: ____

1. Sex: M___F___ 10. Date of Birth: ____/____/____ 11: *MOS/Job Series___ 12:Rank/Grade __

13. Permanent Home Address (Home of Record) or Study Location:

____
(Street) **(P.O. Box / Apartment Number)**

____
(City) **(Country)** **(State)** **(Zip Code)**

Permanent Home Phone Number: __________________________________

14. * Local Address (If Different From Permanent Address):

____
(Street) **(P.O. Box / Apartment Number)**

____
(City) **(Country)** **(State)** **(Zip Code)**

Local Phone Number: ____

15. * Military Unit: ___________________________________Zip Code: _________________

Organization: _______________ Post: _____________Duty Phone Number: _____________

VOLUNTEER REGISTRY DATA SHEET (USAMRDC 60-R) (Continued)

PART C - ADDITIONAL INFORMATION
(To Be Completed by Investigator)

PLEASE PRINT, USING INK OR BALLPOINT PEN
16. Location of Study: ____
17. Is Study Completed: Y: _______ N: _______

Did volunteer finish participation: Y: _____ N: ____ If YES, date finished / /
DD MM YY
If NO, date withdrawn: / / Reason Withdrawn:
DD MM YY
18. Did any Serious or Unexpected Adverse Incident or Reaction Occur: Y: N:
If YES, Explain:

19. * Volunteer Follow-up:____

Purpose:____

Date: _____/____/_____ Was contact made: Y: ______ N: _____ If no action taken, explain:

20. * Hard Copy Records Retired: Place: ______________________ File NR: ____________

21. * Product Information:
Product: ____

Manufacturer: ____

Lot #: ______________________________ Expiration Date: _________________________

NDA #: ______________________________ IND/IDE #______________________________

***Indicates that item may be left blank if information is unavailable or does not apply. Entries must be made for all other items.**
When completed, a copy of this form should be sent to the address below:
Commander
U.S. Army Medical Research and Materiel Command
ATTN: MCMR-RCQ-HR
Fort Detrick, MD 21702-5012
